# Gold(iii) promoted formation of dihydroquinazolinones: double X–H activation by gold†

**DOI:** 10.1039/d0ra06537d

**Published:** 2020-09-28

**Authors:** Veerabhushanam Kadiyala, Perla Bharath Kumar, Komalla Sunil, Chittala Emmaniel Raju, Balasubramanian Sridhar, Galla V. Karunakar

**Affiliations:** Fluoro and Agrochemicals Department, CSIR-Indian Institute of Chemical Technology Hyderabad 500007 India gallavk@iict.res.in; Academy of Scientific and Innovative Research Ghaziabad 201002 India; Center for X-ray Crystallography, CSIR-Indian Institute of Chemical Technology Hyderabad 500007 India

## Abstract

An efficient 2-furyl gold–carbene promoted synthetic method was developed for the formation of dihydroquinazolinones from enynones by dual insertion of anthranilamides. In this organic transformation a new C–O and two C–N bond formations occurred and dihydroquinazolinones were obtained with a quaternary centre in moderate to very good yields in one-pot synthesis.

## Abstract 2

An efficient 2-furyl gold–carbene promoted synthetic method was developed for the formation of dihydroquinazolinones from enynones by dual insertion of anthranilamides in very good yields.

## Graphical abstract

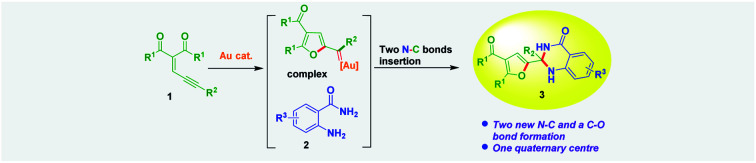

## Introduction

**Fig. 1:**
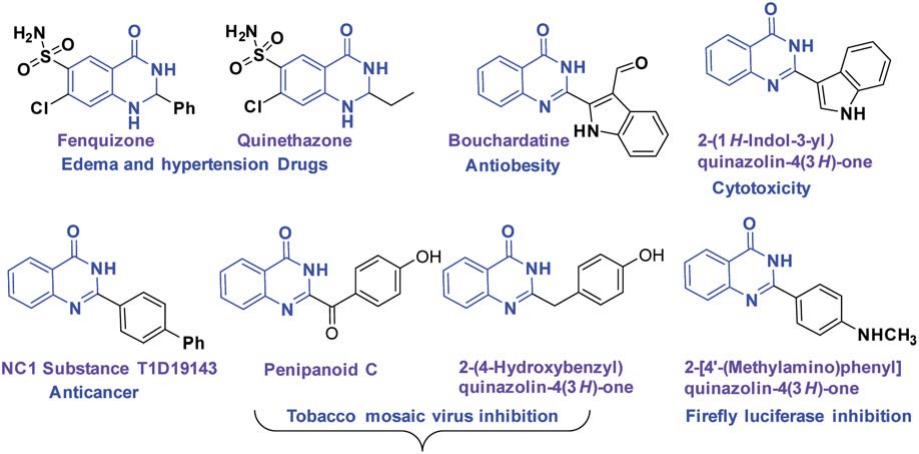
Selected examples of important molecules containing dihydroquinazolinone core skeleton.

Nitrogen-containing heterocyclic molecules^1^ such as quinazolinones have gained much attention due to their wide range of biological and pharmacological applications.^2^ Dihydroquinazolinone derivatives like fenquizone,^3^ and quinethazone^4^ are drugs for edema and hypertension. It was reported that bouchardatine exhibits antiobesity activitity,^5^ and penipanoid C exhibits tobacco mosaic virus inhibition^6^ (Fig. 1). Further, substituted dihydroquinazolinone derivatives displayed significant cytotoxic activity.^7^

Hence, the development of new synthetic methods for the formation of dihydroquinazolinones is a limitless frontier. Cooperative catalysis^8^ has been established as a handy tool for the synthesis of several biologically valuable molecules and different procedures were reported for the synthesis of dihydroquinazolinone derivatives.^9^ Exploration of gold-catalyzed^10^ organic transformations has attracted much attention in recent years due to their broad functional group tolerance and selectivity for the formation of valuable heterocyclic molecules in one-pot reaction conditions.^11^ The recent literature indicating that exploitation of enynal/enynone has recognised as good donor or donor–donor carbene precursors for C–H/X–H insertion and cyclopropanation reactions.^12^ Several reports are available for synthesis of substituted furans from enynones in presence of metal catalysts *via* a 5-*exo*-dig cyclization.^13^

**Scheme 1:**
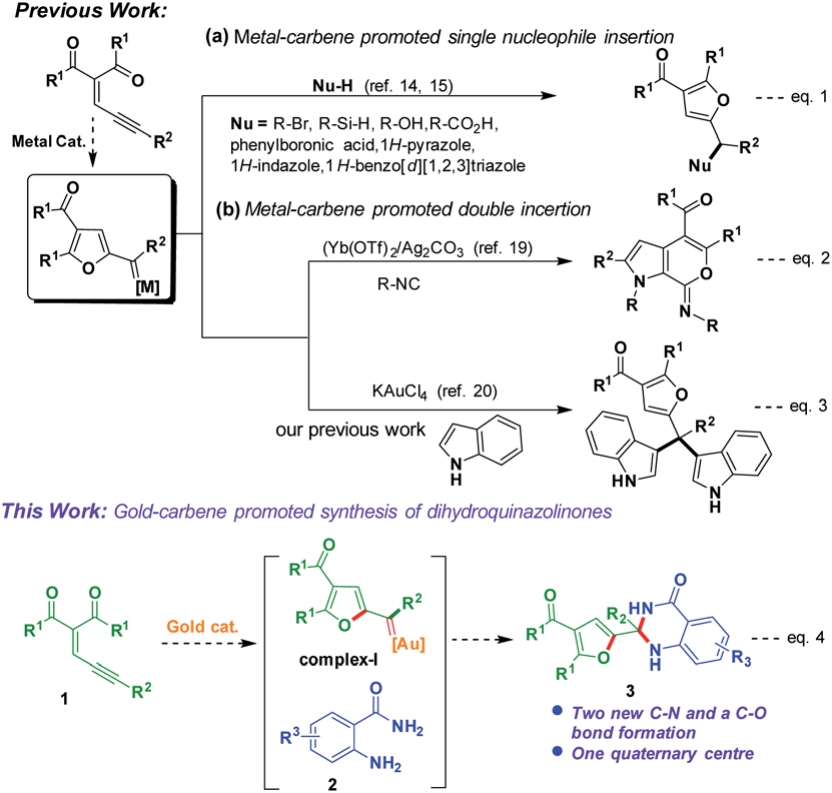
Synthetic transformations of enynones.

The reaction mechanism was proposed *via* (2-furyl) metal–carbene^14^ intermediate would react with one nucleophile^15^ to produce addition products (Scheme 1, eqn (1)). López and Vicente co-workers reported a method for synthesis of functionalized furans from enynones in the presence of zinc catalyst.^16^ Recently, Zhu *et al.* developed metal carbene promoted method for synthesis of vinyl-substituted dihydroindoles.^17^ Hashmi and co-workers studied the stabilization effects of gold carbene complexes.^18^

Double insertion of isocyanides to enynones produced pyrrole-fused heterocyclic molecules *via* (2-furyl) metal–carbene intermediate was reported by Jia and Li co-workers (Scheme 1, eqn (2)).^19^ Very recently, we have reported formation of tetraarylmethane derivatives by reaction of enynones with indoles *via* (2-furyl) gold–carbene intermediate (Scheme 1, eqn (3)).^20^ Our current research^21^ efforts focused to explore the reactivity of enynones under gold catalysis. We envisioned that reaction of enynones (1) in the presence of gold-catalyst would produce gold–carbene complex-I, which would react with anthranilamide (2) may give corresponding dihydroquinazolinone derivative 3 (Scheme 1, eqn (4)).

## Results and discussion

**Scheme 2:**
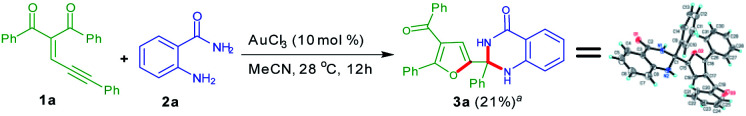
Reaction of enynone (1a) with anthranilamides (2a) for formation of 3a^*a*^ [CCDC 1863534].^22^

Accordingly, we have conducted an experiment by using substrates 1a and 2a in the presence of AuCl_3_ (Scheme 2). Very interestingly, 21% yield of the corresponding product 2-(4-benzoyl-5-phenylfuran-2-yl)-2-phenyl-2,3-dihydroquinazolin-4(1*H*)-one 3a was observed. The product 3a was further confirmed by single crystal X-ray analysis.^22^ It is noteworthy that in this organic transformation two C–N bonds were formed by dual insertion of anthranilamide with a quaternary centre. This interesting observation encouraged us to optimize this reaction to get the better yields of the product 3a.

Gold(i) catalysts were screened with the substrates 1a with 2a to produce moderate yields of 3a along with 3a′ (Table 1, entries 1 and 2). Whereas when experiments were conducted in the presence of AuBr_3_ and KAuCl_4_ moderate yields of product 3a was observed (Table 1, entries 3 and 4). Complex mixture was obtained while reaction was performed in the presence of IPrAuCl (Table 1, entry 5). In the presence of AuClPPh_3_, poor yields of desired product 3a was found (Table 1, entry 6). Then reactions were conducted by utilizing gold catalysts in combination of selectfluor (Table 1, entries 7–10), yields of desired product 3a was not improved. Reactions were performed by employing KAuCl_4_ in combination with K_2_S_2_O_8_, CF_3_COOH, Cu(OAc)_2_, K_2_CO_3_, pyridine *N*-oxide and PhI(OAc)_2_ (Table 1, entries 11–16), moderate yield of 3a was observed. Nevertheless, KAuCl_4_ and FeCl_3_ combination afforded very good yield of (81%) of product 3a (Table 1, entry 17). An experiment was conducted by utilizing only FeCl_3_, poor yields of product 3a was observed along with 3a′ (Table 1, entry 18). Reactions were screened by using series of solvents like toluene, MeOH, THF, DMF and DCE, none of them gave better yield than MeCN (Table 1, entries 19–23). When the gold-catalyst loading decreased from 10 mol% to 5 mol% and 7 mol%, the product yield also reduced to 52% and 58%, respectively (Table 1, entries 24 and 25). Two reactions were conducted without utilizing FeCl_3_ and these cases poor yields of product 3a observed (Table 1, entries 26 and 27).

**Table 1:** Optimization of the reaction conditions^a^

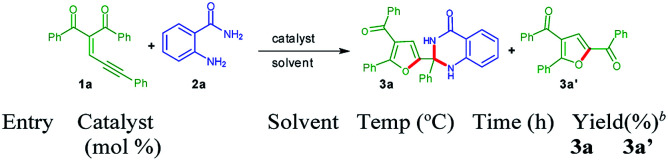						
Entry	Catalyst (mol%)	Solvent	Temp (°C)	Time (h)	Yield^b^ (%)	
					3a	3a′
1	[Au(JohnPhos)(MeCN)][SbF_6_](10)	MeCN	28	12	45	48
2	(C_20_H_15_AuF_6_NO_4_PS_2_)_2_C_7_H_8_ (10)	MeCN	28	12	46	43
3	AuBr_3_ (10)	MeCN	28	12	42	—
4	KAuCl_4_ (10)	MeCN	28	12	35	—
5	IPrAuCl (10)	MeCN	100	12	cm^c^	
6	AuClPPh_3_ (10)	MeCN	70	24	32	—
7	AuClPPh_3_ (10), Selectfluor (20)	MeCN	70	24	30	—
8	IPrAuCl (10), Selectfluor (20)	MeCN	70	24	cm^c^	
9	KAuCl_4_ (10), Selectfluor (20)	MeCN	70	24	49	—
10	AuCl_3_ (10), Selectfluor (20)	MeCN	70	24	48	—
11	KAuCl_4_ (10), K_2_S_2_O_8_ (20)	MeCN	28	14	49	—
12	KAuCl_4_ (10), CF_3_COOH (1 eq.)	MeCN	28	14	cm^c^	
13	KAuCl_4_ (10), Cu(OAc)_2_ (20)	MeCN	28	14	40	—
14	KAuCl_4_ (10), K_2_CO_3_ (1 eq.)	MeCN	28	14	45	—
15	KAuCl_4_ (10), Py *N*-oxide (1.2 eq.)	MeCN	28	14	10	—
16	KAuCl_4_ (10), PhI(OAc)_2_ (1.5 eq.)	MeCN	28	14	20	—
**17**	**KAuCl**_**4**_ **(10), FeCl**_**3**_ **(2.0 eq.)**	**MeCN**	**80**	**05**	**81**	**11**
18	FeCl_3_ (2.0 eq.)	MeCN	80	12	20	6
19	KAuCl_4_ (10), FeCl_3_ (2.0 eq.)	Toluene	80	12	20	32
20	KAuCl_4_ (10), FeCl_3_ (2.0 eq.)	MeOH	80	12	30	35
21	KAuCl_4_ (10), FeCl_3_ (2.0 eq.)	THF	80	12	48	20
22	KAuCl_4_ (10), FeCl_3_ (2.0 eq.)	DMF	80	12	46	25
23	KAuCl_4_ (10), FeCl_3_ (2.0 eq.)	DCE	80	12	41	28
24	KAuCl_4_ (5), FeCl_3_ (1.0 eq.)	MeCN	80	12	52	21
25	KAuCl_4_ (7), FeCl_3_ (1.5 eq.)	MeCN	80	08	58	18
26^d^	AgSbF_6_ (10)	MeCN	80	48	12	8
27^d^	AuCl_3_ (10)	MeCN	80	36	47	—

a Reaction conditions: all reactions were carried out under nitrogen atmosphere with 1a (0.15 mmol), and 2a (0.225 mmol) and solvent (2 mL) in oil bath.

b Yields are for isolated products; eq.: equivalent.

c cm: complex mixture.

d Entries 26 and 27 were conducted without FeCl_3_.^23^

The above experiments concludes that Table 1, entry 17 is the best suitable reaction conditions. Then substrate scope was tested by utilizing different enynones (1a–k) with anthranilamide 2a under the optimal conditions. These results are incorporated in the Table 2.

**Table 2:** Scope of substituted dihydroquinazolinones (3)^a^

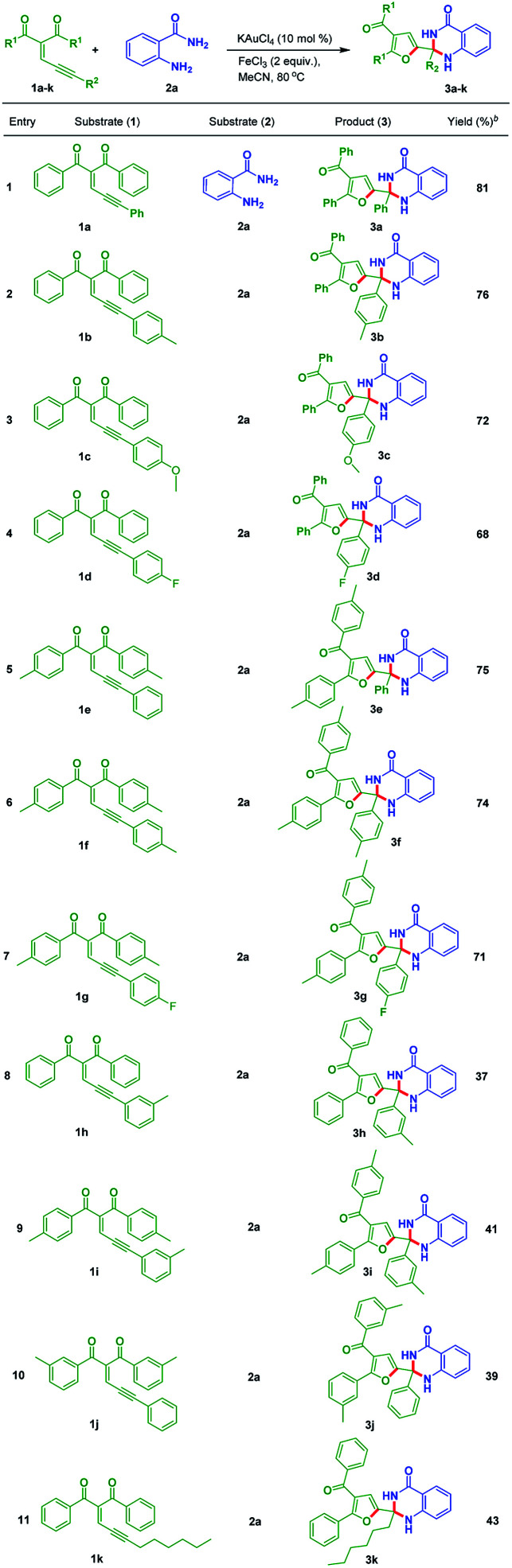

a Reaction conditions: all reactions were carried out at 80 °C under nitrogen atmosphere with 1 (1.0 equiv.), and 2a (1.5 equiv.) in the presence of KAuCl_4_ (10 mol%), FeCl_3_ (2.0 equiv.) and solvent (3 mL) in oil bath; yields are for isolated products.

The substrates which are bearing electron-donating groups such as 1b and 1c were tested with 2a to provide 76% and 72% yields of corresponding products 3b and 3c, respectively. Electron-withdrawing functional group containing enynone such as 1d react with 2a to give the corresponding dihydroquinazolinone derivative 3d in 68% yield. Substrates bearing electron-donating groups like 1e and 1f reacted with 2a to produce 75% and 74% yields of the corresponding dihydroquinazolinone derivatives 3e and 3f, respectively. Both electron-donating and electron-withdrawing functional groups containing enynone like 1g reacted with 2a to generate the corresponding product 3g in 71% yield. The substrates which are having electron-donating groups at *ortho* position of R^2^ like 1h (R^2^ = 3-Me-C_6_H_4_), 1i (R^2^ = 3-Me-C_6_H_4,_ R^1^ = 4-Me-C_6_H_4_) and 1j (R^1^ = 3-Me-C_6_H_4_) reacted with 2a to provide the corresponding products 3h, 3i and 3j in moderate yields, respectively (Table 2, entries 8–10). Alkyl substitution at R^2^ position containing substrate like 1k produced the product 3k in 43% yield (Table 2, entry 11).

Further, experiments were conducted to check the scope of dihydroquinazolinone derivatives by utilizing different substituted anthranilamides (2b–e) with enynones (1a–f). These results were included in the Table 3. The enynone 1a was tested with electron-donating functional group containing anthranilamide 2b to give 3l in 72% yield. Electron-withdrawing functional group containing anthranilamides such as 2c, 2d, and 2e reacted with 1a to produce the corresponding dihydroquinazolinone derivatives 3m, 3n, and 3o in 65%, 63% and 60% yields, respectively (Table 3, entries 2–4). Enynones bearing electron donating groups such as 1b and 1c reacted with 2b to provide corresponding products 3p and 3q in 72% and 70% yields, respectively (Table 3, entries 5 and 6). Fluorine substituted enynone such as 1d reacted with 2b, 2c, 2d and 2e to provide the corresponding dihydroquinazolinone derivatives such as 3r, 3s, 3t and 3u in 69%, 63%, 61% and 62% yields, respectively (Table 3, entries 7 and 10). Electron-donating substitutions containing enynones such as 1e and 1f reacted with 2b under optimized reaction conditions to give the corresponding products 3v and 3w in 70% and 68% yields, respectively (Table 3, entries 11 and 12).

**Table 3:** Scope of substituted dihydroquinazolinones (3)^a^

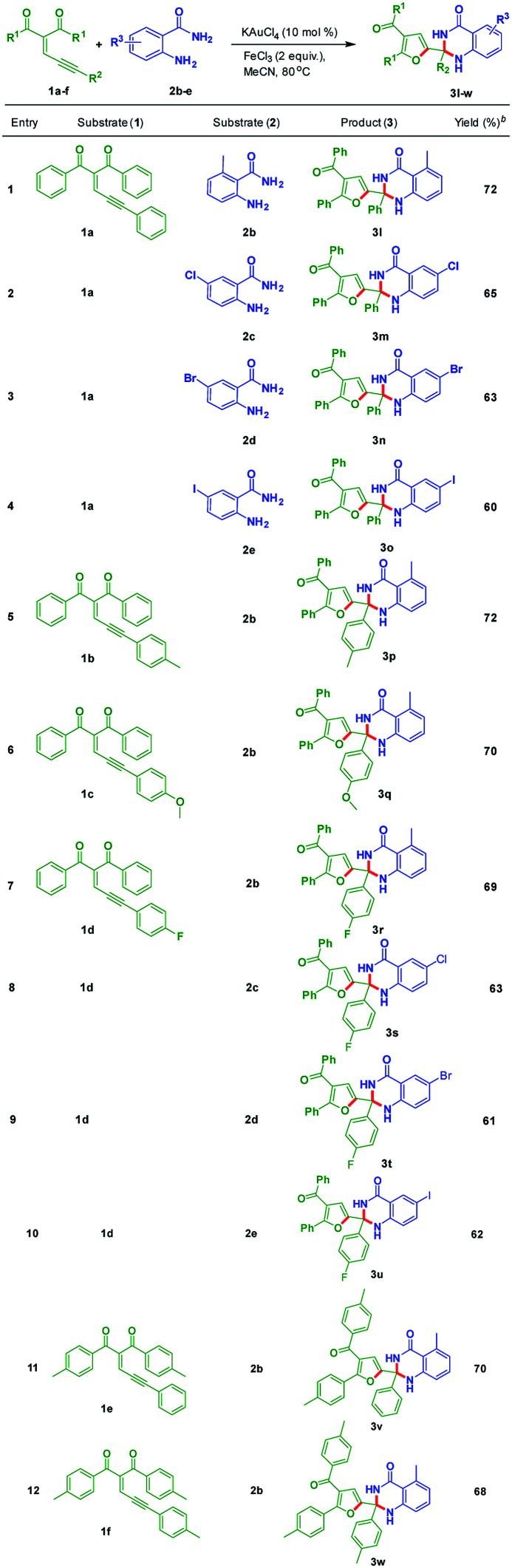

a Reaction conditions: all reactions were carried out at 80 °C under nitrogen atmosphere with 1 (1.0 equiv.), and 2 (1.5 equiv.) in the presence of KAuCl_4_ (10 mol%), FeCl_3_ (2.0 equiv.) and solvent (3 mL) in oil bath; yields are for isolated products 3.

**Scheme 3:**
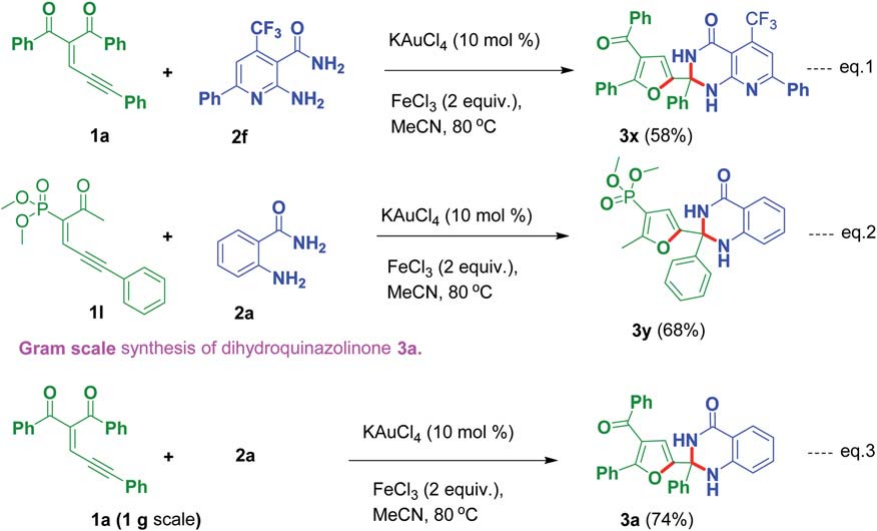
(a) Reaction of 1a with heteroaryl amine 2f (eqn (1)); (b) reaction of dimethyl-(2-oxo-6-phenylhex-3-en-5-yn-3-yl)phosphonate 1l with 2a (eqn (2)); (c) gram scale synthesis of product 3a (eqn (3)).

2-Amino-6-phenyl-4-(trifluoromethyl)nicotinamide 2f reacted with 1a to produce the corresponding product 3t in 58% yield (Scheme 3, eqn (1)). An experiment was conducted by employing a phosphorus substituted enynone like 1l with 2a to provide the corresponding dihydroquinazolinone derivative 3y in 68% yield (Scheme 3, eqn (2)). Further, one more experiment was conducted in gram scale by utilizing 1a and 2a under optimized reaction conditions to give the corresponding product 3a in 74% yield (Scheme 3, eqn (3)).

**Scheme 4:**
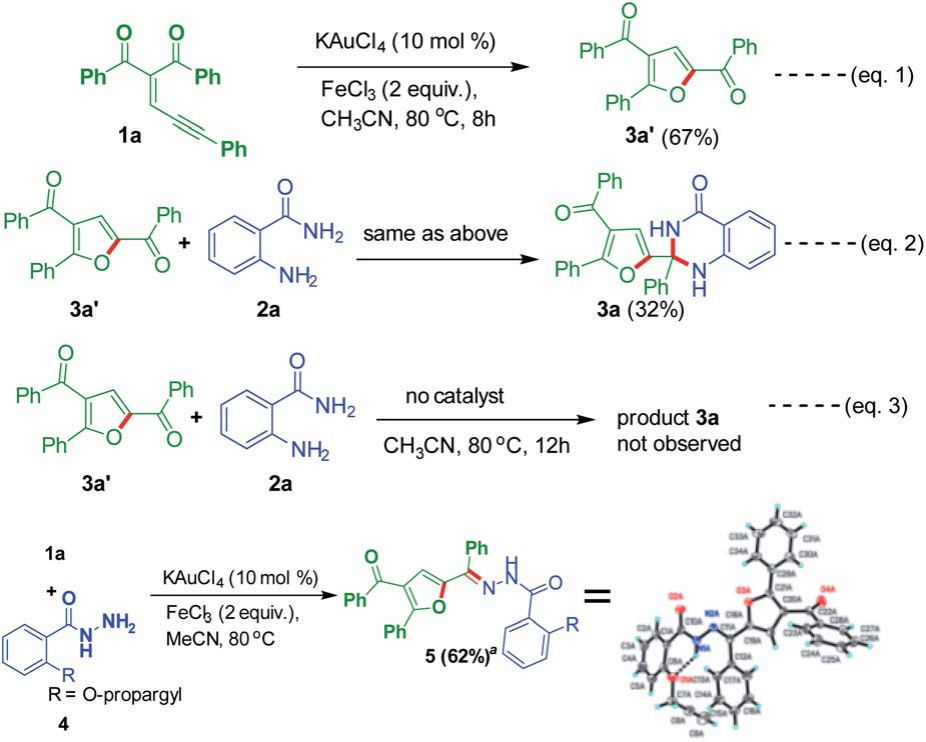
Control experiments. ^*a*^Compound 5 CCDC 1898773.^22^

Control experiments were conducted to clarify the reaction mechanism (Scheme 4). The substrate 1a was tested under optimized conditions to produce good yields of product 3a′ (Scheme 4, eqn (1)). A reaction was conducted by utilizing 3a′ with anthranilamide 2a in the presence of gold-catalyst to provide 32% yield of product 3a (Scheme 4, eqn (2)). Without using catalyst one reaction was conducted by using 3a′ and 2a, in this case product 3a was not observed (Scheme 4, eqn (3)). Then 1a was tested with 2-(prop-2-yn-1-yloxy)benzohydrazide 4 to give 62% yield of product 5. The structure of the compound 5a further charaecterized by single crystal X-ray analysis.^22^

**Scheme 5:**
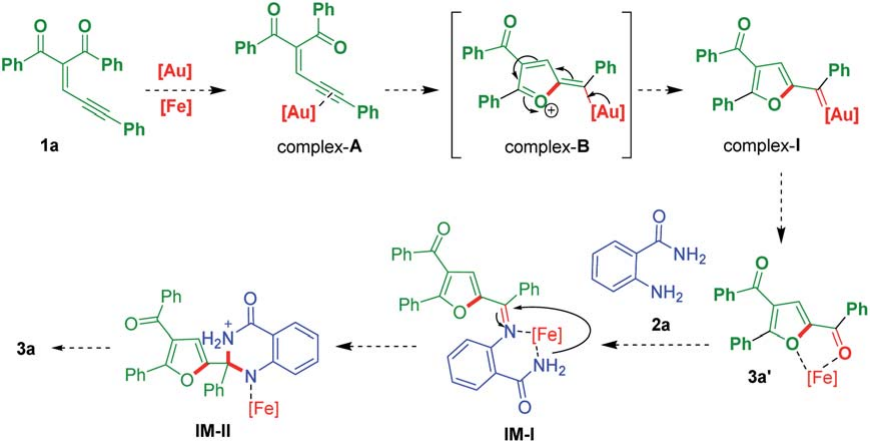
A plausible reaction mechanism.

Formation of dihydroquinazolinones can be proposed by the reaction mechanism as shown in Scheme 5. Gold catalyst would coordinate with enynone 1a may form complex-A, which would further generate 2-furyl gold carbene complex-I*via* intramolecular of 5-*exo*-dig cyclised zwitterionic complex-B.^24^ Then, it would produce ketone (3a′),^13,14^ which would coordinate with ferric chloride as a lewis acid in a regioselective fashion then it would reacts with anthranilamide (2a) may generate IM-I. Subsequent activation of IM-I by metal catalyst may lead to cyclization to form intermediate IM-II, which would finally afford the product 3a.

## Conclusion

In conclusion, we have established gold-catalyzed reaction of enynones with dual insertion of anthranilamides to produce a novel approach for synthesis of dihydroquinazolinones. It is significant that in this organic transformation new C–O and two C–N bonds were formed with a quaternary centre with good functional group tolerance.

## Experimental section

### General information

Reactions were carried out in oven dried reaction flasks under nitrogen atmosphere and also solvents and reagents were transferred by oven-dried syringes to ambient temperature. TLC was performed on Merck silica gel aluminium sheets using UV as a visualizing agent. Solvents were removed under reduced pressure. Columns were packed as slurry of silica gel in hexane and ethyl acetate solvent mixture. The elution was assisted by applying pressure with an air pump. ^13^C NMR spectra were recorded on 75, 100 and 125 MHz spectrometers. ^1^HNMR spectra were recorded on 300, 400 and 500 MHz spectrometers in appropriate solvents using TMS as internal standard. The following abbreviations were used to explain multiplicities: s = singlet, d = doublet, dd = double doublet, dt = doublet of triplet, td = triplet of doublet, t = triplet, m = multiplet, br s = broad singlet. All reactions were performed under nitrogen atmosphere with freshly distilled and dried solvents. All solvents were distilled using standard procedures. Unless otherwise noted, reagents were obtained from Aldrich, Alfa Aesar, and TCI used without further purification. Synthesis of enynones (1a–l) were prepared by following reported procedures.^25^

### General procedure for synthesis of dihydroquinazolinone derivatives (3a)

To a 10 mL round-bottomed flask equipped with magnetic stir bar the substrate 2-aminobenzamide 2a (0.45 mmol, 61 mg, 1.5 equiv.) was taken and dissolved in dry CH_3_CN (3 mL) at 80 °C (oil bath) after that 1,3-diphenyl-2-(3-phenylprop-2-yn-1-ylidene)propane-1,3-dione 1a (0.3 mmol, 100 mg, 1 equiv.) was added. To this reaction mixture KAuCl_4_ (10 mol%, 11 mg) and FeCl_3_ (0.6 mmol, 97 mg, 2.0 equiv.) was added and stirred at 80 °C for 5 h under nitrogen atmosphere. Progress of the reaction was monitored by using TLC. After completion of the reaction, the reaction mixture was filtered through celite plug and washed with ethyl acetate. The ethyl acetate layer was concentrated under reduced pressure to get crude residue which was purified by column chromatography through silica gel using hexane and ethyl acetate as eluent (10 : 3.5) to give 113 mg of the product 2-(4-benzoyl-5-phenylfuran-2-yl)-2-phenyl-2,3-dihydroquinazolin-4(1*H*)-one 3a (81% yield). The same reaction was conducted on a gram scale by utilizing 1a (1 g) and 2a (0.61 g) produced the corresponding product 3a in 74% yield (1.03 g). A similar experimental procedure was adopted for the synthesis of all the furan containing dihydroquinazolinones (3b–y) and 5.

#### 2-(4-Benzoyl-5-phenylfuran-2-yl)-2-phenyl-2,3-dihydroquinazolin-4(1*H*)-one (3a)

*R*_f_: 0.5; hexane : ethyl acetate mixture (10 : 3.5); yellow solid with 113 mg (81%) yield; melting point: 188–190 °C; ^1^H NMR (500 MHz, CDCl_3_): *δ* 7.93 (dd, *J* = 7.7, 1.2 Hz, 1H), 7.73–7.66 (m, 2H), 7.63–7.56 (m, 4H), 7.52–7.48 (m, 1H), 7.47–7.42 (m, 3H), 7.37–7.31 (m, 3H), 7.30–7.26 (m, 3H), 6.97–6.87 (m, 1H), 6.79–6.70 (m, 2H), 6.35 (s, 1H), 5.09 (br s, 1H), ppm; ^13^C NMR (100 MHz, CDCl_3_): *δ* 191.1, 164.0, 156.3, 153.1, 145.2, 140.4, 137.5, 134.4, 133.0, 129.7, 129.6, 129.3, 129.0, 128.8, 128.4, 128.3, 128.2, 127.4, 126.9, 120.9, 119.8, 115.1, 114.9, 114.0, 72.7 ppm; IR(KBr): *ν* = 3368, 3057, 2922, 1654, 1613, 1485, 1367, 1262 cm^−1^; HRMS (ESI-TOF) *m*/*z*: [M + H]^+^ calcd for C_31_H_21_N_2_O_3_H 471.1703, found 471.1705.

#### Crystal data for 3a

C_31_H_22_N_2_O_3_ (*M* =470.50 g mol^−1^): triclinic, space group *P*1̄ (no. 2), *a* = 8.2197(2) Å, *b* = 10.4608(2) Å, *c* = 14.4752(3) Å, *α* = 77.0948(8)°, *β* = 79.6924(8)°, *γ* = 81.5655(9)°, *V* = 1186.21(4) Å^3^, *Z* = 2, *T* = 294.15 K, *μ*(MoKα) = 0.085 mm^−1^, *D*_calc_ = 1.317 g cm^−3^, 35 683 reflections measured (4.464° ≤ 2*Θ* ≤ 61.018°), 7216 unique (*R*_int_ = 0.0618, *R*_sigma_ = 0.0518) which were used in all calculations. The final *R*_1_ was 0.0634 (*I* > 2*σ*(*I*)) and w*R*_2_ was 0.1669 (all data). CCDC 1863534.

#### 2-(4-Benzoyl-5-phenylfuran-2-yl)-2-(*p*-tolyl)-2,3-dihydroquinazolin-4(1*H*)-one (3b)

Following the general procedure, 100 mg (0.285 mmol, 1.0 equiv.) of 1b, 58 mg (0.428 mmol, 1.5 equiv.) of 2a, 10 mg (10 mol%) of KAuCl_4_ and 92 mg (0.571 mmol, 2.0 equiv.) of FeCl_3_ was used and the reaction time was 12 h. After flash column chromatography on silica gel (eluted with *R*_f_: 0.5; hexane/ethyl acetate mixture 10/3.5), 105 mg of 3b was obtained in 76% yield as a yellow solid. Mp: 166–168 °C; ^1^H NMR (500 MHz, CDCl_3_): *δ* 7.92 (dd, *J* = 7.8, 1.2 Hz, 1H), 7.74–7.65 (m, 2H), 7.64–7.56 (m, 2H), 7.53–7.43 (m, 3H), 7.39–7.31 (m, 3H), 7.30–7.21 (m, 5H), 6.94–6.87 (m, 1H), 6.75–6.70 (m, 2H), 6.31 (s, 1H), 5.06 (br s, 1H), 2.38 (s, 3H) ppm; ^13^C NMR (100 MHz, CDCl_3_): *δ* 191.1, 163.8, 156.2, 153.4, 145.3, 139.7, 137.6, 137.5, 134.2, 132.9, 129.6, 129.4, 129.2, 129.0, 128.37, 128.31, 128.2, 127.4, 126.8, 120.9, 119.7, 115.1, 114.8, 113.8, 72.5, 21.0 ppm; IR(KBr): *ν* = 3376, 3068, 2922, 1654, 1612, 1484, 1368, 1267 cm^−1^; HRMS (ESI-TOF) *m*/*z*: [M + H]^+^ calcd for C_32_H_23_N_2_O_3_H 485.1859, found 485.1862.

#### 2-(4-Benzoyl-5-phenylfuran-2-yl)-2-(4-methoxyphenyl)-2,3-dihydroquinazolin-4(1*H*)-one (3c)

Following the general procedure, 100 mg (0.273 mmol, 1.0 equiv.) of 1c, 55 mg (0.409 mmol, 1.5 equiv.) of 2a, 10 mg (10 mol%) of KAuCl_4_ and 88 mg (0.546 mmol, 2.0 equiv.) of FeCl_3_ was used and the reaction time was 12 h. After flash column chromatography on silica gel (eluted with *R*_f_: 0.5; hexane/ethyl acetate mixture 10/3.5), 98 mg of 3c was obtained in 72% yield as a yellow solid. Mp: 111–113 °C; ^1^H NMR (400 MHz, CDCl_3_): *δ* 7.92 (d, *J* = 7.3 Hz, 1H), 7.75–7.46 (m, 6H),7.39–7.23 (m, 7H), 7.01–6.88 (m, 3H), 6.79–6.65 (m, 2H), 6.28 (s, 1H), 5.04 (br s, 1H), 3.83 (s, 3H), ppm; ^13^C NMR (100 MHz, CDCl_3_): *δ*191.1, 163.9, 160.5, 156.3, 153.3, 145.3, 137.5, 134.3, 133.0, 132.5, 129.1, 128.4, 128.3, 128.2, 127.4, 120.9, 119.8, 115.2, 114.8, 114.0, 113.9, 72.4, 55.3 ppm; IR(KBr): *ν* = 3285, 3058, 2925, 1655, 1609, 1507, 1368, 1254 cm^−1^; HRMS (ESI-TOF) *m*/*z*: [M + H]^+^ calcd for C_32_H_23_N_2_O_4_H 501.1808, found 501.1812.

#### 2-(4-Benzoyl-5-phenylfuran-2-yl)-2-(4-fluorophenyl)-2,3-dihydroquinazolin-4(1*H*)-one (3d)

Following the general procedure, 100 mg (0.282 mmol, 1.0 equiv.) of 1d, 57 mg (0.423 mmol, 1.5 equiv.) of 2a, 10 mg (10 mol%) of KAuCl_4_ and 91 mg (0.564 mmol, 2.0 equiv.) of FeCl_3_ was used and the reaction time was 12 h. After flash column chromatography on silica gel (eluted with *R*_f_: 0.5; hexane/ethyl acetate mixture 10/3.5), 95 mg of 3d was obtained in 68% yield as a yellow solid. Mp: 138–140 °C; ^1^H NMR (400 MHz, CDCl_3_): *δ* 7.91 (d, *J* = 7.4 Hz, 1H), 7.73–7.47 (m, 7H), 7.39–7.24 (m, 6H), 7.17–7.06 (m, 2H), 6.98–6.87 (m, 1H), 6.80–6.69 (m, 2H), 6.45 (s, 1H), 5.07 (br s, 1H) ppm; ^13^C NMR (100 MHz, CDCl_3_): *δ* 191.0, 163.8, 162.2 (d, ^3^*J*_C–F_ = 250.148 Hz), 156.4, 152.9, 145.1, 137.4, 136.4, 134.4, 133.0, 129.6, 129.4, 129.12 (d, ^2^*J*_C–F_ = 8.069 Hz), 128.9, 128.3, 128.2, 127.4, 120.9, 120.0, 115.7 (d, ^1^*J*_C–F_ = 21.274 Hz), 115.1, 114.9, 114.0, 72.3 ppm; IR(KBr): *ν* = 3283, 3060, 2922, 1656, 1609, 1493, 1367, 1229 cm^−1^; HRMS (ESI-TOF) *m*/*z*: [M + H]^+^ calcd for C_31_H_20_FN_2_O_3_H 489.1609, found 489.1607.

#### 2-(4-(4-Methylbenzoyl)-5-*p*-tolylfuran-2-yl)-2-phenyl-2,3-dihydroquinazolin-4(1*H*)-one (3e)

Following the general procedure, 100 mg (0.274 mmol, 1.0 equiv.) of 1e, 56 mg (0.412 mmol, 1.5 equiv.) of 2a, 10 mg (10 mol%) of KAuCl_4_ and 89 mg (0.549 mmol, 2.0 equiv.) of FeCl_3_ was used and the reaction time was 12 h. After flash column chromatography on silica gel (eluted with *R*_f_: 0.5; hexane/ethyl acetate mixture 10/3.5), 103 mg of 3e was obtained in 75% yield as a yellow solid. Mp: 223–225 °C; ^1^H NMR (500 MHz, CDCl_3_): *δ* 7.90 (d, *J* = 7.4 Hz, 1H), 7.62–7.56 (m, 4H), 7.51 (d, *J* = 8.0 Hz, 2H), 7.44–7.39 (m, 3H), 7.36–7.30 (m, 1H), 7.14 (d, *J* = 7.3 Hz, 2H), 7.06 (d, *J* = 7.1 Hz, 2H), 6.93–6.87 (m, 1H), 6.73 (d, *J* = 7.9 Hz, 1H), 6.69 (s, 1H), 6.55–6.27 (m, 1H), 5.28–5.02 (br s, 1H), 2.37 (s, 3H), 2.30 (s, 3H) ppm; ^13^C NMR (100 MHz, CDCl_3_): *δ* 190.8, 163.8, 156.2, 152.6, 145.3, 143.8, 140.6, 139.4, 135.0, 134.2, 129.7, 129.5, 129.0, 128.9, 128.6, 128.3, 127.2, 126.9, 126.3, 120.4, 119.6, 115.1, 114.8, 114.0, 72.6, 21.5, 21.2 ppm; IR(KBr): *ν* = 3357, 3050, 2918, 1653, 1612, 1507, 1372, 1271, 1179 cm^−1^; HRMS (ESI-TOF) *m*/*z*: [M + H]^+^ calcd for C_33_H_25_N_2_O_3_H 499.2016, found 499.2023.

#### 2-(4-(4-Methylbenzoyl)-5-*p*-tolylfuran-2-yl)-2-*p*-tolyl-2,3-dihydroquinazolin-4(1*H*)-one (3f)

Following the general procedure, 100 mg (0.264 mmol, 1.0 equiv.) of 1f, 54 mg (0.396 mmol, 1.5 equiv.) of 2a, 10 mg (10 mol%) of KAuCl_4_ and 85 mg (0.529 mmol, 2.0 equiv.) of FeCl_3_ was used and the reaction time was 12 h. After flash column chromatography on silica gel (eluted with *R*_f_: 0.5; hexane/ethyl acetate mixture 10/3.5), 100 mg of 3f was obtained in 74% yield as a yellow solid. Mp: 210–212 °C; ^1^H NMR (500 MHz, CDCl_3_): *δ* 7.91 (dd, *J* = 7.4, 1.0 Hz, 1H), 7.60 (d, *J* = 8.0 Hz, 2H), 7.51 (d, *J* = 8.2 Hz, 2H), 7.45 (d, *J* = 8.2 Hz, 2H), 7.35–7.31 (m, 1H), 7.22 (d, *J* = 8.0 Hz, 2H), 7.14 (d, *J* = 8.0 Hz, 2H), 7.07 (d, *J* = 8.2 Hz, 2H), 6.92–6.88 (m, 1H), 6.71 (d, *J* = 8.0 Hz, 1H), 6.68 (s, 1H), 6.27 (s, 1H), 5.06 (br s, 1H), 2.38 (s, 6H), 2.30 (s, 3H) ppm; ^13^C NMR (100 MHz, CDCl_3_): *δ* 190.8, 163.9, 156.1, 152.8, 145.4, 143.7, 139.5, 139.3, 137.7, 135.0, 134.1, 129.7, 129.3, 128.9, 128.8, 128.2, 127.2, 126.8, 126.4, 120.4, 119.5, 115.1, 114.8, 113.9, 72.5, 21.5, 21.2, 21.0 ppm; IR(KBr): *ν* = 3346, 3033, 2918, 1660, 1643, 1503, 1374, 1179 cm^−1^; HRMS (ESI-TOF) *m*/*z*: [M + H]^+^ calcd for C_34_H_27_N_2_O_3_H 513.2172, found 513.2176.

#### 2-(4-Fluorophenyl)-2-(4-(4-methylbenzoyl)-5-*p*-tolylfuran-2-yl)-2,3-dihydroquinazolin-4(1*H*)-one (3g)

Following the general procedure, 100 mg (0.261 mmol, 1.0 equiv.) of 1g, 53 mg (0.392 mmol, 1.5 equiv.) of 2a, 10 mg (10 mol%) of KAuCl_4_ and 85 mg (0.523 mmol, 2.0 equiv.) of FeCl_3_ was used and the reaction time was 12 h. After flash column chromatography on silica gel (eluted with *R*_f_: 0.5; hexane/ethyl acetate mixture 10/3.5), 96 mg of 3g was obtained in 71% yield as a yellow solid. Mp: 223–225 °C; ^1^H NMR (400 MHz, CDCl_3_): *δ* 7.91 (dd, *J* = 7.7, 1.2 Hz, 1H), 7.63–7.55 (m, 4H), 7.50 (d, *J* = 8.1 Hz, 2H), 7.38–7.31 (m, 1H), 7.16–7.06 (m, 6H), 6.94–6.88 (m, 1H), 6.73 (d, *J* = 8.0 Hz, 1H), 6.70 (s, 1H), 6.39 (s, 1H), 5.06 (br s, 1H), 2.38 (s, 3H), 2.31 (s, 3H) ppm; ^13^C NMR (100 MHz, CDCl_3_): *δ* 190.8, 163.8, 163.2 (d, ^3^*J*_C–F_ = 249.4 Hz), 156.3, 152.3, 145.1, 143.9, 139.6, 136.6, 134.9, 134.3, 129.7, 129.1, 129.0 (d, ^2^*J*_C–F_ = 7.3 Hz), 128.3, 127.2, 126.3, 120.5, 119.9, 115.7 (d, ^1^*J*_C–F_ = 22.0 Hz), 115.2, 114.9, 114.1, 72.3, 21.6, 21.3 ppm; IR(KBr): *ν* = 3343, 3051, 2918, 1662, 1643, 1507, 1373, 1234, 893 cm^−1^; HRMS (ESI-TOF) *m*/*z*: [M + H]^+^ calcd for C_33_H_24_FN_2_O_3_H 517.1922, found 517.1931.

#### 2-(4-Benzoyl-5-phenylfuran-2-yl)-2-(*m*-tolyl)-2,3-dihydroquinazolin-4(1*H*)-one (3h)

Following the general procedure, 100 mg (0.285 mmol, 1.0 equiv.) of 1h, 58 mg (0.427 mmol, 1.5 equiv.) of 2a, 10 mg (10 mol%) of KAuCl_4_ and 92 mg (0.571 mmol, 2.0 equiv.) of FeCl_3_ was used and the reaction time was 12 h. After flash column chromatography on silica gel (eluted with *R*_f_: 0.6; hexane/ethyl acetate mixture 10/3.0), 52 mg of 3h was obtained in 37% yield as a light yellow solid. Mp: 224–226 °C; ^1^H NMR (400 MHz, CDCl_3_): *δ* 7.93 (dd, *J* = 7.8, 1.4 Hz, 1H), 7.71–7.67 (m, 2H), 7.63–7.57 (m, 2H), 7.53–7.46 (m, 2H), 7.37–7.23 (m, 9H), 6.94–6.89 (m, 1H), 6.76–6.72 (m, 2H), 6.33 (s, 1H), 5.08 (br s, 1H), 2.39 (s, 3H). ppm; ^13^C NMR (100 MHz, DMSO-d_6_ & CDCl_3_): *δ* 189.1, 162.5, 153.7, 153.0, 145.2, 140.2, 136.0, 132.0, 131.4, 127.9, 127.8, 127.7, 127.6, 126.8, 126.7, 126.5, 126.2, 125.8, 122.7, 119.2, 116.4, 113.4, 113.2, 111.6, 70.6, 19.9. ppm; IR(KBr): *ν* = 3335, 3061, 2919, 1668, 1487, 1265, 1148, 756 cm^−1^; HRMS (ESI-TOF) *m*/*z*: [M + H]^+^ calcd for C_32_H_24_N_2_O_3_H 485.1865, found 485.1864.

#### 2-(4-(4-Methylbenzoyl)-5-(*p*-tolyl)furan-2-yl)-2-(*m*-tolyl)-2,3-dihydroquinazolin-4(1*H*)-one (3i)

Following the general procedure, 100 mg (0.264 mmol, 1.0 equiv.) of 1i, 54 mg (0.396 mmol, 1.5 equiv.) of 2a, 10 mg (10 mol%) of KAuCl_4_ and 85 mg (0.529 mmol, 2.0 equiv.) of FeCl_3_ was used and the reaction time was 12 h. After flash column chromatography on silica gel (eluted with *R*_f_: 0.6; hexane/ethyl acetate mixture 10/3.0), 56 mg of 3i was obtained in 41% yield as a light yellow solid. Mp: 204–206 °C; ^1^H NMR (400 MHz, CDCl_3_): *δ* 7.93 (dd, *J* = 7.8, 1.3 Hz, 1H), 7.60 (d, *J* = 8.1 Hz, 2H), 7.52 (d, *J* = 8.1 Hz, 2H), 7.46 (s, 1H), 7.37–7.23 (m, 4H), 7.15 (d, *J* = 7.9 Hz, 2H), 7.08 (d, *J* = 8.0 Hz, 2H), 6.94–6.89 (m, 1H), 6.72 (d, *J* = 7.9 Hz, 1H), 6.68 (s, 1H), 6.24 (s, 1H), 5.04 (br s, 1H), 2.38 (d, *J* = 1.5 Hz, 6H), 2.31 (s, 3H). ppm; ^13^C NMR (75 MHz, CDCl_3_): *δ* 190.9, 163.8, 156.2, 152.7, 145.3, 143.8, 140.4, 139.5, 138.7, 135.1, 134.3, 130.5, 129.8, 129.0, 128.9, 128.6, 128.4, 127.6, 127.3, 126.4, 123.9, 120.5, 119.8, 115.2, 114.9, 114.1, 72.7, 21.6, 21.5, 21.3. ppm; IR(KBr): *ν* = 3448, 3034, 2919, 1663, 1498, 1271, 1155, 754 cm^−1^; HRMS (ESI-TOF) *m*/*z*: [M + H]^+^ calcd for C_34_H_28_N_2_O_3_H 513.2178, found 513.2180.

#### 2-(4-(3-Methylbenzoyl)-5-(*m*-tolyl)furan-2-yl)-2-phenyl-2,3-dihydroquinazolin-4(1*H*)-one (3j)

Following the general procedure, 100 mg (0.274 mmol, 1.0 equiv.) of 1j, 56 mg (0.412 mmol, 1.5 equiv.) of 2a, 10 mg (10 mol%) of KAuCl_4_ and 89 mg (0.549 mmol, 2.0 equiv.) of FeCl_3_ was used and the reaction time was 12 h. After flash column chromatography on silica gel (eluted with *R*_f_: 0.6; hexane/ethyl acetate mixture 10/3.0), 54 mg of 3j was obtained in 39% yield as a light yellow solid. Mp: 102–104 °C; ^1^H NMR (400 MHz, CDCl_3_): *δ* 7.93 (dd, *J* = 7.7, 1.3 Hz, 1H), 7.61–7.56 (m, 2H), 7.51–7.42 (m, 5H), 7.40–7.28 (m, 4H), 7.21 (t, *J* = 7.6 Hz, 1H), 7.17–7.12 (m, 1H), 7.09 (d, *J* = 7.6 Hz, 1H), 6.94–6.89 (m, 1H), 6.76–6.72 (m, 2H), 6.48 (s, 1H), 5.09 (br s, 1H), 2.29 (s, 3H), 2.25 (s, 3H). ppm; ^13^C NMR (75 MHz, CDCl_3_): *δ* 191.3, 164.0, 156.6, 152.9, 145.2, 140.4, 138.1, 137.9, 137.6, 134.4, 133.7, 130.1, 129.7, 129.0, 128.8, 128.4, 128.2, 128.1, 126.9, 126.8, 124.7, 121.0, 119.9, 115.1, 114.9, 114.0, 72.7, 21.2, 21.1. ppm; IR(KBr): *ν* = 3318, 3056, 2920, 1657, 1485, 1270, 1144, 754 cm^−1^; HRMS (ESI-TOF) *m*/*z*: [M + H]^+^ calcd for C_33_H_26_N_2_O_3_H 499.2022, found 499.2027.

#### 2-(4-Benzoyl-5-phenylfuran-2-yl)-2-hexyl-2,3-dihydroquinazolin-4(1*H*)-one (3k)

Following the general procedure, 100 mg (0.29 mmol, 1.0 equiv.) of 1k, 59 mg (0.436 mmol, 1.5 equiv.) of 2a, 11 mg (10 mol%) of KAuCl_4_ and 94 mg (0.581 mmol, 2.0 equiv.) of FeCl_3_ was used and the reaction time was 12 h. After flash column chromatography on silica gel (eluted with *R*_f_: 0.6; hexane/ethyl acetate mixture 10/3.0), 60 mg of 3k was obtained in 43% yield as a light yellow semi solid. ^1^H NMR (400 MHz, CDCl_3_): *δ* 7.87 (dd, *J* = 7.8, 1.3 Hz, 1H), 7.68–7.65 (m, 2H), 7.60–7.57 (m, 2H), 7.49–7.44 (m, 1H), 7.33–7.24 (m, 6H), 7.20 (s, 1H), 6.88–6.83 (m, 1H), 6.68 (d, *J* = 7.9 Hz, 1H), 6.52 (s, 1H), 4.75 (br s, 1H), 2.26–2.13 (m, 2H), 1.42–1.23 (m, 8H), 0.91–0.85 (m, 3H). ppm; ^13^C NMR (100 MHz, CDCl_3_): *δ* 191.2, 165.0, 155.6, 154.7, 145.6, 137.5, 134.2, 132.9, 129.6, 129.2, 128.2, 127.3, 126.1, 121.0, 119.4, 114.8, 114.6, 111.1, 70.2, 39.9, 31.5, 29.0, 23.1, 22.4, 13.9. ppm; IR(KBr): *ν* = 3301, 3060, 2927, 1661, 1486, 1270, 1150, 756 cm^−1^; HRMS (ESI-TOF) *m*/*z*: [M + H]^+^ calcd for C_31_H_30_N_2_O_3_H 479.2335, found 479.2339.

#### 2-(4-Benzoyl-5-phenylfuran-2-yl)-5-methyl-2-phenyl-2,3-dihydroquinazolin-4(1*H*)-one (3l)

Following the general procedure, 100 mg (0.3 mmol, 1.0 equiv.) of 1a, 67 mg (0.45 mmol, 1.5 equiv.) of 2b, 11 mg (10 mol%) of KAuCl_4_ and 97 mg (0.6 mmol, 2.0 equiv.) of FeCl_3_ was used and the reaction time was 12 h. After flash column chromatography on silica gel (eluted with *R*_f_: 0.7; hexane/ethyl acetate mixture 10/2.5), 104 mg of 3l was obtained in 72% yield as a yellow solid. Mp: 228–230 °C; ^1^H NMR (400 MHz, CDCl_3_): *δ* 7.72–7.68 (m, 2H), 7.64–7.57 (m, 3H), 7.55–7.48 (m, 1H), 7.46–7.42 (m, 3H), 7.39–7.33 (m, 2H), 7.30–7.25 (m, 4H), 7.17 (t, *J* = 7.8 Hz, 1H), 6.75 (s, 1H), 6.71 (d, *J* = 7.5 Hz, 1H), 6.59 (d, *J* = 7.9 Hz, 1H), 6.21 (s, 1H), 5.03 (d, *J* = 1.4 Hz, 1H), 2.68 (s, 3H) ppm; ^13^C NMR (100 MHz, CDCl_3_): *δ* 191.1, 164.3, 156.2, 153.2, 146.5, 142.3, 140.4, 137.6, 133.1, 133.0, 129.7, 129.6, 129.3, 129.1, 128.8, 128.3, 128.2, 127.4, 127.0, 123.6, 120.9, 114.2, 113.8, 113.2, 72.1, 22.2 ppm; IR(KBr): *ν* = 3341, 3060, 2927, 1659, 1636, 1519, 1368, 1265 cm^−1^; HRMS (ESI-TOF) *m*/*z*: [M + H]^+^ calcd for C_32_H_23_N_2_O_3_H 485.1859, found 485.1873.

#### 2-(4-Benzoyl-5-phenylfuran-2-yl)-6-chloro-2-phenyl-2,3-dihydroquinazolin-4(1*H*)-one (3m)

Following the general procedure, 100 mg (0.3 mmol, 1.0 equiv.) of 1a, 76 mg (0.45 mmol, 1.5 equiv.) of 2c, 11 mg (10 mol%) of KAuCl_4_ and 97 mg (0.6 mmol, 2.0 equiv.) of FeCl_3_ was used and the reaction time was 12 h. After flash column chromatography on silica gel (eluted with *R*_f_: 0.6; hexane/ethyl acetate mixture 10/3.0), 98 mg of 3m was obtained in 65% yield as a yellow solid. Mp: 148–150 °C; ^1^H NMR (400 MHz, CDCl_3_): *δ* 7.89 (s, 1H), 7.69 (d, *J* = 7.4 Hz, 2H), 7.61–7.41 (m, 8H), 7.38–7.33 (m, 1H), 7.31–7.23 (m, 5H), 6.75–6.67 (m, 2H), 6.48 (s, 1H), 5.16 (s, 1H), ppm; ^13^C NMR (75 MHz, DMSO-d_6_ & CDCl_3_): *δ* 190.6, 162.7, 155.6, 153.1, 144.5, 140.5, 137.1, 133.3, 132.6, 129.1, 128.9, 128.8, 128.7, 128.1, 127.9, 127.7, 127.1, 126.9, 126.7, 123.0, 120.5, 116.2, 115.5, 113.4, 72.1 ppm; IR(KBr): *ν* = 3283, 3060, 2924, 1658, 1610, 1486, 1265, 891 cm^−1^; HRMS (ESI-TOF) *m*/*z*: [M + H]^+^ calcd for C_31_H_20_ClN_2_O_3_H 505.1313, found 505.1322.

#### 2-(4-Benzoyl-5-phenylfuran-2-yl)-6-bromo-2-phenyl-2,3-dihydroquinazolin-4(1*H*)-one (3n)

Following the general procedure, 100 mg (0.3 mmol, 1.0 equiv.) of 1a, 86 mg (0.45 mmol, 1.5 equiv.) of 2d, 11 mg (10 mol%) of KAuCl_4_ and 97 mg (0.6 mmol, 2.0 equiv.) of FeCl_3_ was used and the reaction time was 12 h. After flash column chromatography on silica gel (eluted with *R*_f_: 0.6; hexane/ethyl acetate mixture 10/3.0), 103 mg of 3n was obtained in 63% yield as a yellow solid. Mp: 186–188 °C; ^1^H NMR (400 MHz, CDCl_3_): *δ* 8.02 (d, *J* = 2.0 Hz, 1H), 7.73–7.67 (m, 2H), 7.61–7.48 (m, 5H), 7.47–7.40 (m, 4H), 7.38–7.33 (m, 1H), 7.30–7.22 (m, 4H), 6.73 (s, 1H), 6.64 (d, *J* = 8.5 Hz, 1H), 6.49 (s, 1H), 5.18 (br s, 1H) ppm; ^13^C NMR (100 MHz, CDCl_3_): *δ* 191.0, 162.7, 156.4, 152.7, 144.2, 140.0, 137.4, 136.9, 133.1, 130.8, 129.8, 129.6, 129.4, 128.9, 128.8, 128.3, 128.2, 127.4, 126.9, 120.9, 116.7, 114.1, 111.8, 72.7 ppm; IR(KBr): *ν* = 3325, 3064, 2923, 1651, 1605, 1490, 1319, 1144 cm^−1^; HRMS (ESI-TOF) *m*/*z*: [M + H]^+^ calcd for C_31_H_20_BrN_2_O_3_H 549.0808, found 549.0824.

#### 2-(4-Benzoyl-5-phenylfuran-2-yl)-6-iodo-2-phenyl-2,3-dihydroquinazolin-4(1*H*)-one (3o)

Following the general procedure, 100 mg (0.3 mmol, 1.0 equiv.) of 1a, 117 mg (0.45 mmol, 1.5 equiv.) of 2e, 11 mg (10 mol%) of KAuCl_4_ and 96 mg (0.6 mmol, 2.0 equiv.) of FeCl_3_ was used and the reaction time was 12 h. After flash column chromatography on silica gel (eluted with *R*_f_: 0.6; hexane/ethyl acetate mixture 10/3.0), 107 mg of 3o was obtained in 60% yield as a yellow solid. Mp: 200–202 °C; ^1^H NMR (400 MHz, CDCl_3_): *δ* 8.21 (s, 1H), 7.70 (d, *J* = 7.4 Hz, 2H), 7.62–7.23 (m, 14H), 6.73 (s, 1H), 6.53 (d, *J* = 8.3 Hz, 1H), 6.41 (s, 1H), 5.16 (br s, 1H) ppm; ^13^C NMR (75 MHz, DMSO-d_6_ & CDCl_3_): *δ* 189.4, 161.5, 154.3, 152.7, 144.9, 140.3, 140.2, 136.3, 134.5, 131.7, 128.1, 128.0, 127.7, 127.1, 127.0, 126.9, 126.2, 125.9, 119.5, 116.2, 115.7, 112.2, 70.9 ppm; IR(KBr): *ν* = 3324, 3062, 1646, 1602, 1489, 1318, 1146, 890 cm^−1^; HRMS (ESI-TOF) *m*/*z*: [M + H]^+^ calcd for C_31_H_20_IN_2_O_3_H 597.0669, found 597.0696.

#### 2-(4-Benzoyl-5-phenylfuran-2-yl)-5-methyl-2-(*p*-tolyl)-2,3-dihydroquinazolin-4(1*H*)-one (3p)

Following the general procedure, 100 mg (0.285 mmol, 1.0 equiv.) of 1b, 64 mg (0.428 mmol, 1.5 equiv.) of 2b, 10 mg (10 mol%) of KAuCl_4_ and 92 mg (0.571 mmol, 2.0 equiv.) of FeCl_3_ was used and the reaction time was 12 h. After flash column chromatography on silica gel (eluted with *R*_f_: 0.7; hexane/ethyl acetate mixture 10/2.5), 103 mg of 3p was obtained in 72% yield as a yellow solid. Mp: 202–204 °C; ^1^H NMR (400 MHz, CDCl_3_): *δ* 7.69 (d, *J* = 7.3 Hz, 2H), 7.66–7.60 (m, 2H), 7.54–7.43 (m, 3H), 7.38–7.32 (m, 2H), 7.29–7.21 (m, 5H), 7.18–7.13 (m, 1H), 6.73 (s, 1H), 6.69 (d, *J* = 7.4 Hz, 1H), 6.58 (d, *J* = 7.9 Hz, 1H), 6.29 (s, 1H), 5.03 (br s, 1H), 2.67 (s, 3H), 2.38 (s, 3H) ppm; ^13^C NMR (100 MHz, CDCl_3_): *δ* 191.1, 164.4, 156.1, 153.4, 146.6, 142.2, 139.6, 137.67, 137.61, 133.0, 132.9, 129.6, 129.4, 129.2, 129.1, 128.3, 128.2, 127.4, 126.8, 123.5, 120.8, 114.0, 113.7, 113.2, 71.9, 22.1, 21.1 ppm; IR(KBr): *ν* = 3321, 3037, 2919, 1652, 1601, 1507, 1373, 1269 cm^−1^; HRMS (ESI-TOF) *m*/*z*: [M + H]^+^ calcd for C_33_H_25_N_2_O_3_H 499.2016, found 499.2021.

#### 2-(4-Benzoyl-5-phenylfuran-2-yl)-2-(4-methoxyphenyl)-5-methyl-2,3-dihydroquinazolin-4(1*H*)-one (3q)

Following the general procedure, 100 mg (0.273 mmol, 1.0 equiv.) of 1c, 61 mg (0.409 mmol, 1.5 equiv.) of 2b, 10 mg (10 mol%) of KAuCl_4_ and 88 mg (0.546 mmol, 2.0 equiv.) of FeCl_3_ was used and the reaction time was 12 h. After flash column chromatography on silica gel (eluted with *R*_f_: 0.7; hexane/ethyl acetate mixture 10/2.5), 98 mg of 3q was obtained in 70% yield as a yellow solid. Mp: 223–225 °C; ^1^H NMR (400 MHz, CDCl_3_): *δ* 7.69 (d, *J* = 7.3 Hz, 2H), 7.66–7.61 (m, 2H), 7.54–7.46 (m, 3H), 7.39–7.33 (m, 2H), 7.30–7.25 (m, 3H), 7.19–7.13 (m, 1H), 6.92 (d, *J* = 8.8 Hz, 2H), 6.73 (s, 1H), 6.69 (d, *J* = 7.4 Hz, 1H), 6.58 (d, *J* = 7.8 Hz, 1H), 6.25 (s, 1H), 5.01 (br s, 1H), 3.83 (s, 3H), 2.67 (s, 3H) ppm; ^13^C NMR (100 MHz, CDCl_3_): *δ* 191.1, 164.4, 160.4, 156.1, 153.4, 146.6, 142.2, 137.6, 133.1, 132.9, 132.5, 129.6, 129.3, 129.1, 128.4, 128.3, 128.2, 127.4, 123.6, 120.8, 114.1, 113.9, 113.7, 113.2, 71.8, 55.3, 22.2 ppm; IR(KBr): *ν* = 3326, 3034, 2920, 1652, 1601, 1506, 1371, 1251 cm^−1^; HRMS (ESI-TOF) *m*/*z*: [M + H]^+^ calcd for C_33_H_25_N_2_O_4_H 515.1965, found 515.1974.

#### 2-(4-Benzoyl-5-phenylfuran-2-yl)-2-(4-fluorophenyl)-5-methyl-2,3-dihydroquinazolin-4(1*H*)-one (3r)

Following the general procedure, 100 mg (0.282 mmol, 1.0 equiv.) of 1d, 63 mg (0.423 mmol, 1.5 equiv.) of 2b, 10 mg (10 mol%) of KAuCl_4_ and 91 mg (0.564 mmol, 2.0 equiv.) of FeCl_3_ was used and the reaction time was 12 h. After flash column chromatography on silica gel (eluted with *R*_f_: 0.7; hexane/ethyl acetate mixture 10/2.5), 98 mg of 3r was obtained in 69% yield as a yellow solid. Mp: 170–172 °C; ^1^H NMR (400 MHz, CDCl_3_): *δ* 7.68 (d, *J* = 7.4 Hz, 2H), 7.63–7.49 (m, 4H), 7.38–7.25 (m, 6H), 7.21–7.07 (m, 3H), 6.77–6.69 (m, 2H), 6.59 (d, *J* = 7.9 Hz, 1H), 6.38 (s, 1H), 5.02 (br s, 1H), 2.66 (s, 3H) ppm; ^13^C NMR (100 MHz, CDCl_3_): 191.0, 164.3, 163.2 (d, ^3^*J*_C–F_ = 250.1 Hz), 156.3, 153.0, 146.4, 142.3, 137.5, 136.4, 133.2, 133.0, 129.6, 129.4, 129.17 (d, ^2^*J*_C–F_ = 8.8 Hz), 129.0, 128.36, 128.30, 127.4, 123.8, 120.9, 115.7 (d, ^1^*J*_C–F_ = 21.2 Hz), 114.2, 113.7, 113.2, 72.3, 22.1 ppm; IR(KBr): *ν* = 3397, 3062, 2923, 1661, 1601, 1504, 1360, 1224 cm^−1^; HRMS (ESI-TOF) *m*/*z*: [M + H]^+^ calcd for C_32_H_22_FN_2_O_3_H 503.1765, found 503.1766.

#### 2-(4-Benzoyl-5-phenylfuran-2-yl)-6-chloro-2-(4-fluorophenyl)-2,3-dihydroquinazolin-4(1*H*)-one (3s)

Following the general procedure, 100 mg (0.282 mmol, 1.0 equiv.) of 1d, 72 mg (0.423 mmol, 1.5 equiv.) of 2c, 10 mg (10 mol%) of KAuCl_4_ and 91 mg (0.564 mmol, 2.0 equiv.) of FeCl_3_ was used and the reaction time was 12 h. After flash column chromatography on silica gel (eluted with *R*_f_: 0.6; hexane/ethyl acetate mixture 10/3.0), 93 mg of 3s was obtained in 63% yield as a yellow solid. Mp: 130–132 °C; ^1^H NMR (400 MHz, CDCl_3_): *δ* 7.85 (d, *J* = 2.2 Hz, 1H), 7.68 (d, *J* = 7.4 Hz, 2H), 7.59–7.47 (m, 5H), 7.37–7.22 (m, 6H), 7.15–7.06 (m, 2H), 6.76–6.67 (m, 3H), 5.19 (br s, 1H) ppm; ^13^C NMR (100 MHz, CDCl_3_): *δ* 190.9, 163.3 (d, ^3^*J*_C–F_ = 250.8 Hz), 162.8, 156.5, 152.4, 143.5, 137.3, 136.0, 134.3, 133.1, 129.6, 129.5, 129.13 (d, ^2^*J*_C–F_ = 8.1 Hz), 128.8, 128.4, 128.3, 127.9, 127.5, 125.2, 120.9, 116.5, 116.4, 115.8 (d, ^1^*J*_C–F_ = 22.0 Hz), 114.2, 72.3 ppm; IR(KBr): *ν* = 3279, 3062, 2923, 1660, 1607, 1486, 1347, 1231 cm^−1^; HRMS (ESI-TOF) *m*/*z*: [M + H]^+^ calcd for C_31_H_19_ClFN_2_O_3_H 523.1219, found 523.1230.

#### 2-(4-Benzoyl-5-phenylfuran-2-yl)-6-bromo-2-(4-fluorophenyl)-2,3-dihydroquinazolin-4(1*H*)-one (3t)

Following the general procedure, 100 mg (0.282 mmol, 1.0 equiv.) of 1d, 91 mg (0.423 mmol, 1.5 equiv.) of 2d, 10 mg (10 mol%) of KAuCl_4_ and 91 mg (0.564 mmol, 2.0 equiv.) of FeCl_3_ was used and the reaction time was 12 h. After flash column chromatography on silica gel (eluted with *R*_f_: 0.6; hexane/ethyl acetate mixture 10/3.0), 98 mg of 3t was obtained in 61% yield as a yellow solid. Mp: 198–200 °C; ^1^H NMR (400 MHz, CDCl_3_): *δ* 8.02 (d, *J* = 2.0 Hz, 1H), 7.72–7.66 (m, 2H), 7.59–7.49 (m, 4H), 7.46–7.41 (m, 1H), 7.38–7.23 (m, 7H), 7.15–7.09 (m, 1H), 6.74 (s, 1H), 6.65 (d, *J* = 8.5 Hz, 1H), 6.56 (s, 1H), 5.13 (br s, 1H) ppm; ^13^C NMR (100 MHz, CDCl_3_): *δ* 190.9, 163.3 (d, ^3^*J*_C–F_ = 250.1 Hz), 162.7, 156.5, 152.4, 144.0, 137.3, 137.0, 135.9, 133.1, 130.9, 129.6, 129.5, 129.12 (d, ^2^*J*_C–F_ = 8.8 Hz), 128.8, 128.4, 128.3, 127.5, 120.9, 116.820, 116.820, 115.8 (d, ^1^*J*_C–F_ = 22.0 Hz), 112.1, 72.3 ppm; IR(KBr): *ν* = 3319, 3064, 1654, 1604, 1499, 1319, 1230, 1144 cm^−1^; HRMS (ESI-TOF) *m*/*z*: [M + H]^+^ calcd for C_31_H_19_BrFN_2_O_3_H 567.0714, found, 567.0732.

#### 2-(4-Benzoyl-5-phenylfuran-2-yl)-2-(4-fluorophenyl)-6-iodo-2,3-dihydroquinazolin-4(1*H*)-one (3u)

Following the general procedure, 100 mg (0.282 mmol, 1.0 equiv.) of 1d, 111 mg (0.423 mmol, 1.5 equiv.) of 2e, 10 mg (10 mol%) of KAuCl_4_ and 91 mg (0.564 mmol, 2.0 equiv.) of FeCl_3_ was used and the reaction time was 12 h. After flash column chromatography on silica gel (eluted with *R*_f_: 0.6; hexane/ethyl acetate mixture 10/3.0), 107 mg of 3u was obtained in 62% yield as a yellow solid. Mp: 194–196 °C; ^1^H NMR (400 MHz, CDCl_3_): *δ* 8.20 (d, *J* = 1.5 Hz, 1H), 7.72–7.67 (m, 2H), 7.62–7.49 (m, 6H), 7.40–7.33 (m, 2H), 7.30–7.24 (m, 4H), 7.16–7.08 (m, 1H), 6.74 (s, 1H), 6.54 (d, *J* = 8.4 Hz, 1H), 6.49 (s, 1H), 5.13 (br s, 1H) ppm; ^13^C NMR (75 MHz, DMSO-d_6_ & CDCl_3_): *δ* 188.8, 160.95, 160.93 (d, ^3^*J*_C–F_ = 247.0 Hz), 153.8, 152.1, 144.4, 140.0, 136.0, 135.8, 134.0, 131.4, 127.7, 127.6, 127.5, 126.7 (d, ^2^*J*_C–F_ = 11.0 Hz), 125.8, 119.1, 115.9, 115.3, 113.4 (d, ^1^*J*_C–F_ = 22.0 Hz), 111.7, 70.0 ppm; IR(KBr): *ν* = 3319, 3062, 1650, 1601, 1494, 1319, 1231, 1153 cm^−1^; HRMS (ESI-TOF) *m*/*z*: [M + H]^+^ calcd for C_31_H_19_FIN_2_O_3_H 615.0575, found, 615.0602.

#### 5-Methyl-2-(4-(4-methylbenzoyl)-5-*p*-tolylfuran-2-yl)-2-phenyl-2,3-dihydroquinazolin-4(1*H*)-one (3v)

Following the general procedure, 100 mg (0.274 mmol, 1.0 equiv.) of 1e, 62 mg (0.412 mmol, 1.5 equiv.) of 2b, 10 mg (10 mol%) of KAuCl_4_ and 89 mg (0.549 mmol, 2.0 equiv.) of FeCl_3_ was used and the reaction time was 12 h. After flash column chromatography on silica gel (eluted with *R*_f_: 0.7; hexane/ethyl acetate mixture 10/2.5), 99 mg of 3v was obtained in 70% yield as a yellow solid. Mp: 180–182 °C; ^1^H NMR (400 MHz, CDCl_3_): *δ* 7.64–7.52 (m, 6H), 7.46–7.40 (m, 3H), 7.19–7.13 (m, 3H), 7.08 (d, *J* = 8.0 Hz, 2H), 6.73–6.66 (m, 2H), 6.58 (d, *J* = 7.9 Hz, 1H), 6.24 (s, 1H), 5.04 (br s, 1H), 2.68 (s, 3H), 2.39 (s, 3H), 2.31 (s, 3H) ppm; ^13^C NMR (100 MHz, CDCl_3_): *δ* 190.8, 164.3, 156.2, 152.6, 146.6, 143.8, 142.3, 140.6, 139.4, 135.2, 133.0, 129.7, 129.6, 129.0, 128.9, 128.7, 127.2, 127.0, 126.5, 123.6, 120.4, 114.3, 113.7, 113.2, 72.1, 22.2, 21.6, 21.3 ppm; IR(KBr): *ν* = 3326, 3056, 2922, 1652, 1602, 1499, 1368, 1269 cm^−1^; HRMS (ESI-TOF) *m*/*z*: [M + H]^+^ calcd for C_34_H_27_N_2_O_3_H 513.2172, found 513.2177.

#### 5-Methyl-2-(4-(4-methylbenzoyl)-5-*p*-tolylfuran-2-yl)-2-*p*-tolyl-2,3-dihydroquinazolin-4(1*H*)-one (3w)

Following the general procedure, 100 mg (0.264 mmol, 1.0 equiv.) of 1f, 60 mg (0.396 mmol, 1.5 equiv.) of 2b, 10 mg (10 mol%) of KAuCl_4_ and 85 mg (0.529 mmol, 2.0 equiv.) of FeCl_3_ was used and the reaction time was 12 h. After flash column chromatography on silica gel (eluted with *R*_f_: 0.7; hexane/ethyl acetate mixture 10/2.5), 95 mg of 3w was obtained in 68% yield as a yellow solid. Mp: 228–230 °C; ^1^H NMR (400 MHz, CDCl_3_): *δ* 7.60 (d, *J* = 8.1 Hz, 2H), 7.54 (d, *J* = 8.3 Hz, 2H), 7.44 (d, *J* = 8.3 Hz, 2H), 7.21 (d, *J* = 7.9 Hz, 2H), 7.19–7.12 (m, 3H), 7.08 (d, *J* = 8.0 Hz, 2H), 6.71–6.67 (m, 2H), 6.56 (d, *J* = 7.9 Hz, 1H), 6.24–6.17 (m, 1H), 5.04–4.98 (br s, 1H), 2.68 (s, 3H), 2.38 (d, *J* = 4.8 Hz, 6H), 2.31 (s, 3H) ppm; ^13^C NMR (100 MHz, CDCl_3_): *δ* 190.9, 164.4, 156.1, 152.8, 146.7, 143.7, 142.2, 139.6, 139.3, 137.7, 135.2, 133.0, 129.7, 129.3, 129.0, 128.9, 127.2, 126.9, 126.5, 123.5, 120.4, 114.1, 113.7, 113.2, 71.9, 22.1, 21.6, 21.3, 21.0 ppm; IR(KBr): *ν* = 3328, 3040, 2922, 1648, 1602, 1501, 1368, 1269, 891 cm^−1^; HRMS (ESI-TOF) *m*/*z*: [M + H]^+^ calcd for C_35_H_29_N_2_O_3_H 527.2329, found 527.2346.

#### 2-(4-Benzoyl-5-phenylfuran-2-yl)-2,7-diphenyl-5-(trifluoromethyl)-2,3-dihydropyrido[2,3-*d*]pyrimidin-4(1*H*)-one (3x)

Following the general procedure, 100 mg (0.3 mmol, 1.0 equiv.) of 1a, 112 mg (0.45 mmol, 1.5 equiv.) of 2f, 10 mg (10 mol%) of KAuCl_4_ and 97 mg (0.6 mmol, 2.0 equiv.) of FeCl_3_ was used and the reaction time was 12 h. After flash column chromatography on silica gel (eluted with *R*_f_: 0.6; hexane/ethyl acetate mixture 10/3.0), 106 mg of 3x was obtained in 58% yield as a yellow solid. Mp: 140–142 °C; ^1^H NMR (500 MHz, CDCl_3_): *δ*, 8.03–7.96 (m, 2H), 7.79–7.73 (m, 2H), 7.64–7.57 (m, 4H), 7.54–7.45 (m, 7H), 7.40–7.35 (m, 2H), 7.29–7.25 (m, 5H), 6.83 (s, 1H), 6.39 (s, 1H) ppm; ^13^C NMR (100 MHz, CDCl_3_): *δ* 190.9, 161.5, 160.7, 157.7, 156.7, 152.2, 139.7 (q, ^2^*J*_C–F_ = 33.7 Hz), 139.3, 137.4, 136.8, 133.1, 130.7, 129.8, 129.5, 129.4, 128.9, 128.8, 128.3, 128.2, 127.4, 127.2, 126.6, 122.3 (q, ^1^*J*_C–F_ = 272.1 Hz), 120.9, 114.3, 110.78, 110.73, 104.4, 71.2 ppm; IR(KBr): *ν* = 3196, 3063, 2922, 1677, 1579, 1447, 1263, 1156 cm^−1^; HRMS (ESI-TOF) *m*/*z*: [M + H]^+^ calcd for C_37_H_23_F_3_N_3_O_3_H 616.1842, found 616.1869.

#### Dimethyl(2-methyl-5-(4-oxo-2-phenyl-1,2,3,4-tetrahydroquinazolin-2-yl)furan-3-yl)phosphonate (3y)

Following the general procedure, 100 mg (0.359 mmol, 1.0 equiv.) of 1l, 73 mg (0.539 mmol, 1.5 equiv.) of 2a, 14 mg (10 mol%) of KAuCl_4_ and 116 mg (0.719 mmol, 2.0 equiv.) of FeCl_3_ was used and the reaction time was 12 h. After flash column chromatography on silica gel (eluted with *R*_f_: 0.8; hexane/ethyl acetate mixture 10/7.0), 101 mg of 3y was obtained in 68% yield as a light yellow solid. Mp: 226–228 °C; ^1^H NMR (300 MHz, CDCl_3_): *δ* 7.88 (d, *J* = 7.7 Hz, 1H), 7.50–7.30 (m, 6H), 6.87 (t, *J* = 7.7 Hz, 1H), 6.71 (d, *J* = 7.9 Hz, 1H), 6.50 (d, *J* = 3.3 Hz, 1H), 6.27 (s, 1H), 4.99 (br s, 1H), 3.75–3.63 (m, 6H), 2.45 (d, *J* = 1.9 Hz, 3H). ppm; ^13^C NMR (75 MHz, DMSO-d_6_ & CDCl_3_): *δ* 162.2, 158.8, 158.4, 153.0 (d, ^2^*J*_C,P_ = 14.8 Hz), 144.9, 140.4, 131.9, 127.0, 126.5, 125.7, 125.5, 116.1, 113.2, 112.9, 110.2(d, ^2^*J*_C,P_ = 11.5 Hz), 106.0, 103.2, 70.2, 50.6 (d, ^2^*J*_C,P_ = 4.9 Hz), 11.9. ppm; ^31^P NMR (162 MHz, CDCl_3_ & DMSO-d_6_) 21.287 (m); IR(KBr): *ν* = 3443, 3252, 2953, 1659, 1521, 1242, 1018, 830 cm^−1^; HRMS (ESI-TOF) *m*/*z*: [M + H]^+^ calcd for C_21_H_21_N_2_O_5_PH 413.1266, found 413.1266.

#### *N*′-((4-Benzoyl-5-phenylfuran-2-yl)(phenyl)methylene)-2-(prop-2-yn-1-yloxy)benzohydrazide (5)

Following the general procedure, 100 mg (0.297 mmol, 1.0 equiv.) of 1a, 85 mg (0.446 mmol, 1.5 equiv.) of 7, 10 mg (10 mol%) of KAuCl_4_ and 96 mg (0.595 mmol, 2.0 equiv.) of FeCl_3_ was used and the reaction time was 12 h. After flash column chromatography on silica gel (eluted with *R*_f_: 0.6; hexane/ethyl acetate mixture 10/3.0), 97 mg of 8 was obtained in 62% yield as a yellow solid. Mp: 73–75 °C; ^1^H NMR (400 MHz, CDCl_3_): *δ* 10.81 (s, 1H), 8.34 (dd, *J* = 7.8 Hz, 1.7 Hz, 1H), 7.83–7.78 (m, 2H), 7.75–7.70 (m, 2H), 7.69–7.62 (m, 3H), 7.55–7.41 (m, 4H), 7.39–7.33 (m, 2H), 7.30–7.26 (m, 3H), 7.18–7.11 (m, 1H), 7.01 (d, *J* = 8.3 Hz, 1H), 6.72 (s, 1H), 4.18 (d, *J* = 2.2 Hz, 2H), 2.50 (t, *J* = 2.2 Hz, 1H), ppm; ^13^C NMR (100 MHz, CDCl_3_): *δ* 191.4, 161.2, 157.1, 154.9, 149.8, 145.0, 137.4, 133.2, 133.0, 131.4, 130.2, 129.69, 129.62, 129.4, 128.9, 128.6, 128.3, 128.2, 127.9, 122.5, 122.4, 120.8, 116.9, 112.7, 55.8. ppm; IR(KBr): *ν* = 3357, 3297, 3200, 3058, 2923, 2853, 1655, 1598, 1476, 1231, 1012, 752 cm^−1^; HRMS (ESI-TOF) *m*/*z*: [M + H]^+^ calcd for C_34_H_24_N_2_O_4_H 525.1814, found 525.1808.

#### Crystal data 5

C_70_H_53_N_4_O_9_ (*M* =1066.17 g mol^−1^): triclinic, space group *P*1̄ (no. 2), *a* = 9.1182(6) Å, *b* = 13.8291(10) Å, *c* = 23.4126(16) Å, *α* = 103.581(1)°, *β* = 91.205(2)°, *γ* = 98.758(2)°, *V* = 2831.3(3) Å^3^, *Z* = 2, *T* = 294.15 K, *μ*(Mo Kα) = 0.084 mm^−1^, *D*_calc_ = 1.2505 g cm^−3^, 35 814 reflections measured (1.8° ≤ 2*Θ* ≤ 50°), 9961 unique (*R*_int_ = 0.0944, *R*_sigma_ = 0.1499) which were used in all calculations. The final *R*_1_ was 0.1271 (*I* > 2*σ*(*I*)) and w*R*_2_ was 0.3437 (all data). CCDC 1898773.

## Conflicts of interest

There are no conflicts to declare.

## Supplementary Material

RA-010-D0RA06537D-s001

RA-010-D0RA06537D-s002
