# Supplementary material for: Gold(iii) promoted formation of dihydroquinazolinones: double X–H activation by gold
Source: RSC Adv. 2020 Sep 28;10(59):35681–91. doi: 10.1039/d0ra06537d (PMC9056963; doi:10.1039/d0ra06537d)
Supplement: RA-010-D0RA06537D-s001 [file RA-010-D0RA06537D-s001.pdf]

Electronic Supplementary Material (ESI) for RSC Advances.
This journal is © The Royal Society of Chemistry 2020

Supporting Information for

Gold(III)- promoted formation of dihydroquinazolinones: gold double X-H activation†

Veerabhushanam Kadiyala,[a,b] Perla Bharath Kumar,[a,b] Komalla sunil,[a,b] Chittala Emmaniel Raju,[a,b] Balasubramanian Sridhar[c] and Galla V. Karunakar[a,b]*

[a] Fluoro and Agrochemicals Department, CSIR-Indian Institute of Chemical Technology, Hyderabad, 500007, India.
Email: gallavk@iict.res.in

[b] Academy of Scientific and Innovative Research, Ghaziabad- 201002, India

c Center for X-ray Crystallography, CSIR-Indian Institute of Chemical Technology, Hyderabad, 500007, India.

Contents:

1. Copies of ^{1}H and ^{13}C NMR spectra (**3a-3y** and **5**) S2
2. X-ray crystallography data of **3a** and **5** S29

Electronic Supplementary Material (ESI) for Chemical Communications

1. Copies of ^{1}H and ^{13}C NMR spectra (3a-3y and 5).

2-(4-Benzoyl-5-phenylfuran-2-yl)-2-phenyl-2,3-dihydroquinazolin-4(*1H*)-one:**3a**

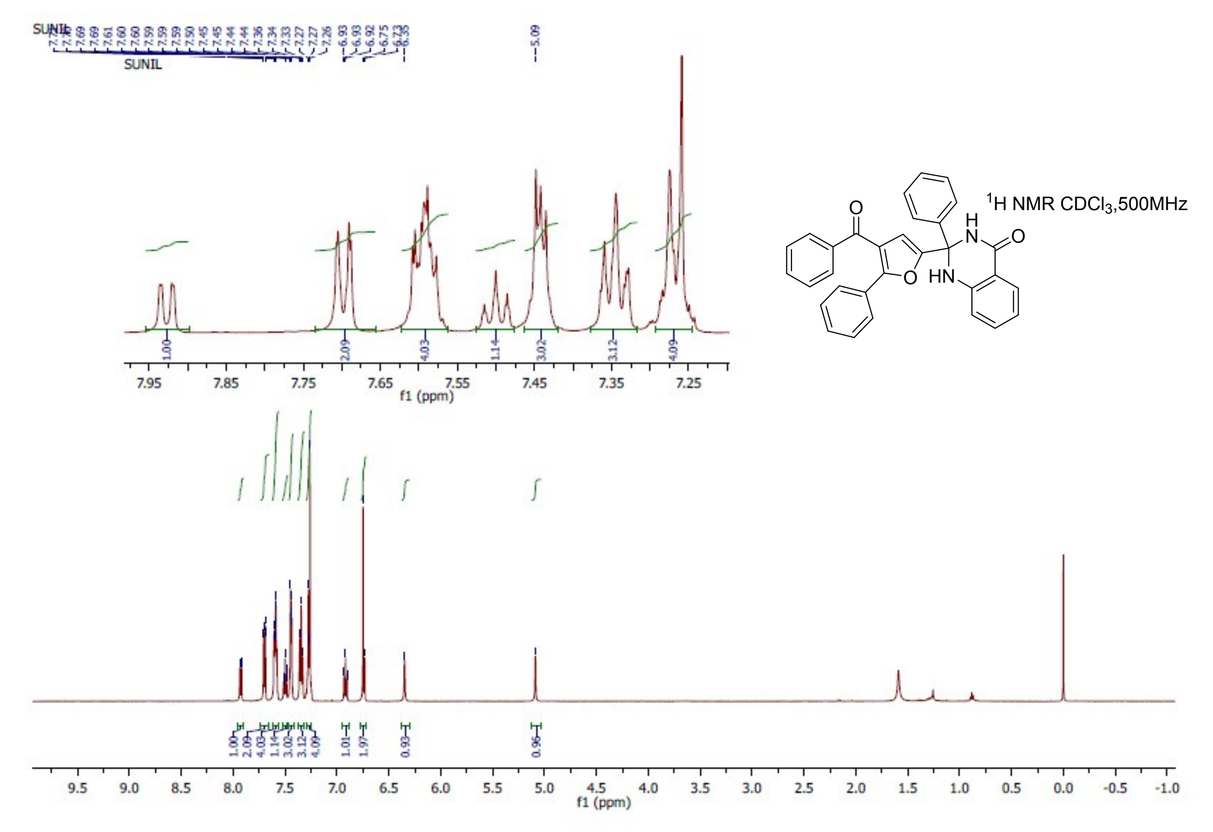

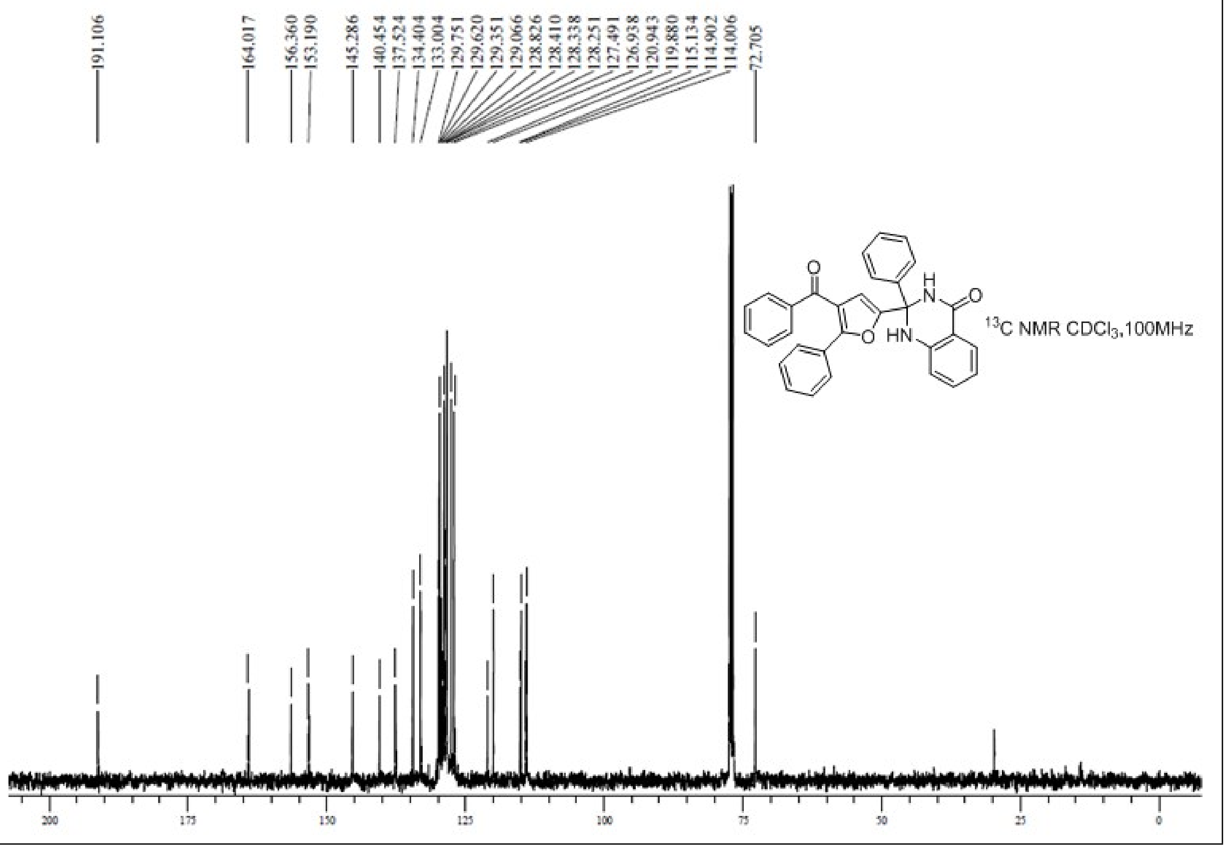

2-(4-Benzoyl-5-phenylfuran-2-yl)-2-(*p*-tolyl)-2,3-dihydroquinazolin-4(*1H*)-one:**3b**

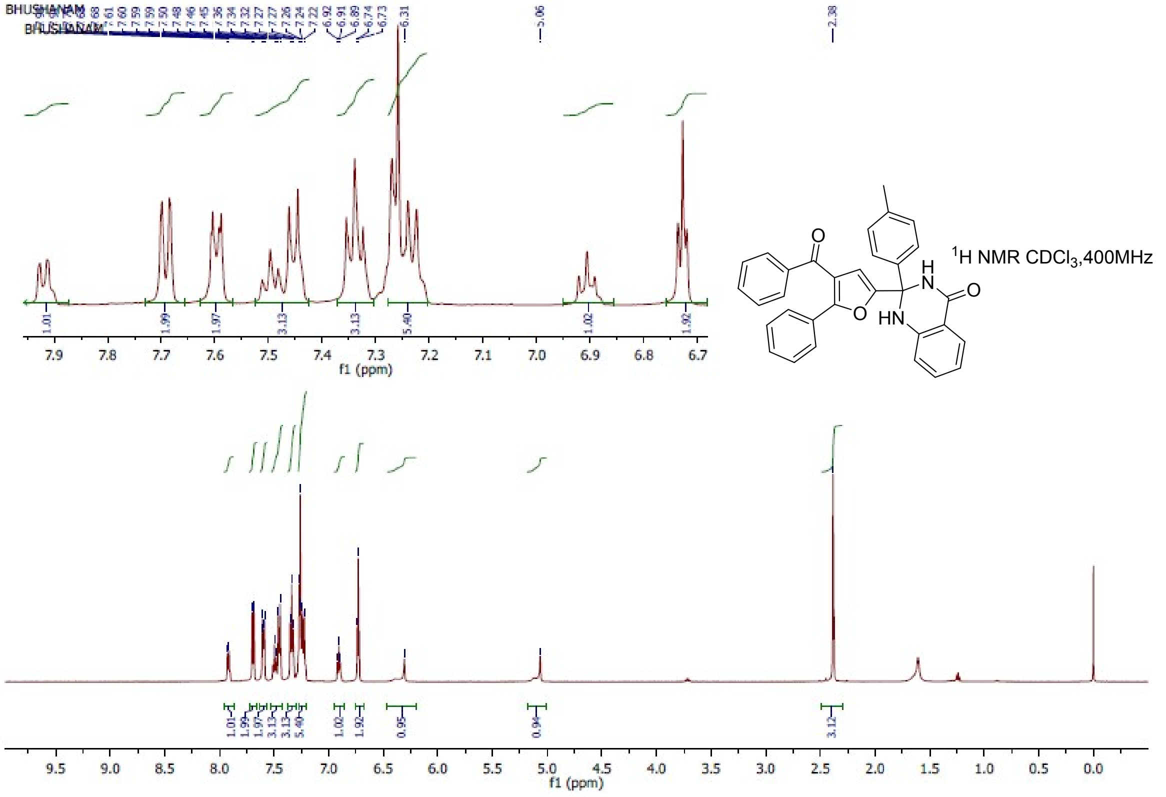

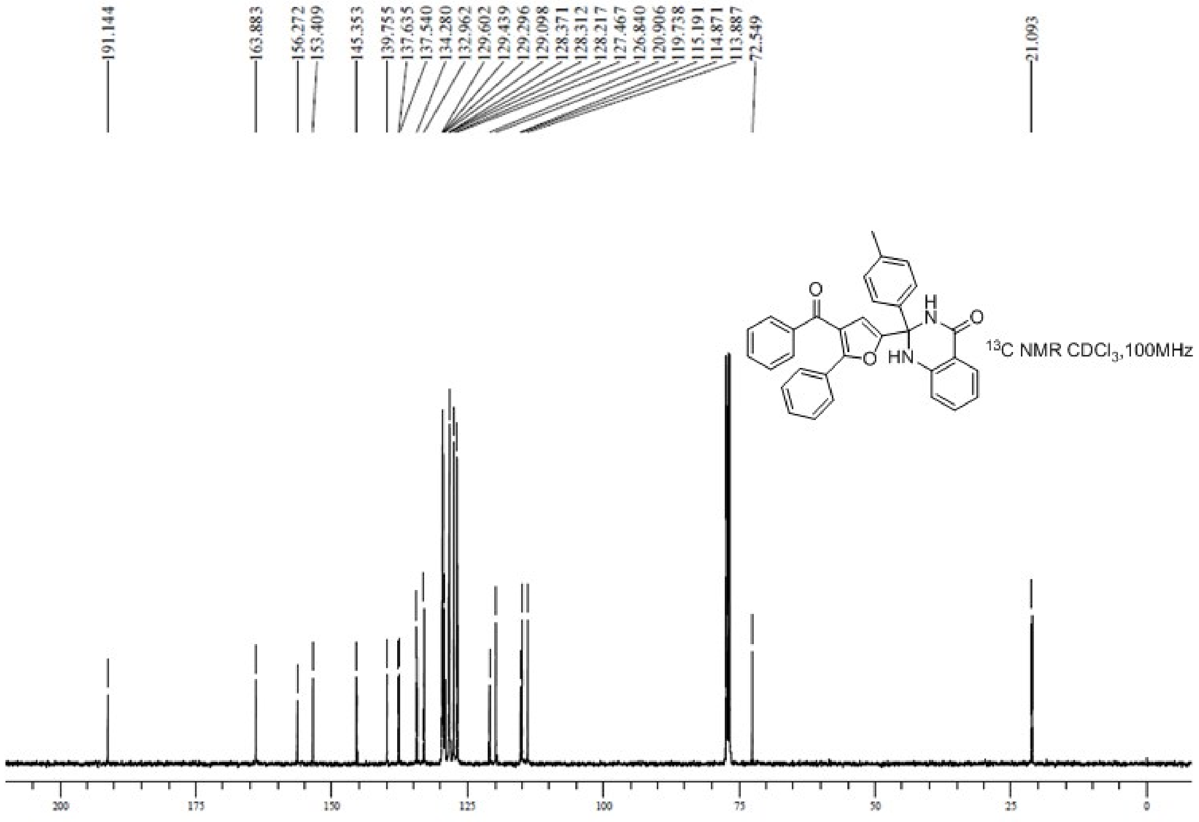

2-(4-Benzoyl-5-phenylfuran-2-yl)-2-(4-methoxyphenyl)-2,3-dihydroquinazolin-4(*1H*)-one:**3c**

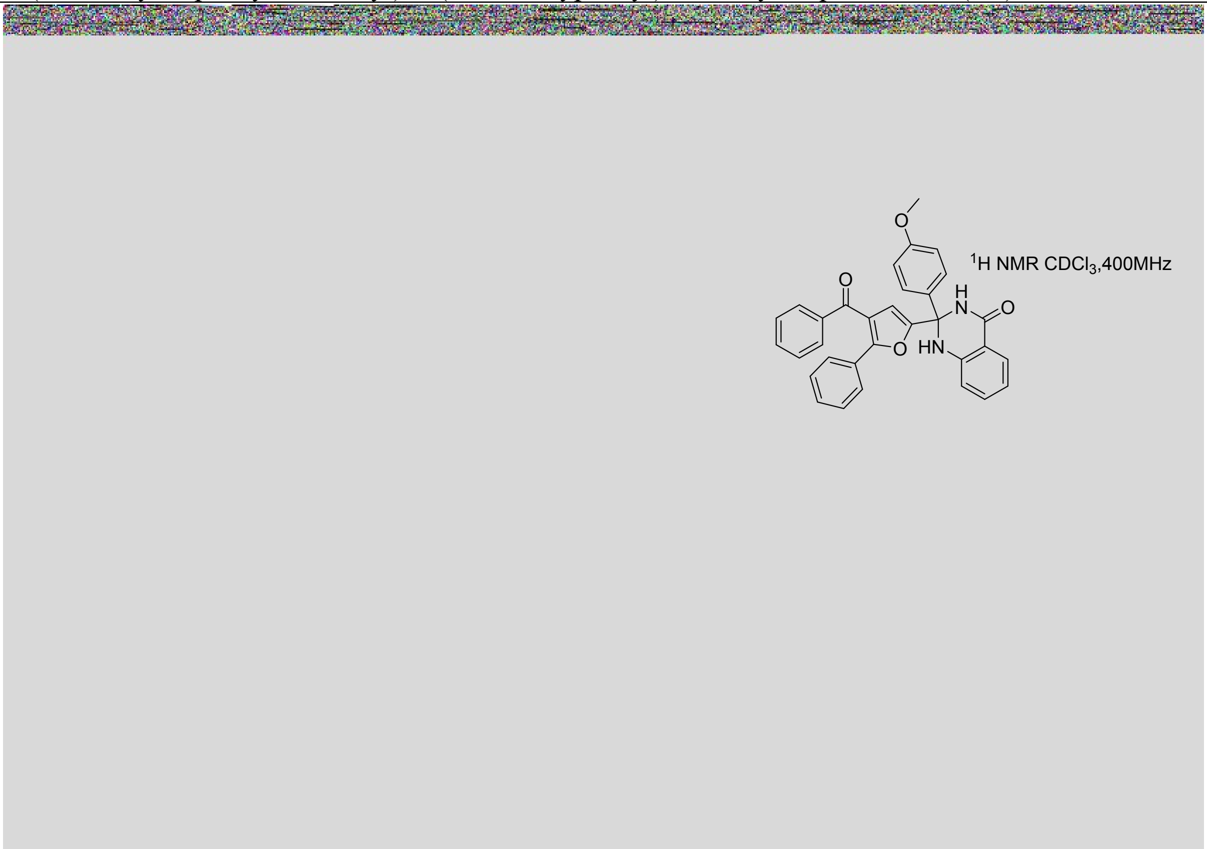

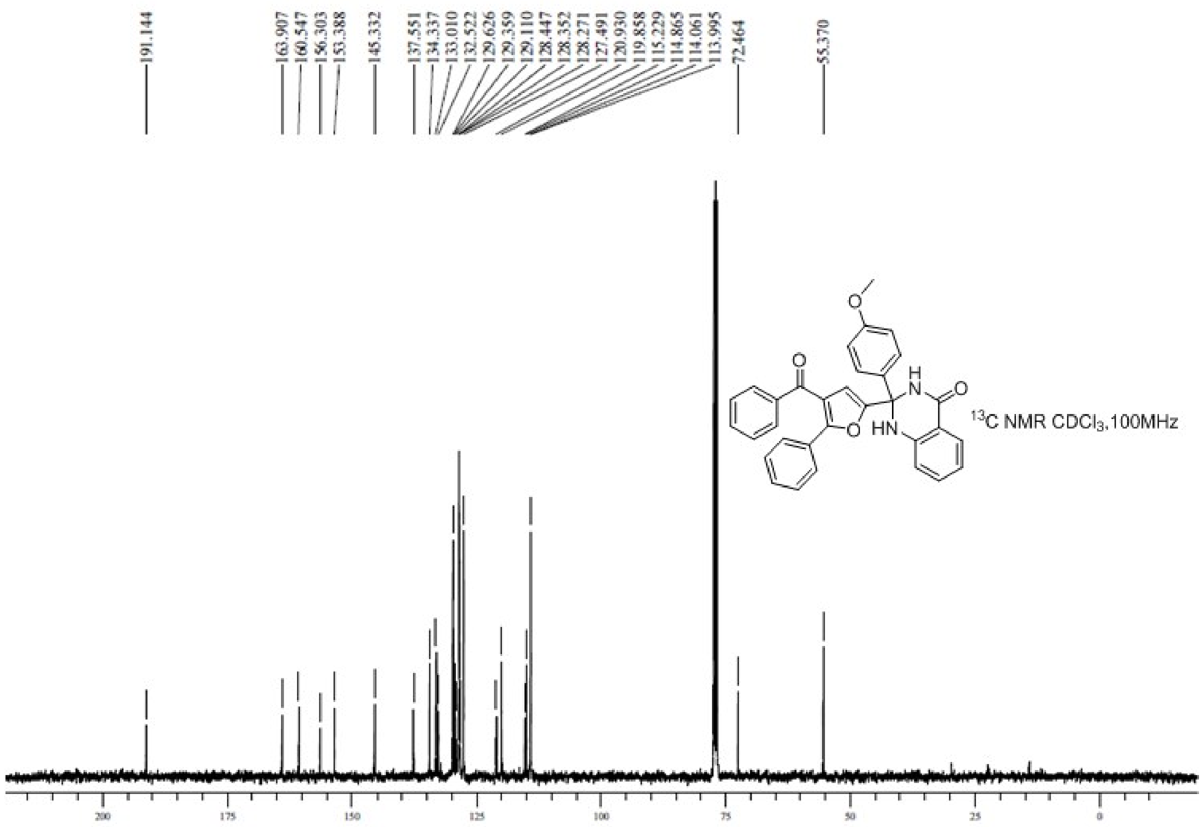

2-(4-Benzoyl-5-phenylfuran-2-yl)-2-(4-fluorophenyl)-2,3-dihydroquinazolin-4(*1H*)-one:**3d**

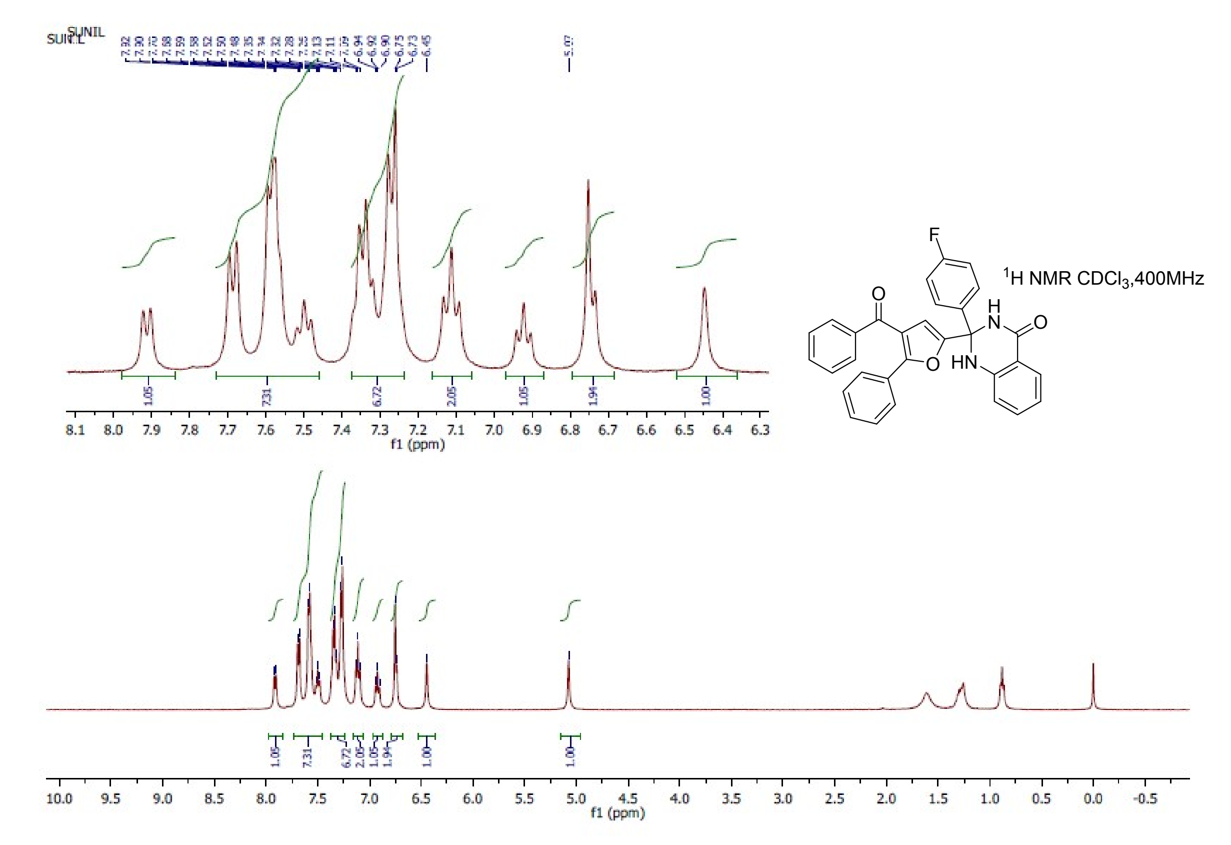

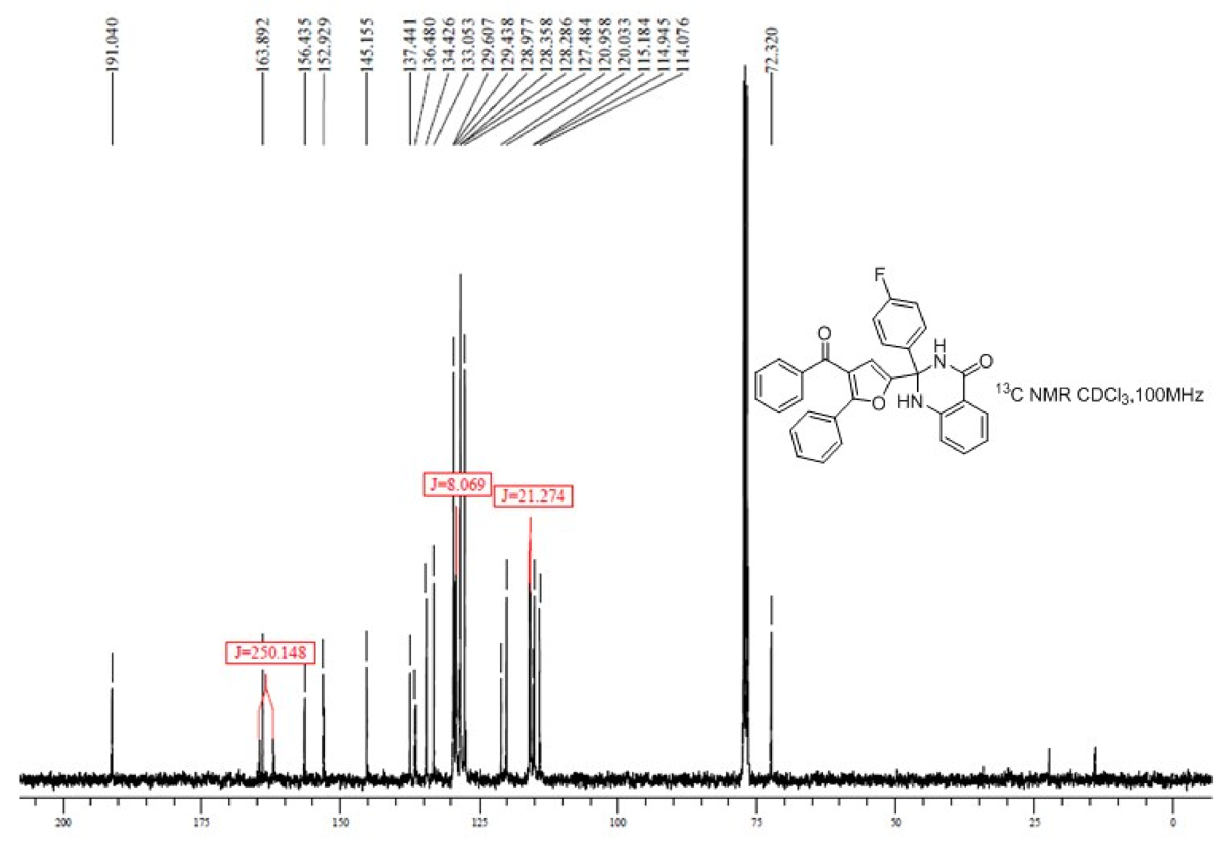

2-(4-(4-Methylbenzoyl)-5-(*p*-tolyl)furan-2-yl)-2-phenyl-2,3-dihydroquinazolin-4(*1H*)-one:**3e**

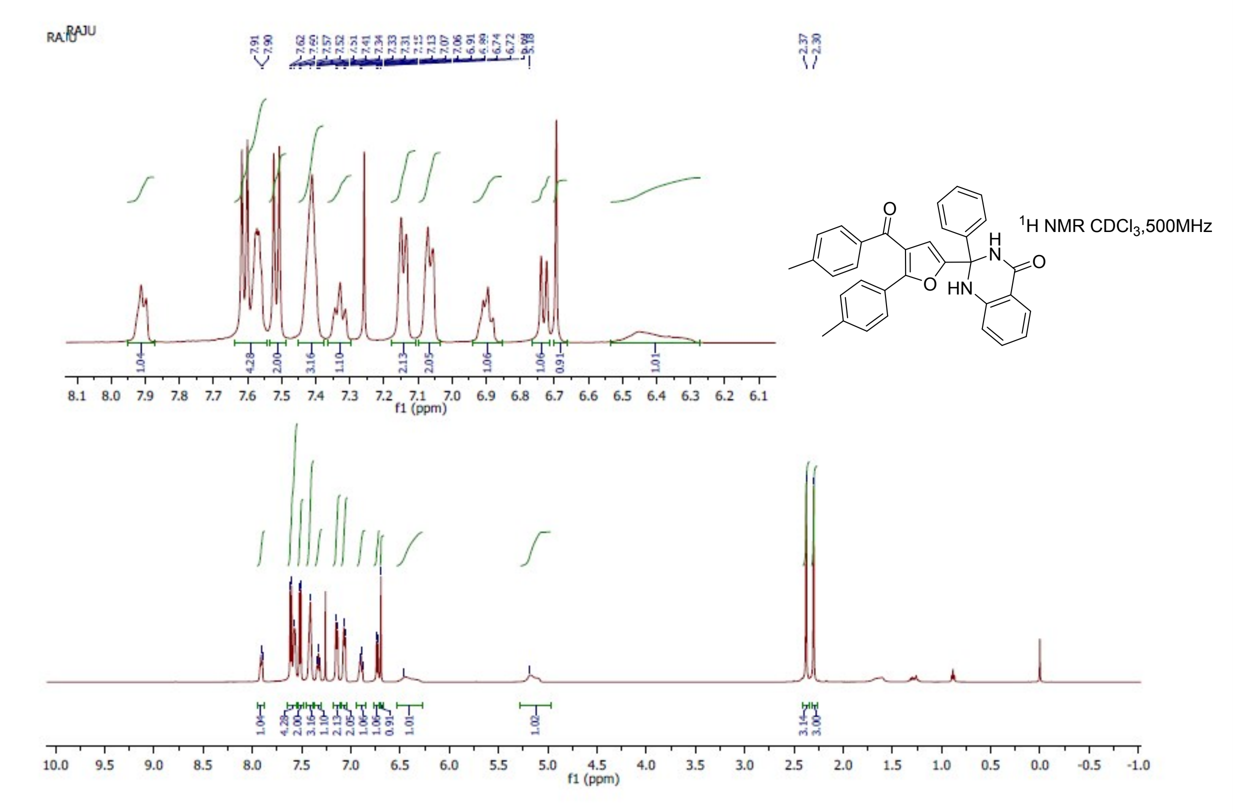

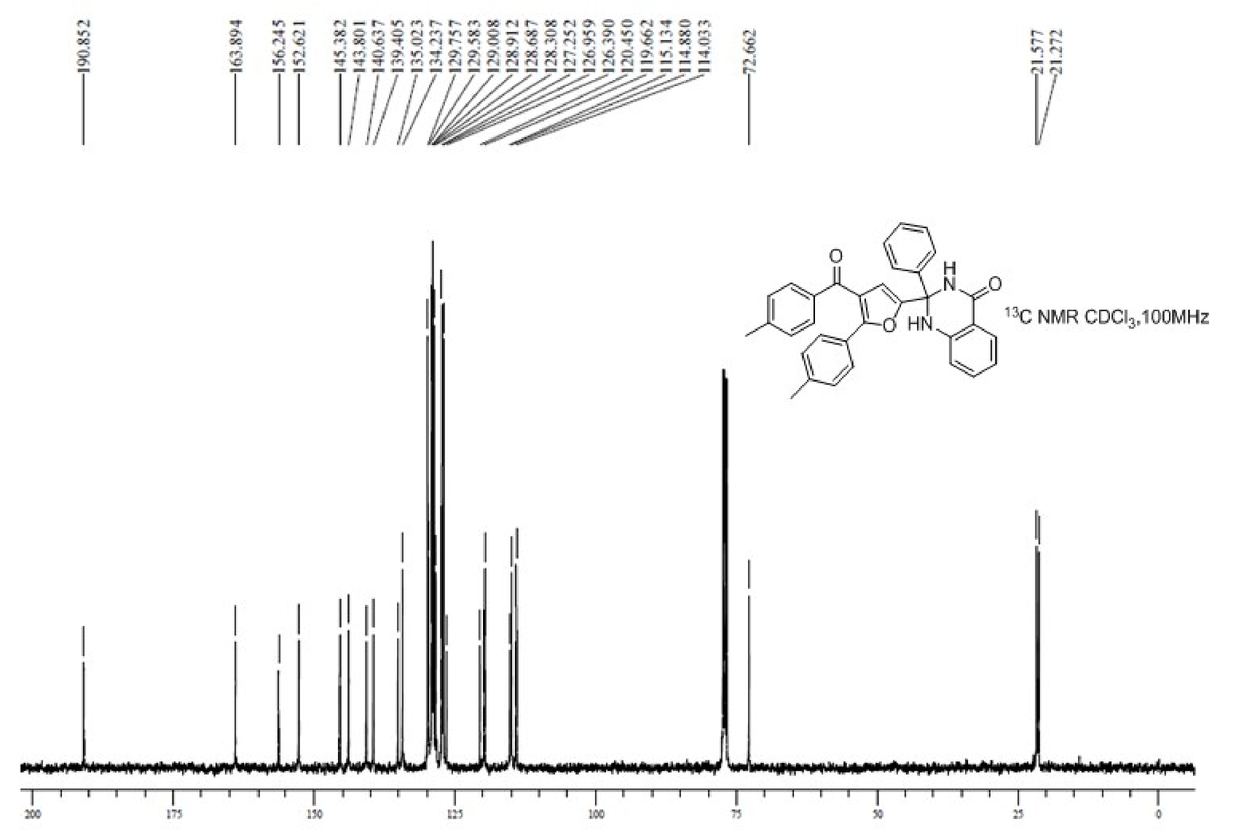

2-(4-(4-Methylbenzoyl)-5-(*p*-tolyl)furan-2-yl)-2-(*p*-tolyl)-2,3-dihydroquinazolin-4(*1H*)-one:**3f**

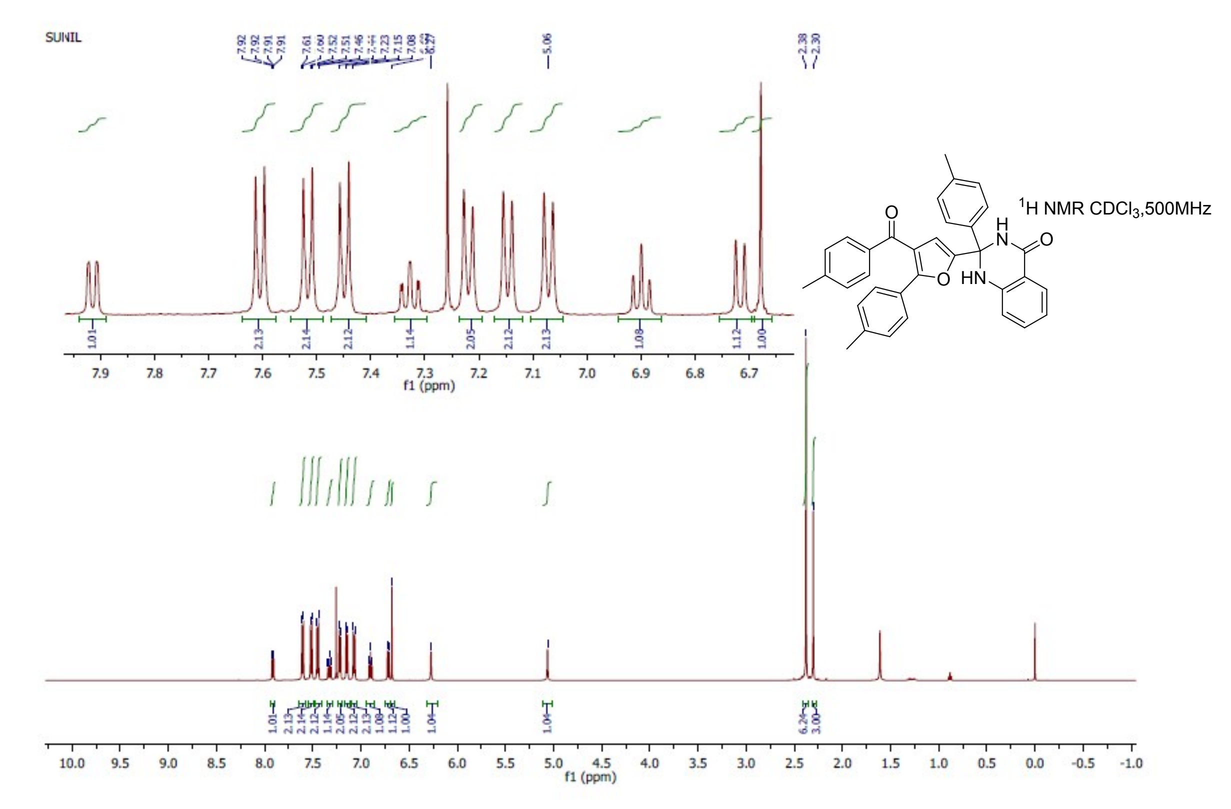

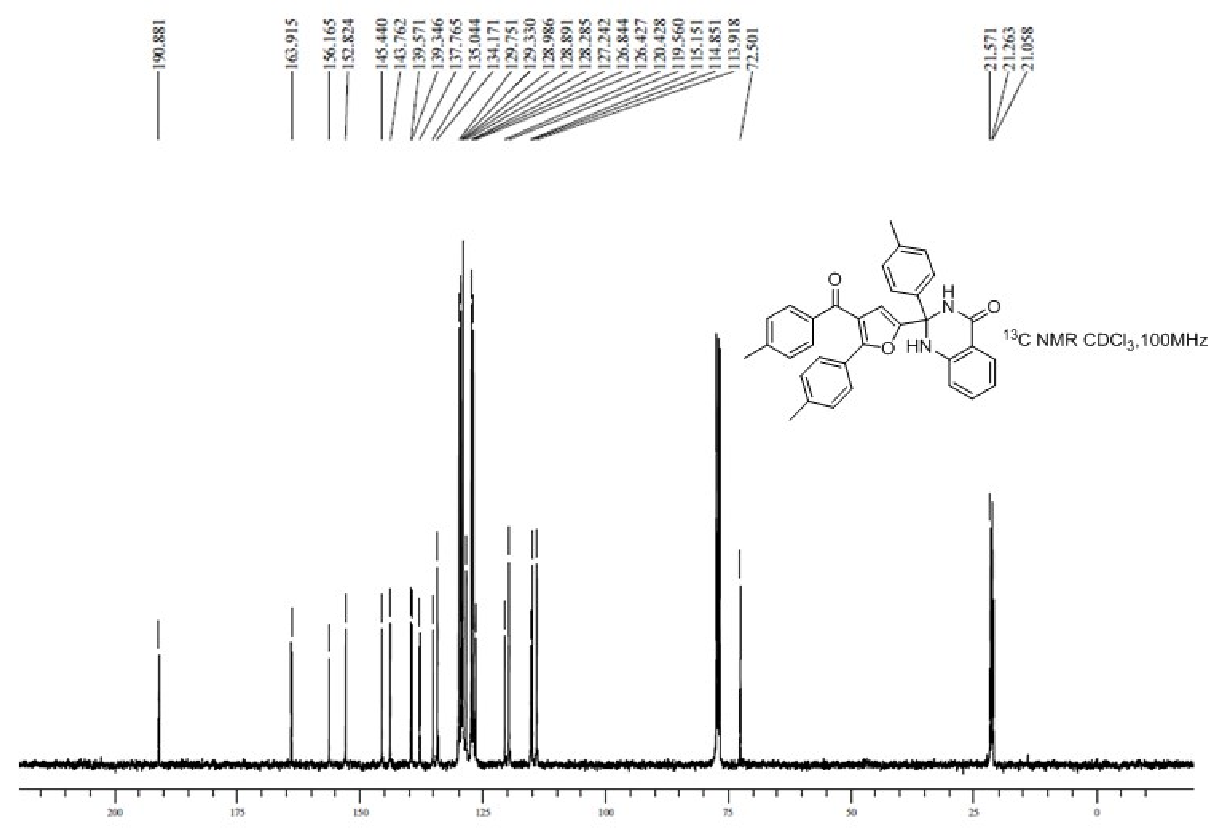

2-(4-Fluorophenyl)-2-(4-(4-methylbenzoyl)-5-(*p*-tolyl)furan-2-yl)-2,3-dihydroquinazolin-4(*1H*)-one:**3g**

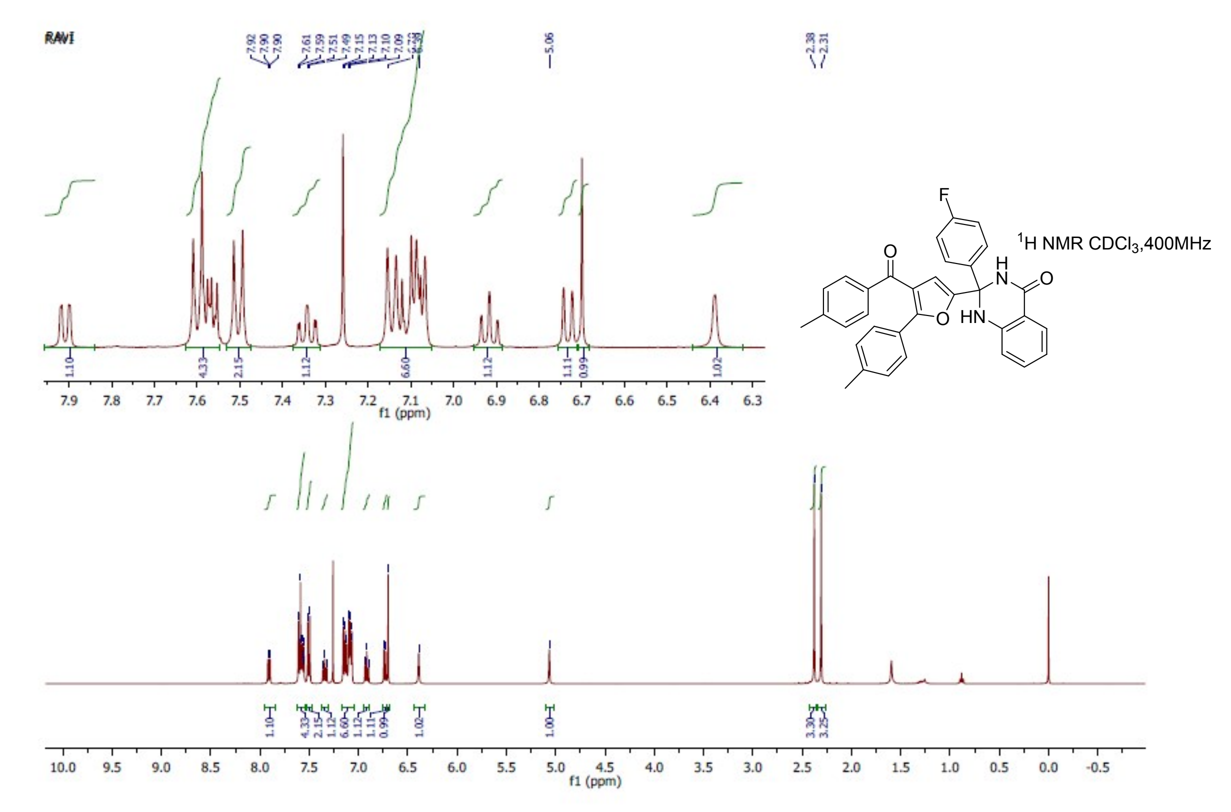

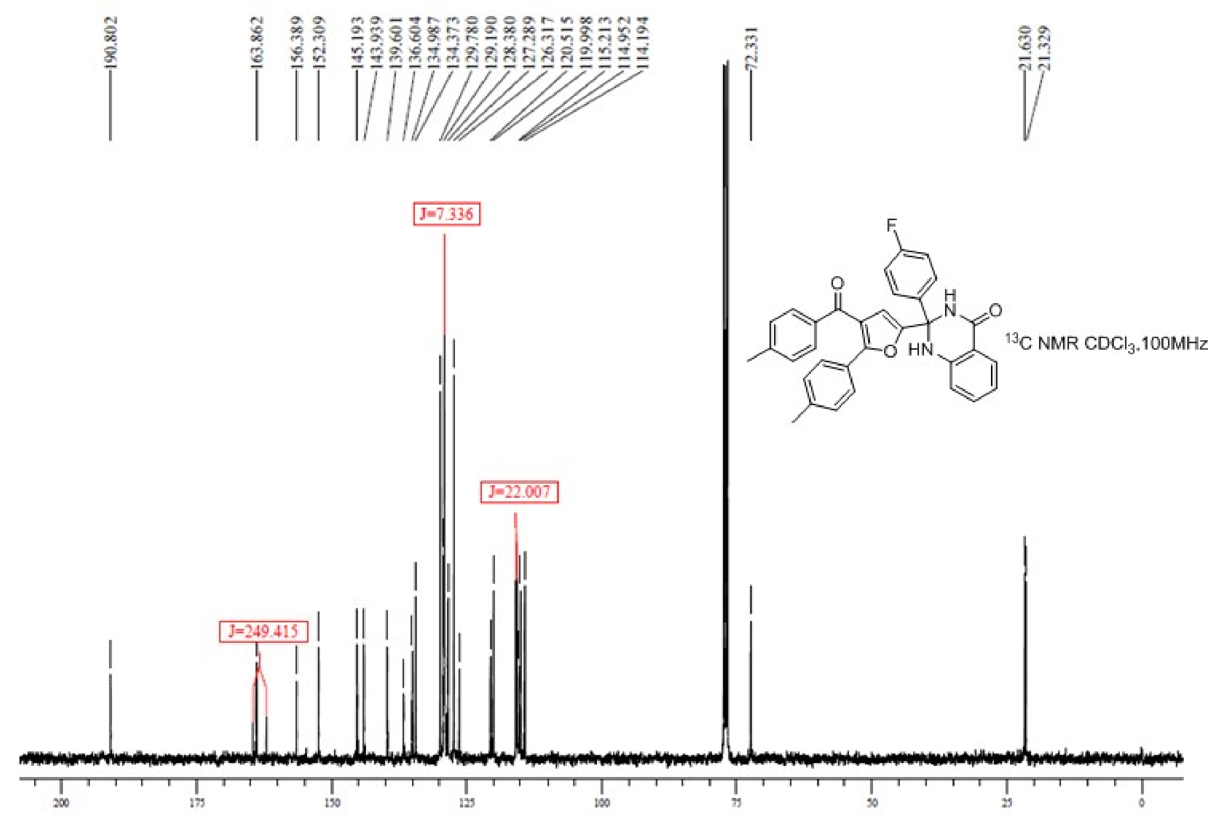

2-(4-Benzoyl-5-phenylfuran-2-yl)-2-(m-tolyl)-2,3-dihydroquinazolin-4(*1H*)-one: **3h**

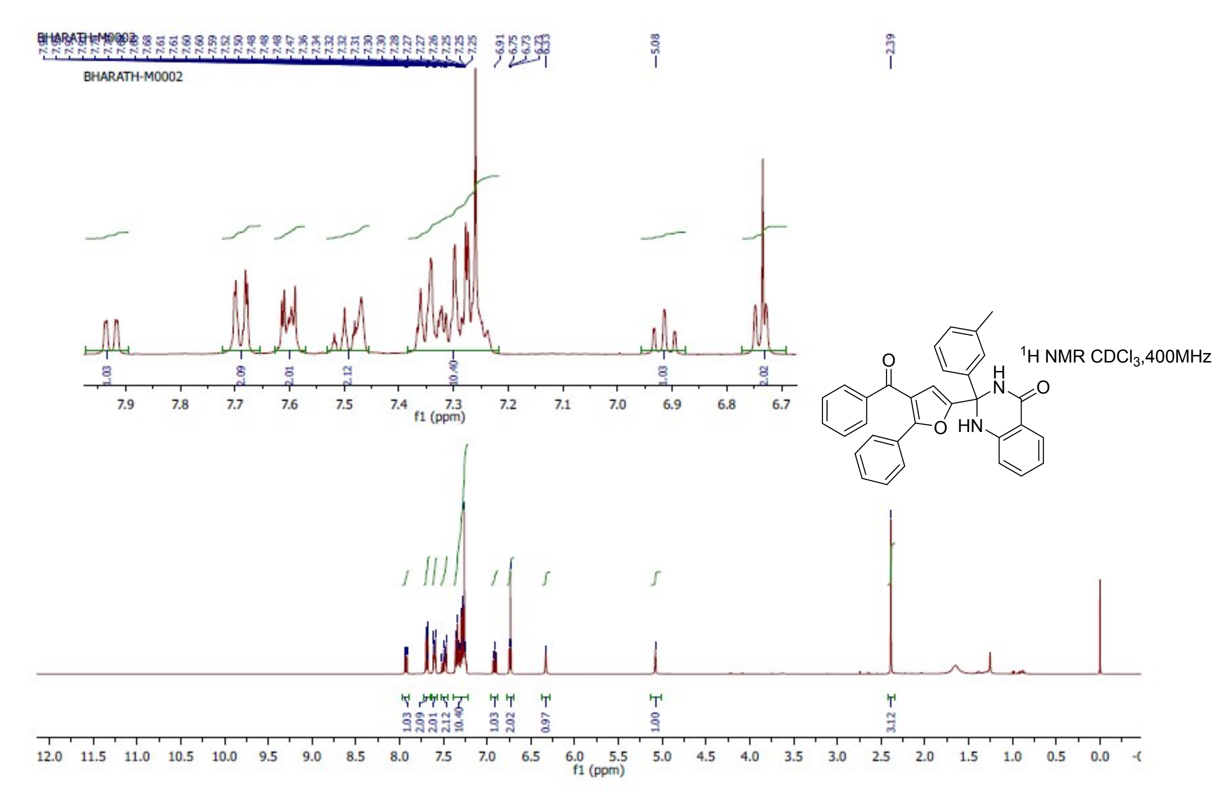

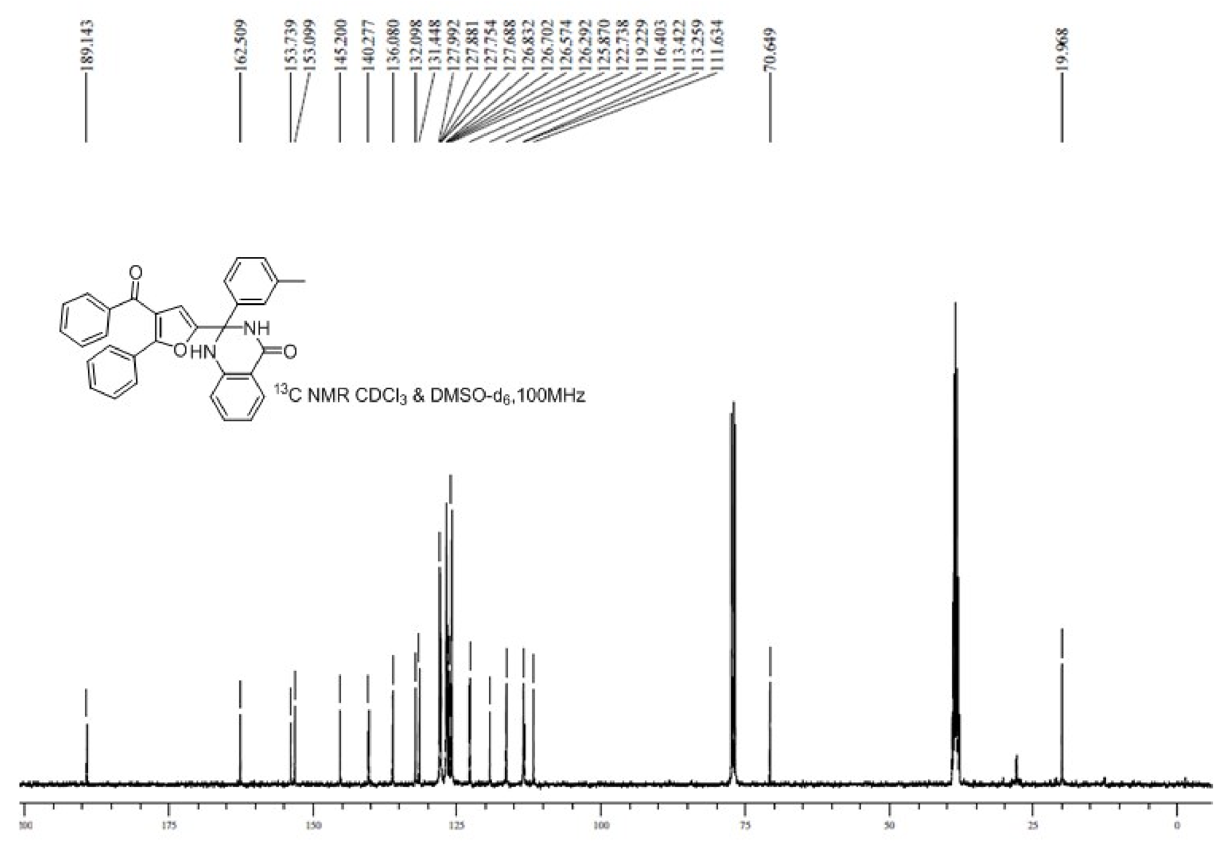

2-(4-(4-Methylbenzoyl)-5-(p-tolyl)furan-2-yl)-2-(m-tolyl)-2,3-dihydroquinazolin-4(*1H*)-one: **3i**

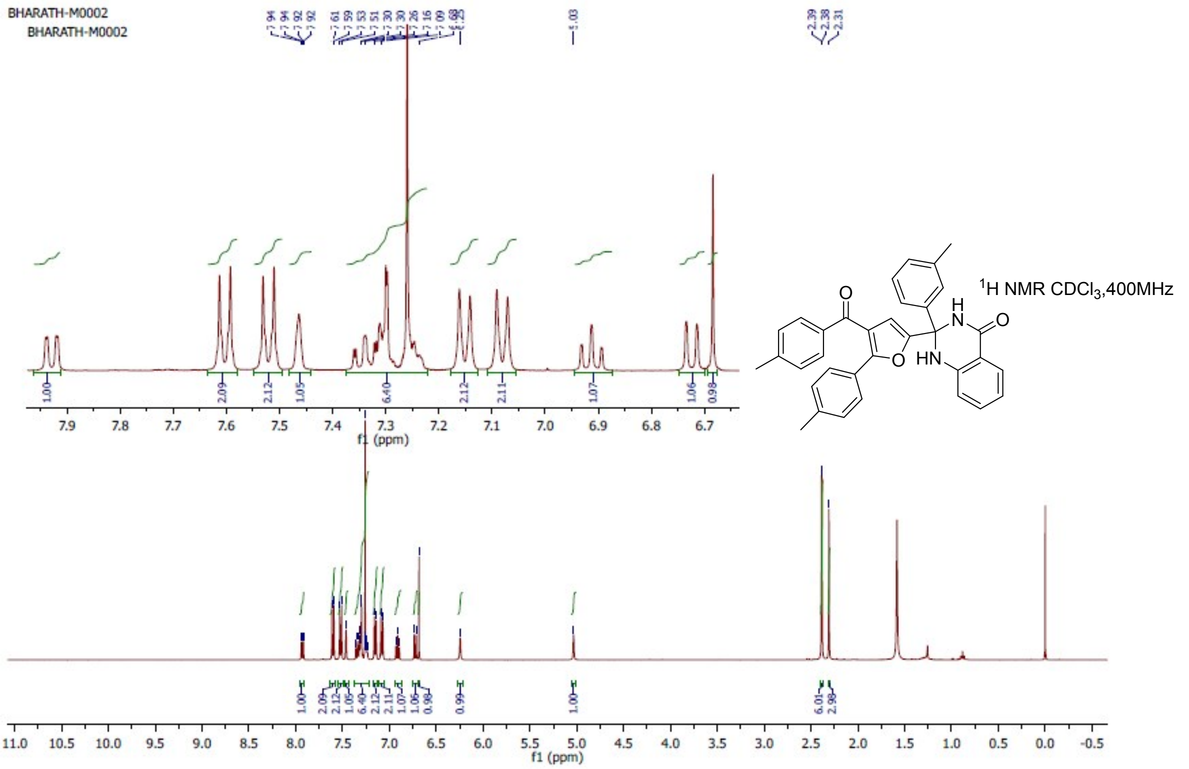

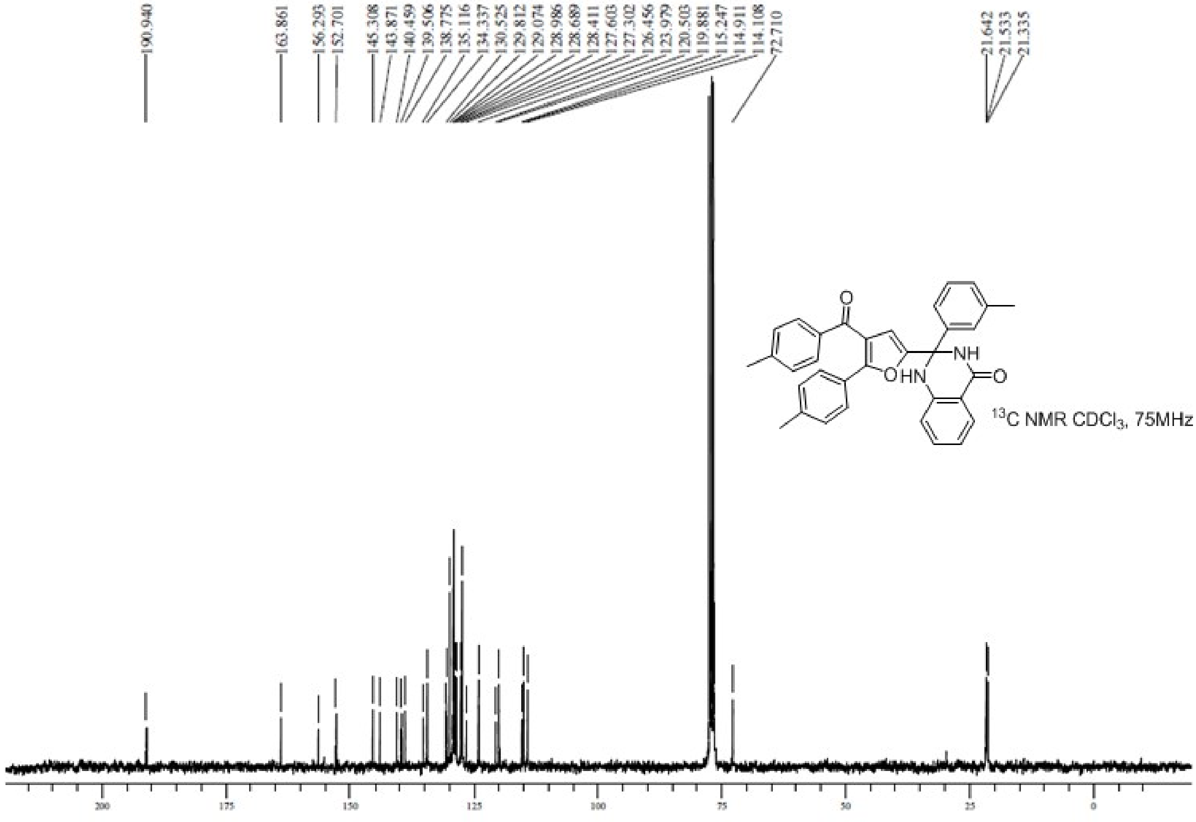

2-(4-(3-Methylbenzoyl)-5-(m-tolyl)furan-2-yl)-2-phenyl-2,3-dihydroquinazolin-4(*1H*)-one: **3j**

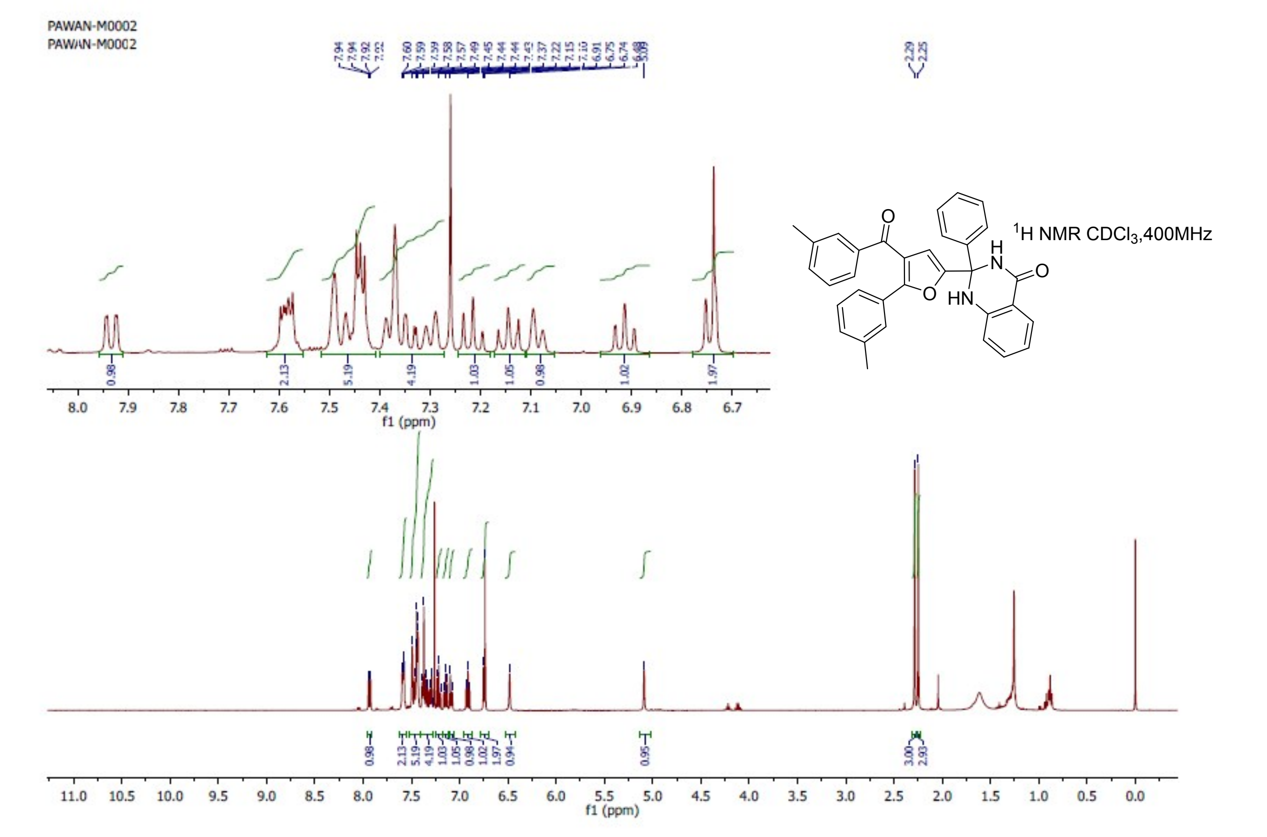

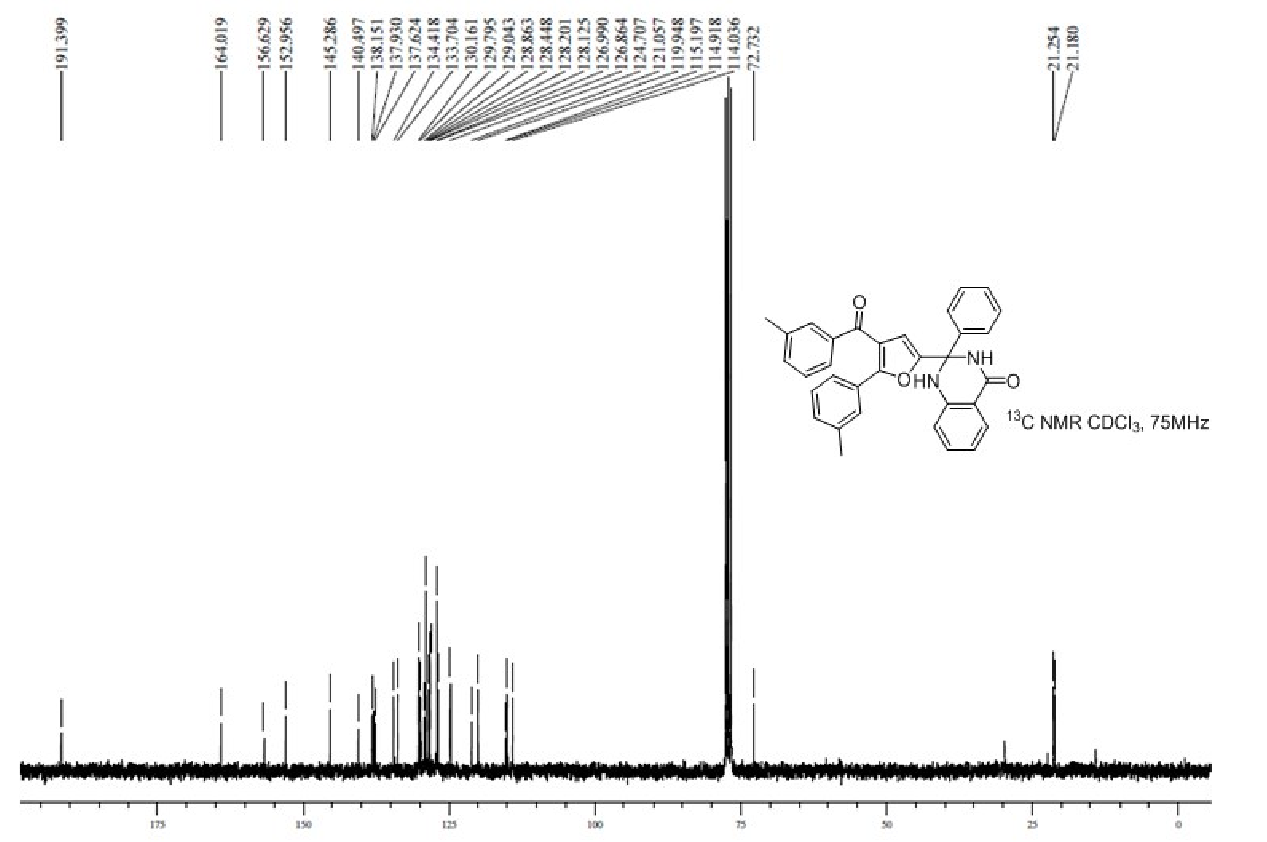

2-(4-Benzoyl-5-phenylfuran-2-yl)-2-hexyl-2,3-dihydroquinazolin-4(*1H*)-one: **3k**

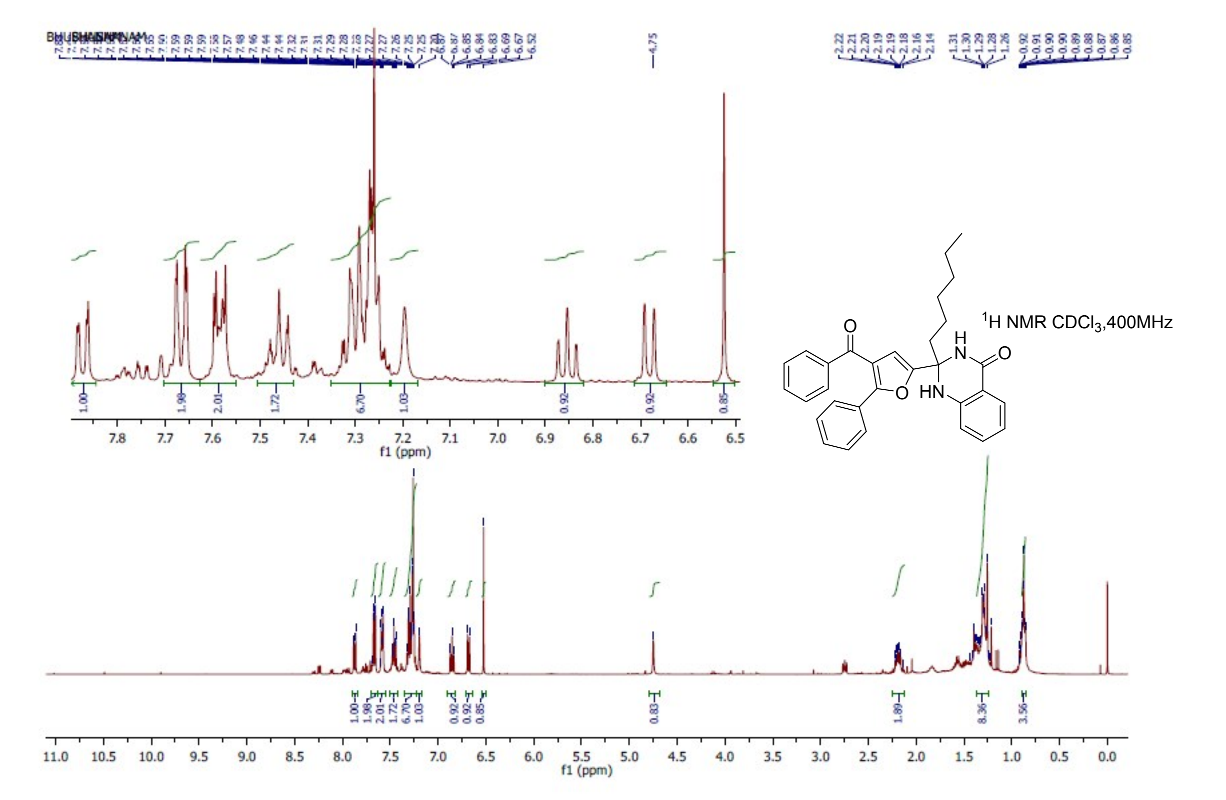

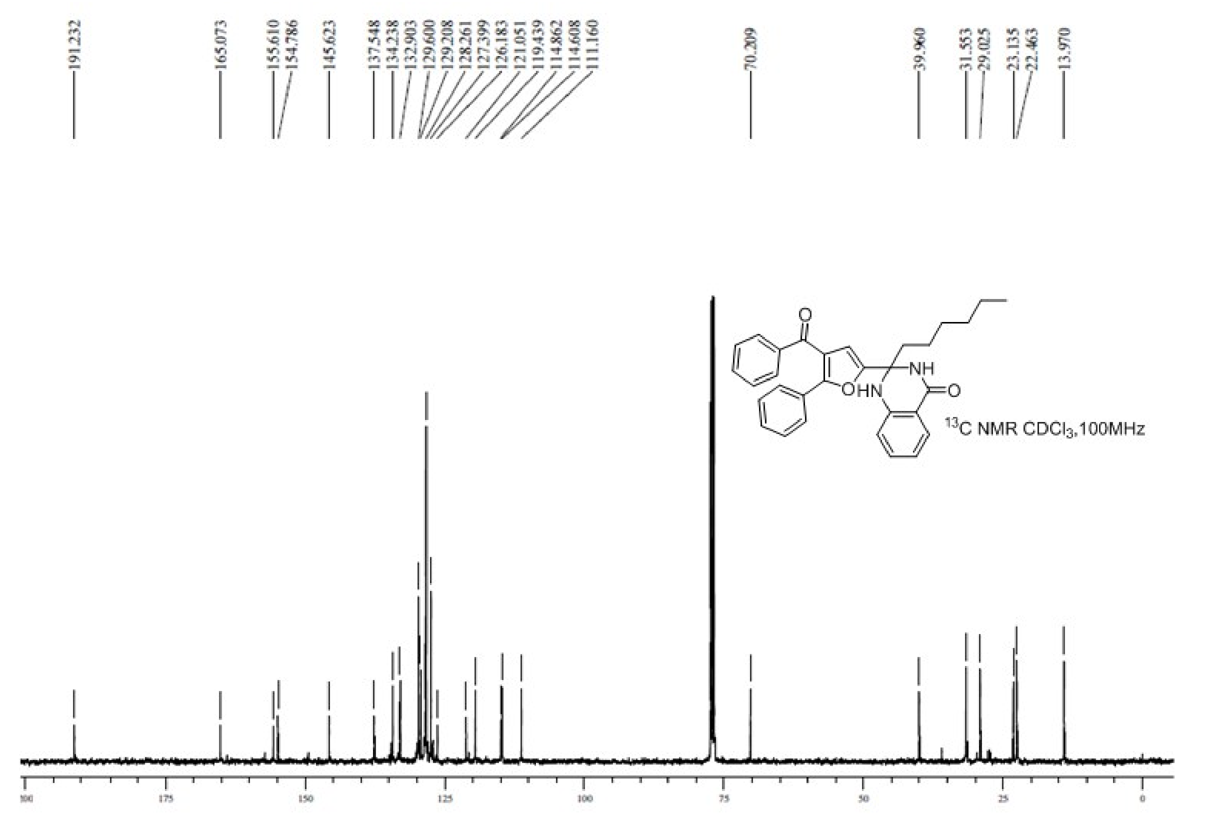

2-(4-Benzoyl-5-phenylfuran-2-yl)-5-methyl-2-phenyl-2,3-dihydroquinazolin-4(*1H*)-one:**3l**

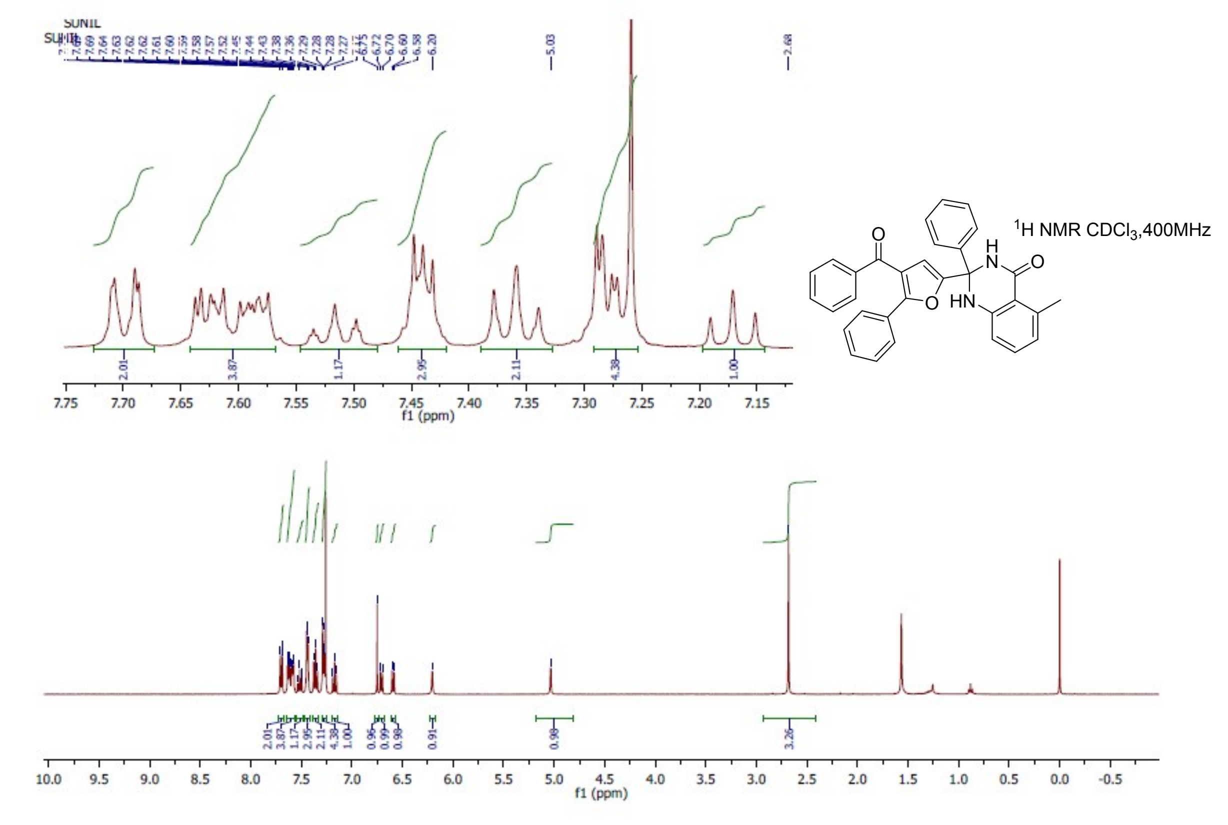

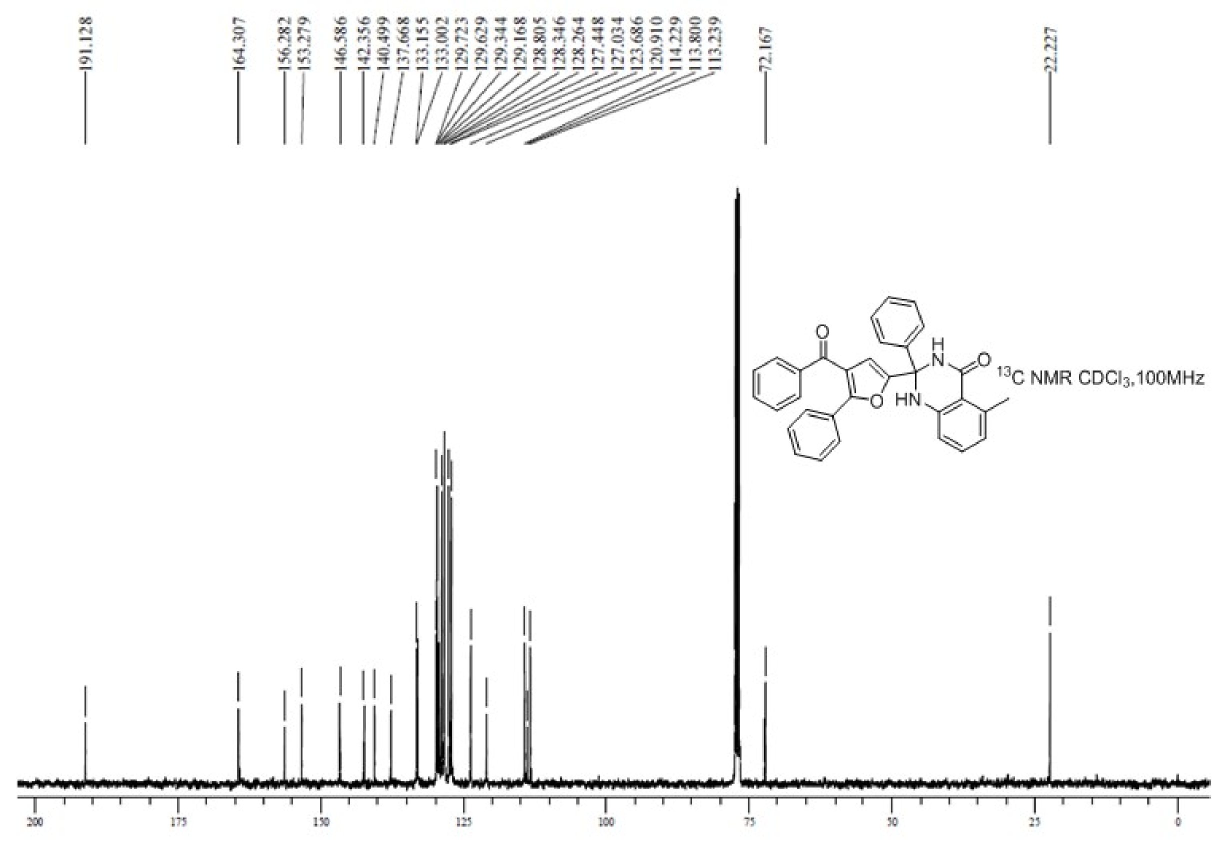

2-(4-Benzoyl-5-phenylfuran-2-yl)-6-chloro-2-phenyl-2,3-dihydroquinazolin-4(*1H*)-one:**3m**

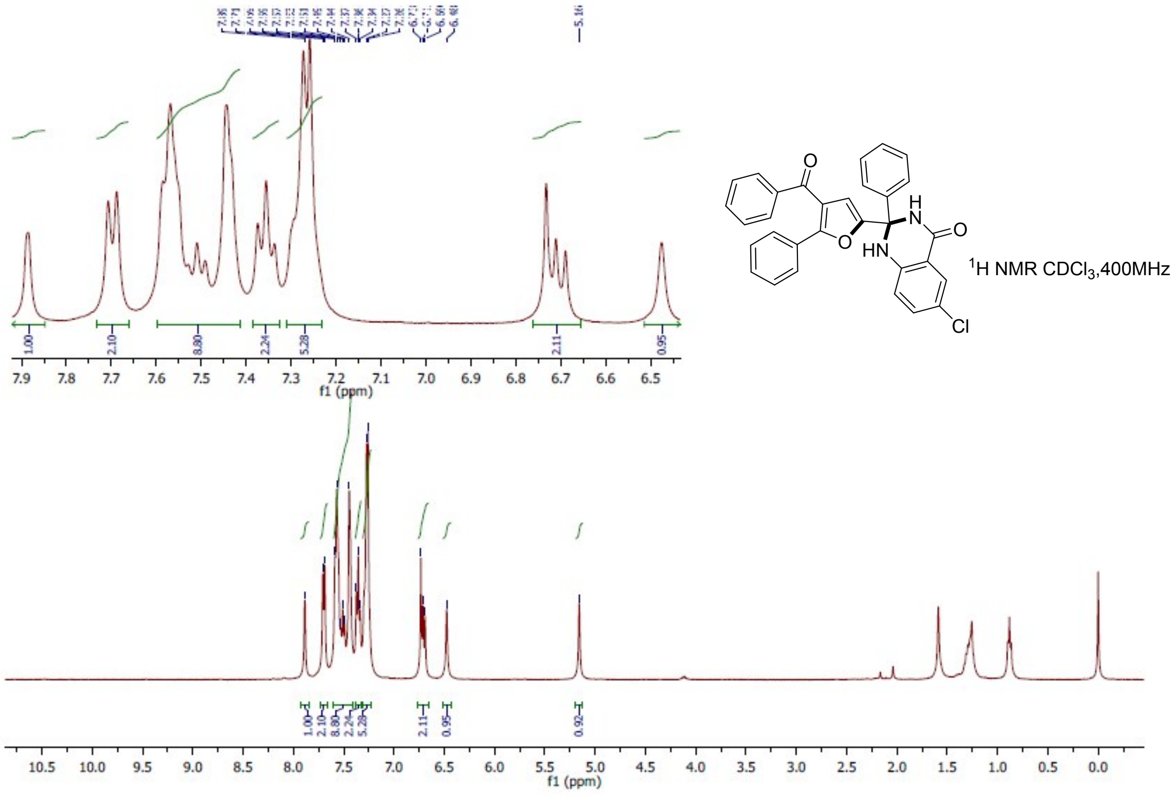

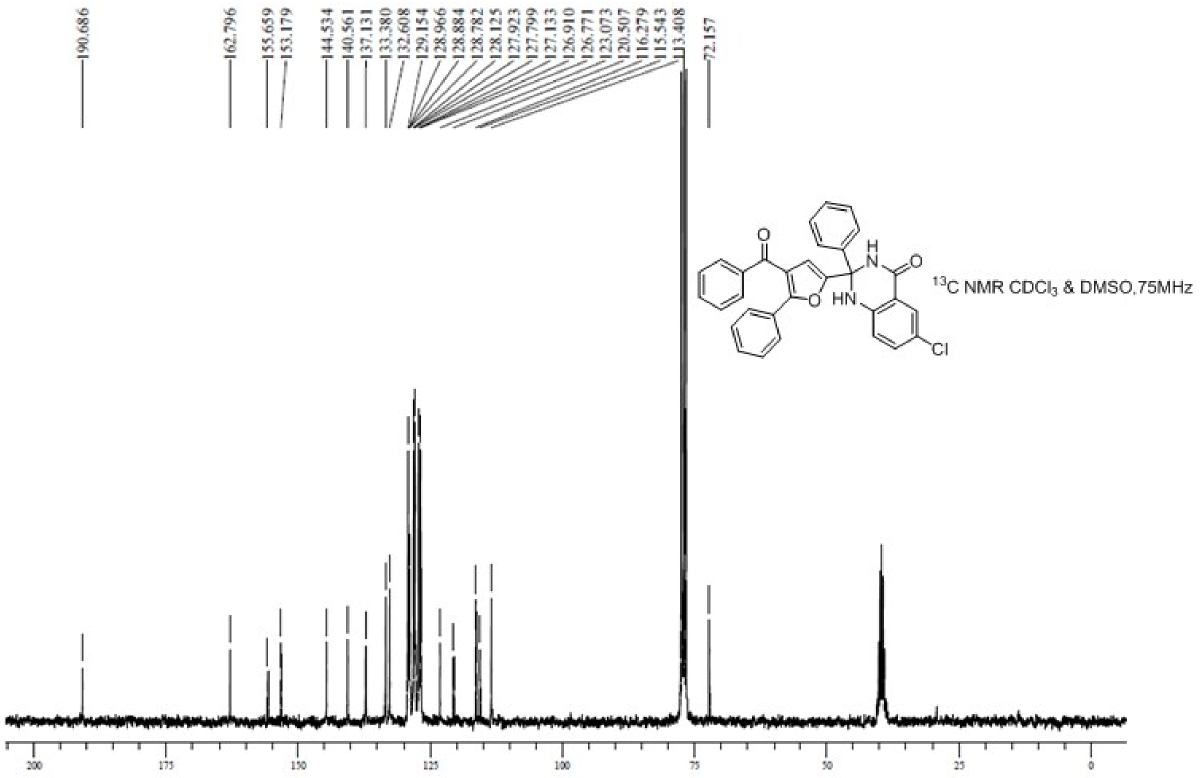

2-(4-Benzoyl-5-phenylfuran-2-yl)-6-bromo-2-phenyl-2,3-dihydroquinazolin-4(*1H*)-one:**3n**

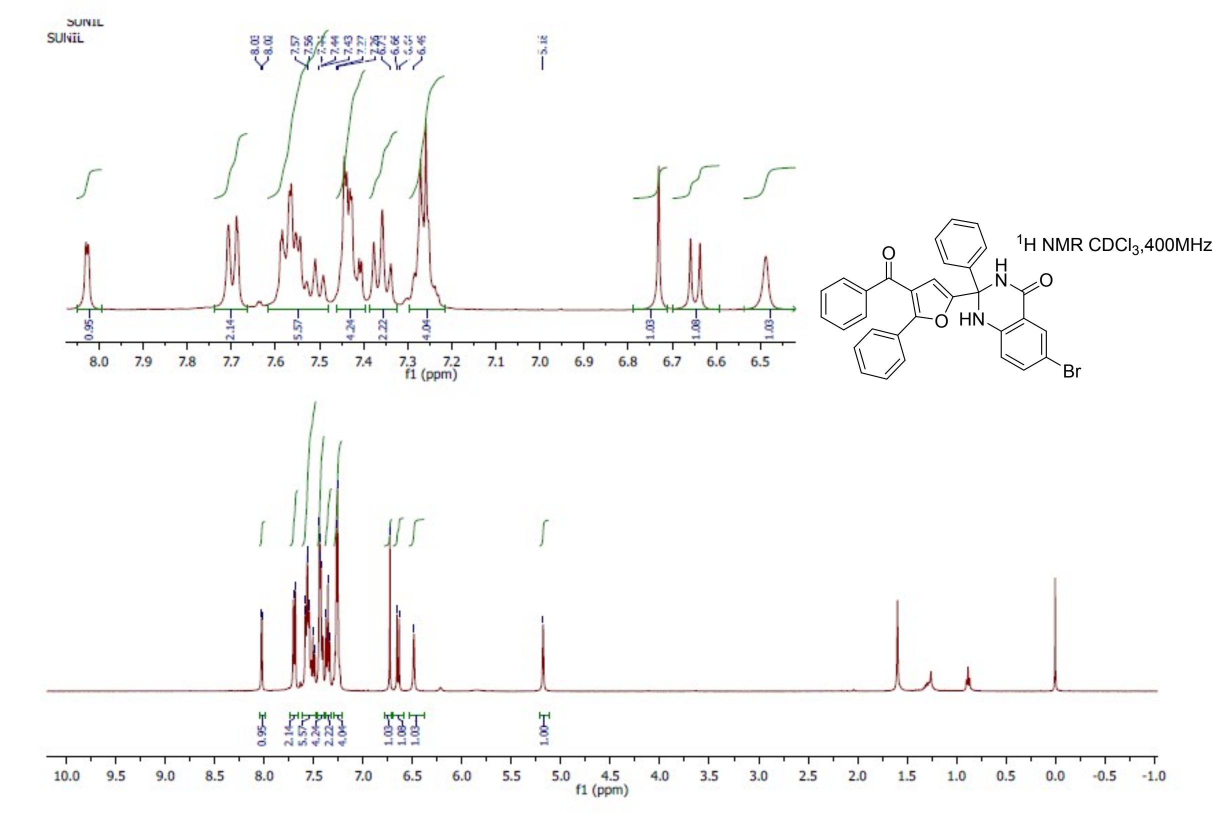

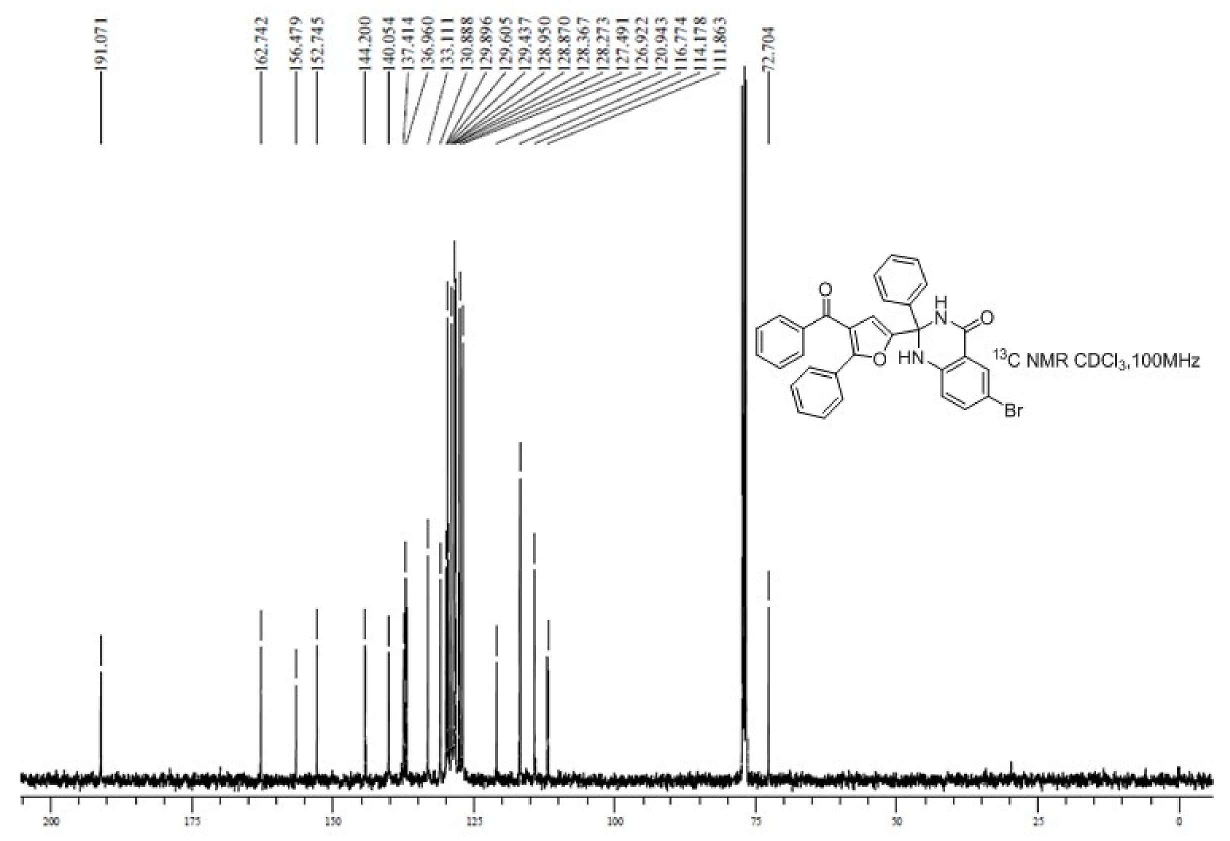

2-(4-Benzoyl-5-phenylfuran-2-yl)-6-iodo-2-phenyl-2,3-dihydroquinazolin-4(*1H*)-one:**3o**

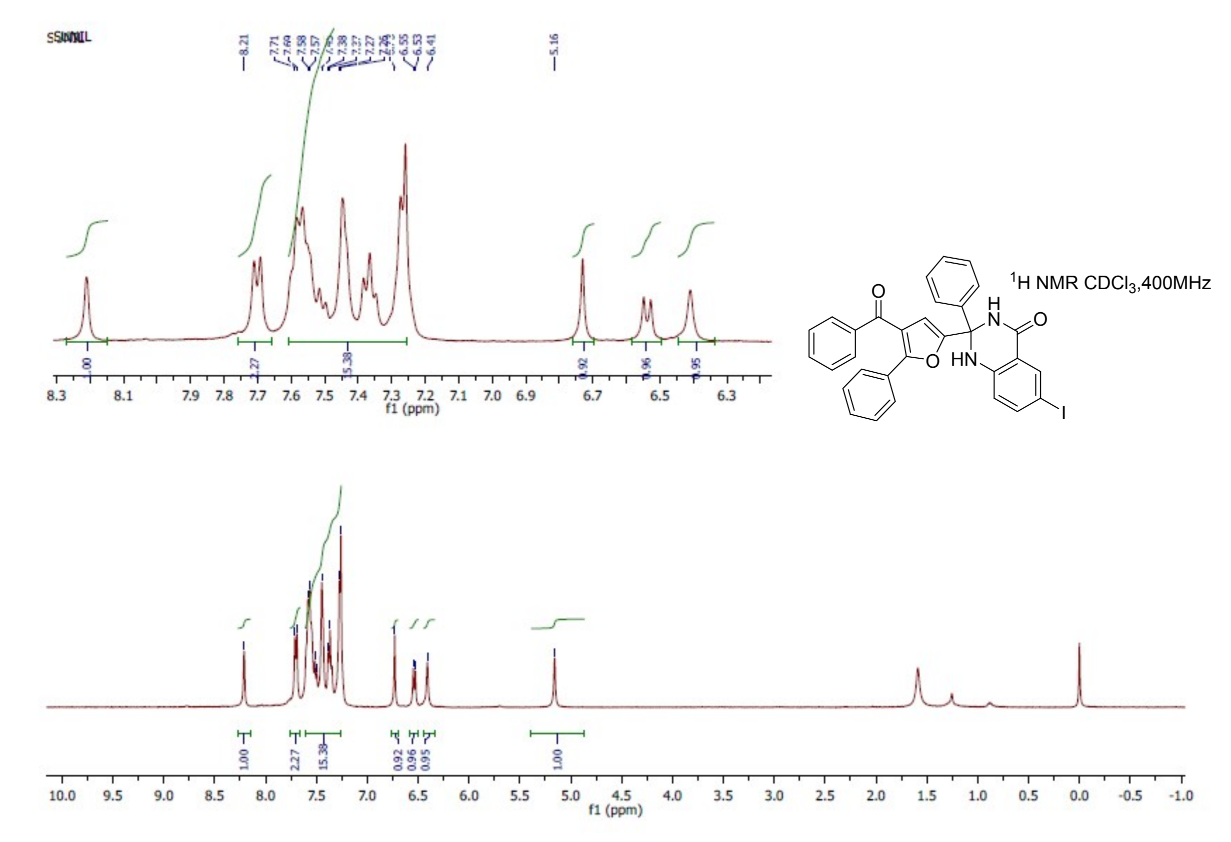

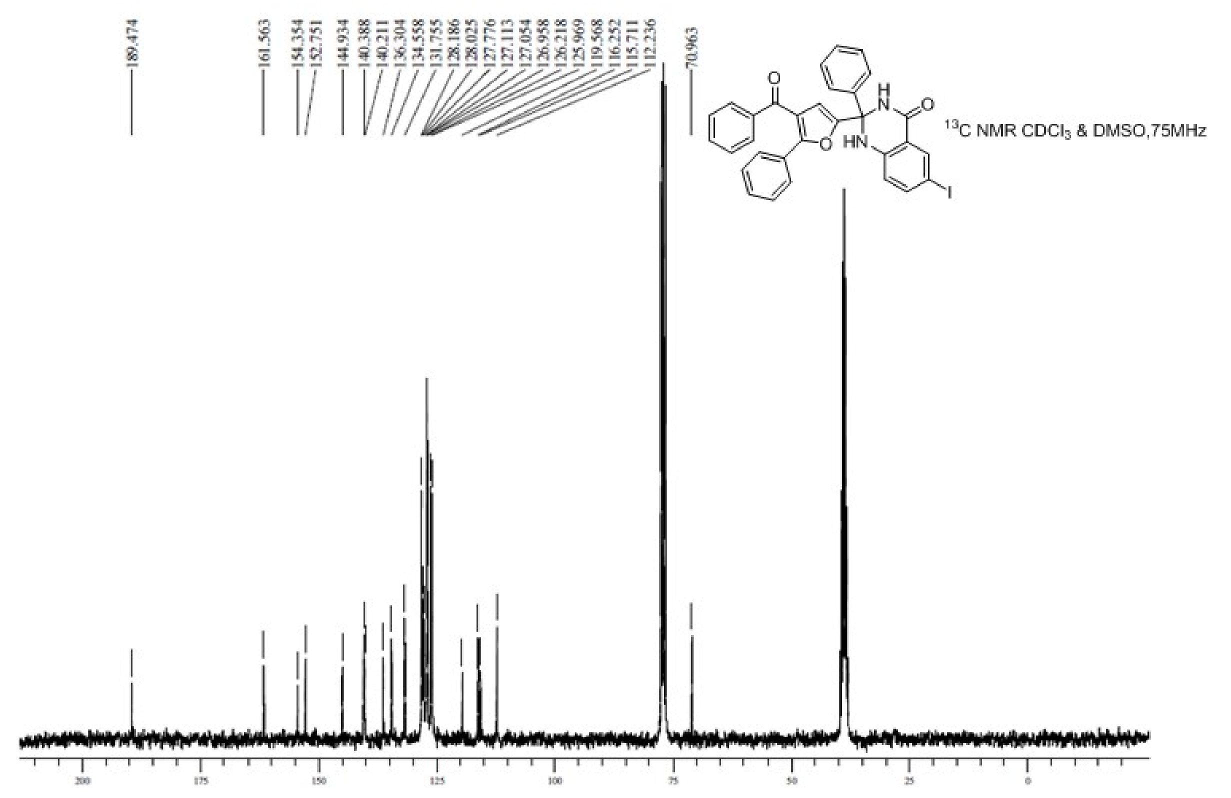

2-(4-Benzoyl-5-phenylfuran-2-yl)-5-methyl-2-(*p*-tolyl)-2,3-dihydroquinazolin-4(*1H*)-one:**3p**

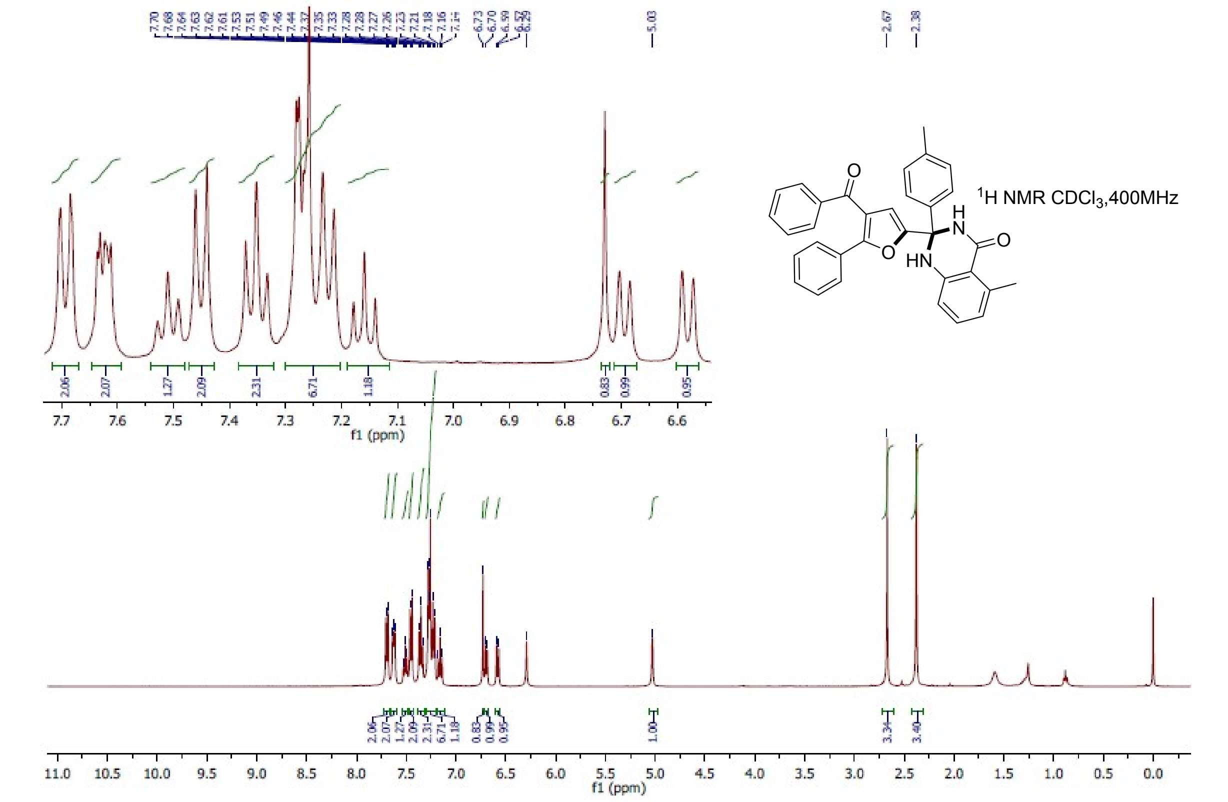

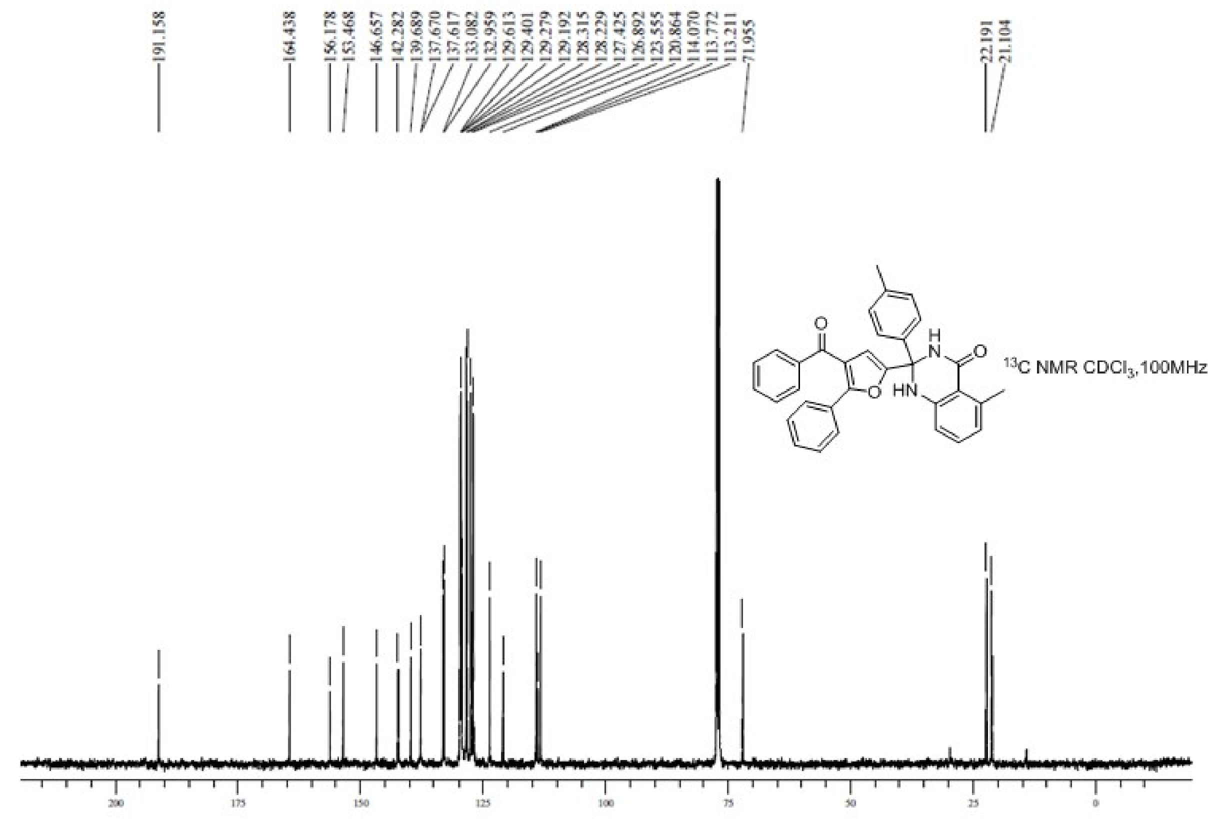

2-(4-Benzoyl-5-phenylfuran-2-yl)-2-(4-methoxyphenyl)-5-methyl-2,3-dihydroquinazolin-4(*1H*)-one:**3q**

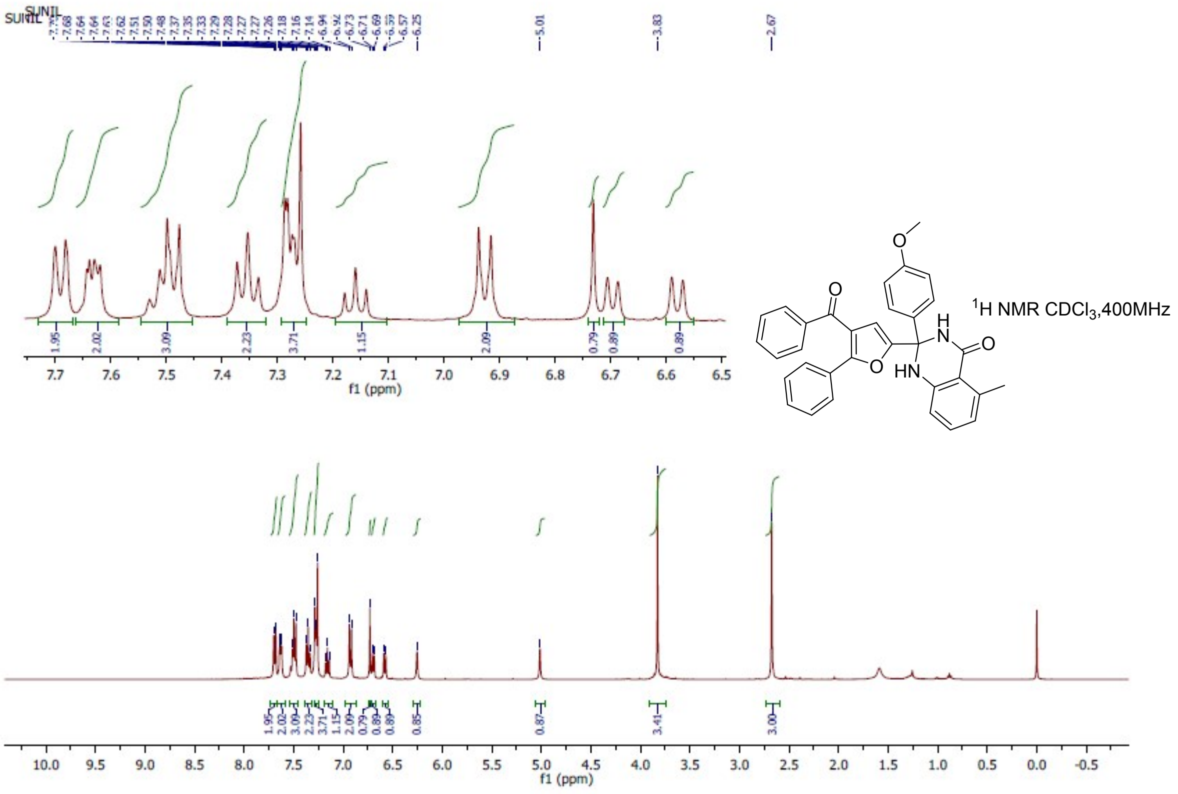

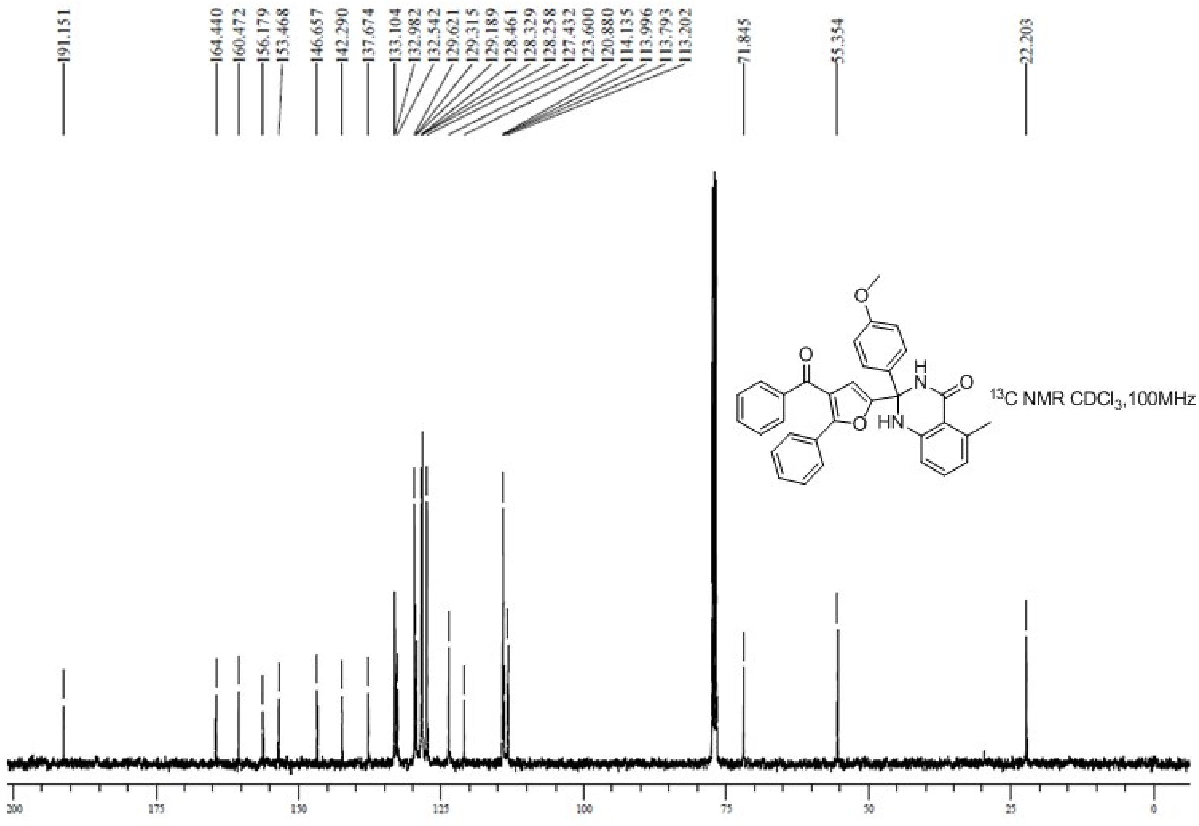

2-(4-Benzoyl-5-phenylfuran-2-yl)-2-(4-fluorophenyl)-5-methyl-2,3-dihydroquinazolin-4(*1H*)-one:**3r**

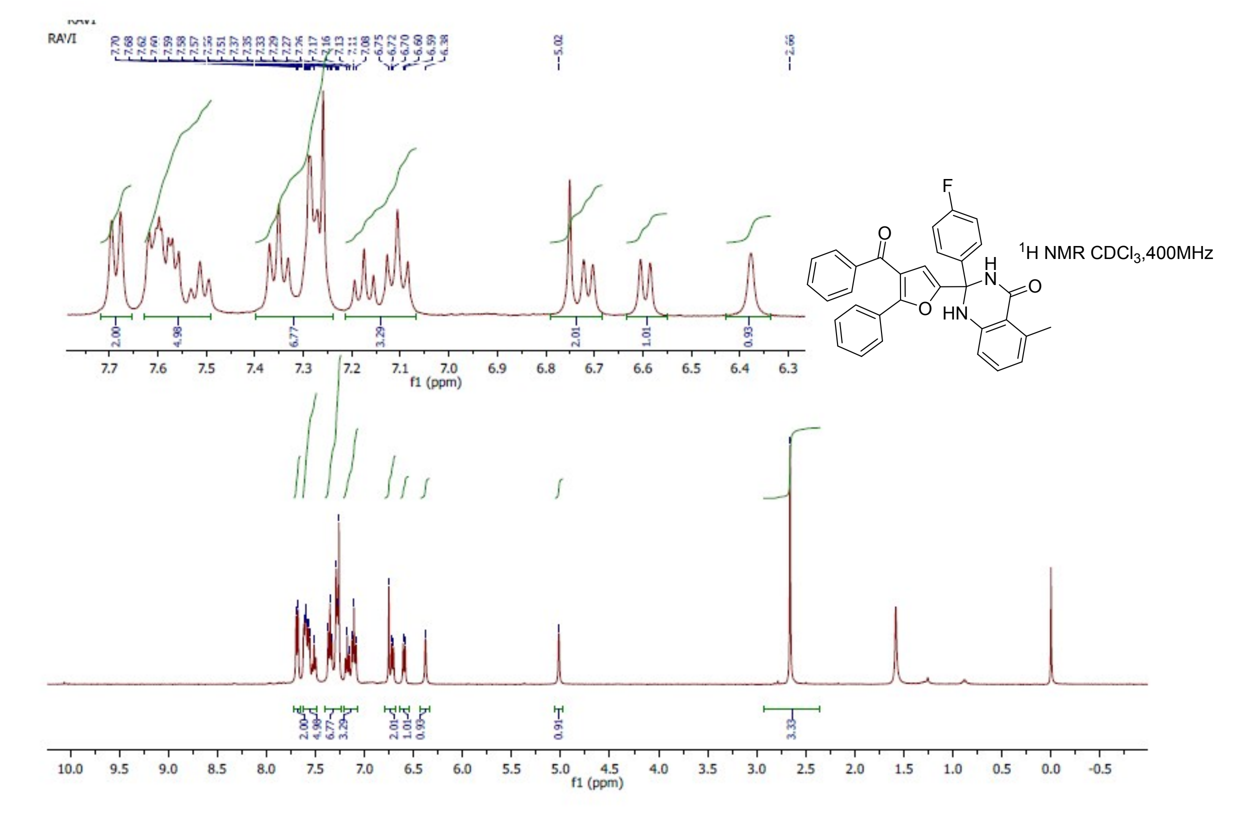

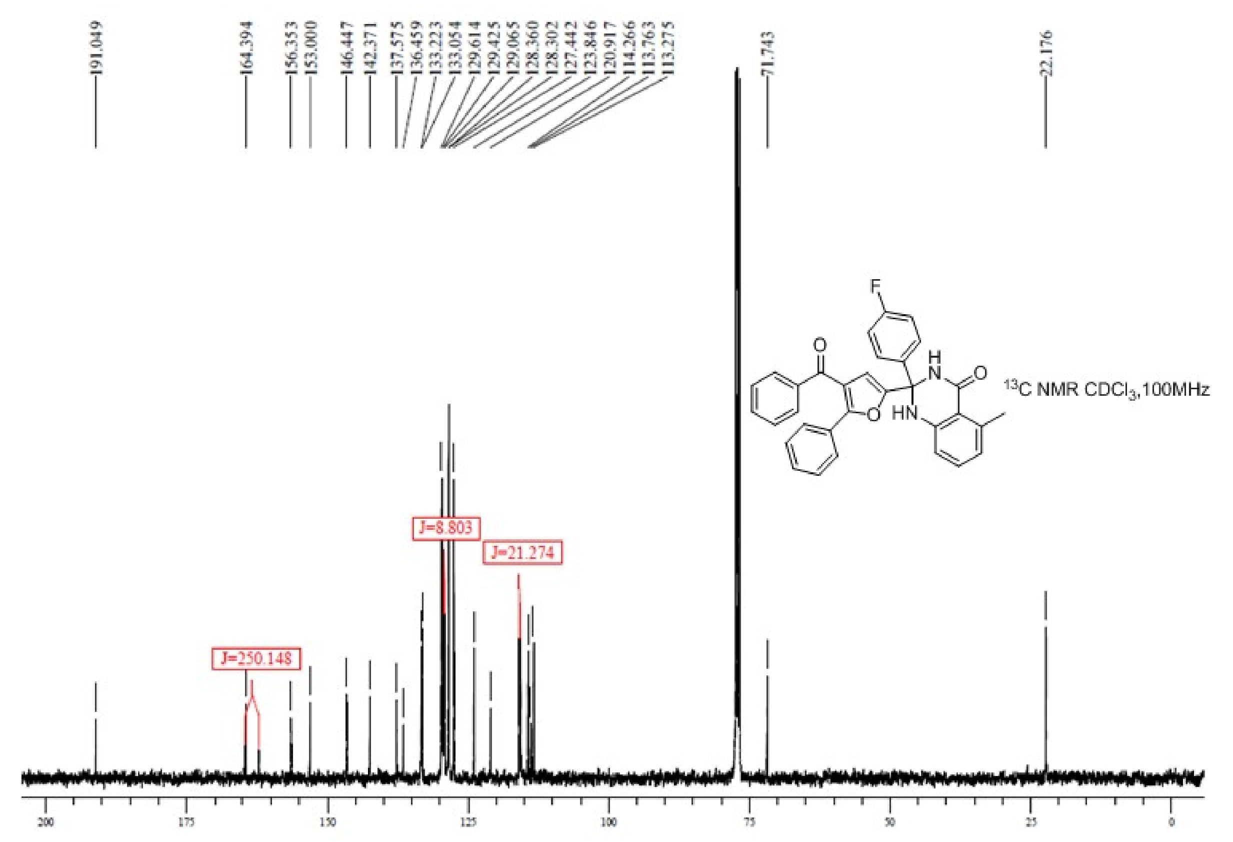

2-(4-Benzoyl-5-phenylfuran-2-yl)-6-chloro-2-(4-fluorophenyl)-2,3-dihydroquinazolin-4(*1H*)-one:**3s**

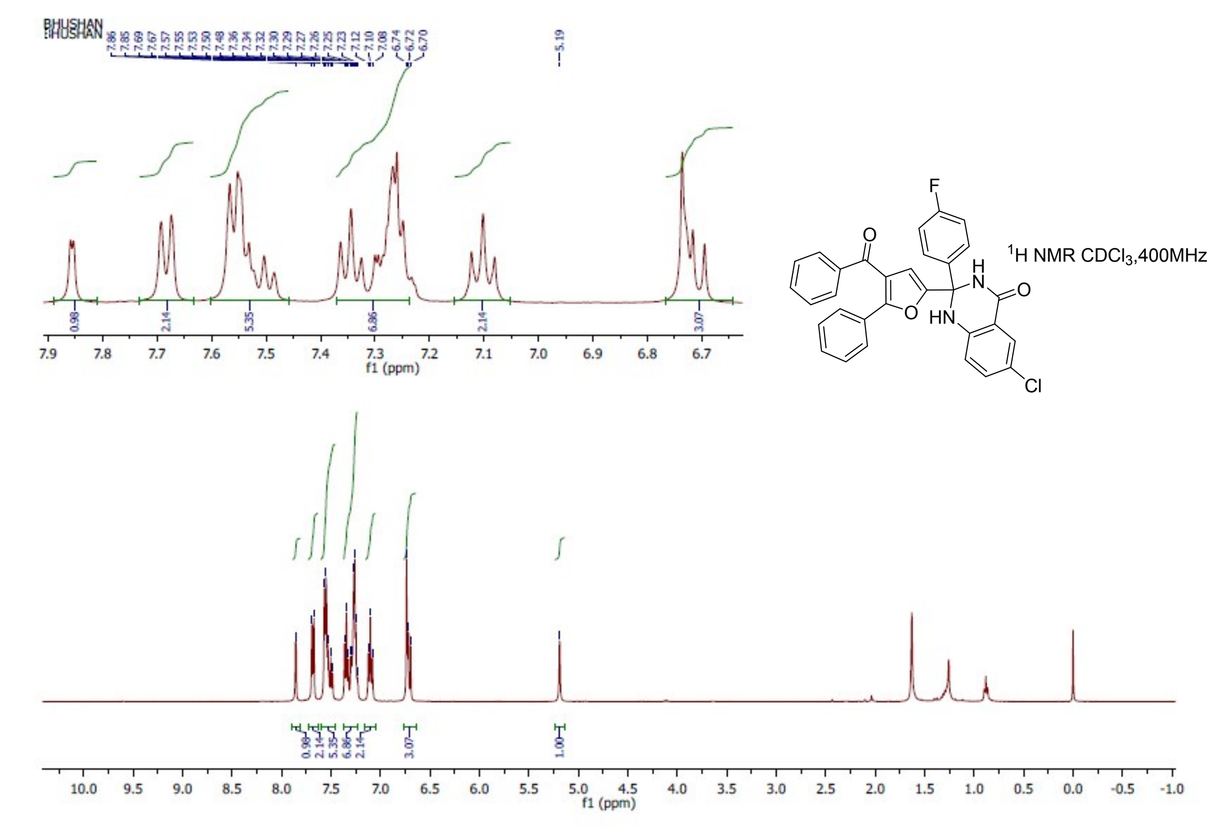

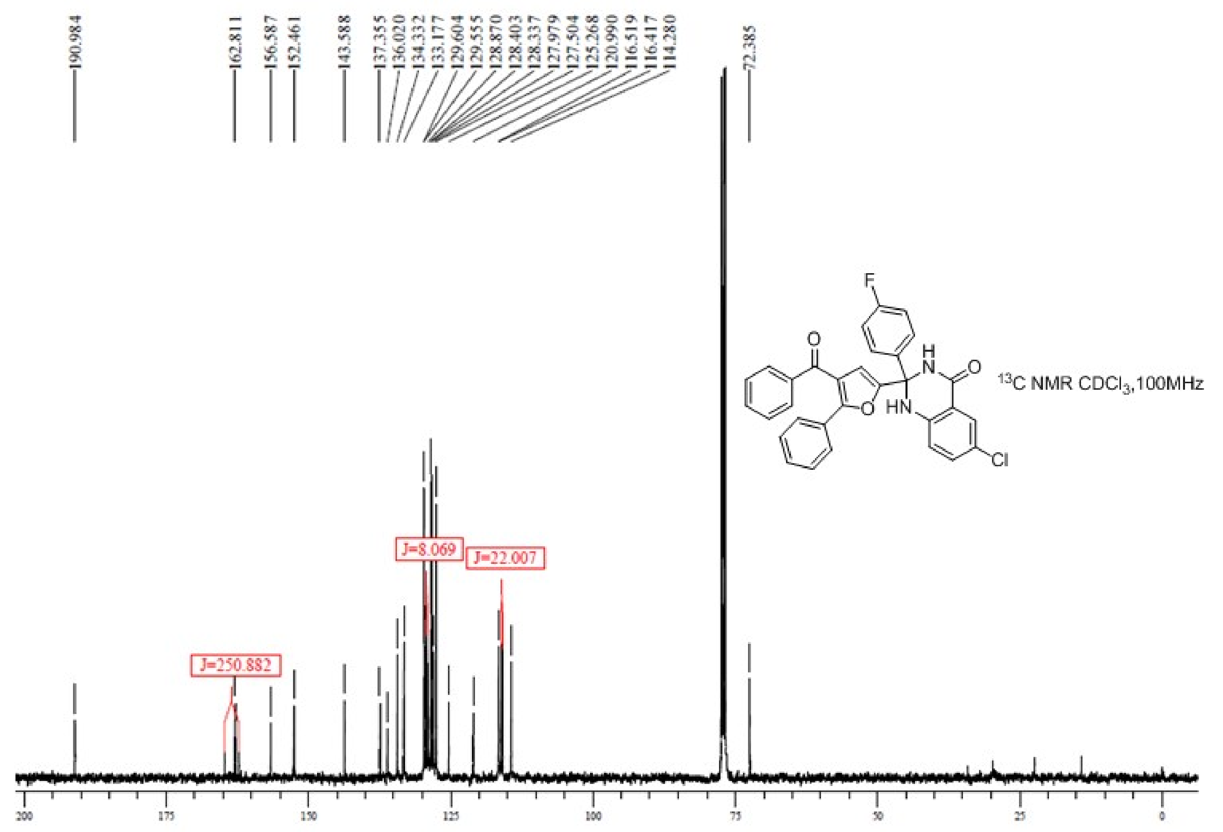

2-(4-Benzoyl-5-phenylfuran-2-yl)-6-bromo-2-(4-fluorophenyl)-2,3-dihydroquinazolin-4(*1H*)-one:**3t**

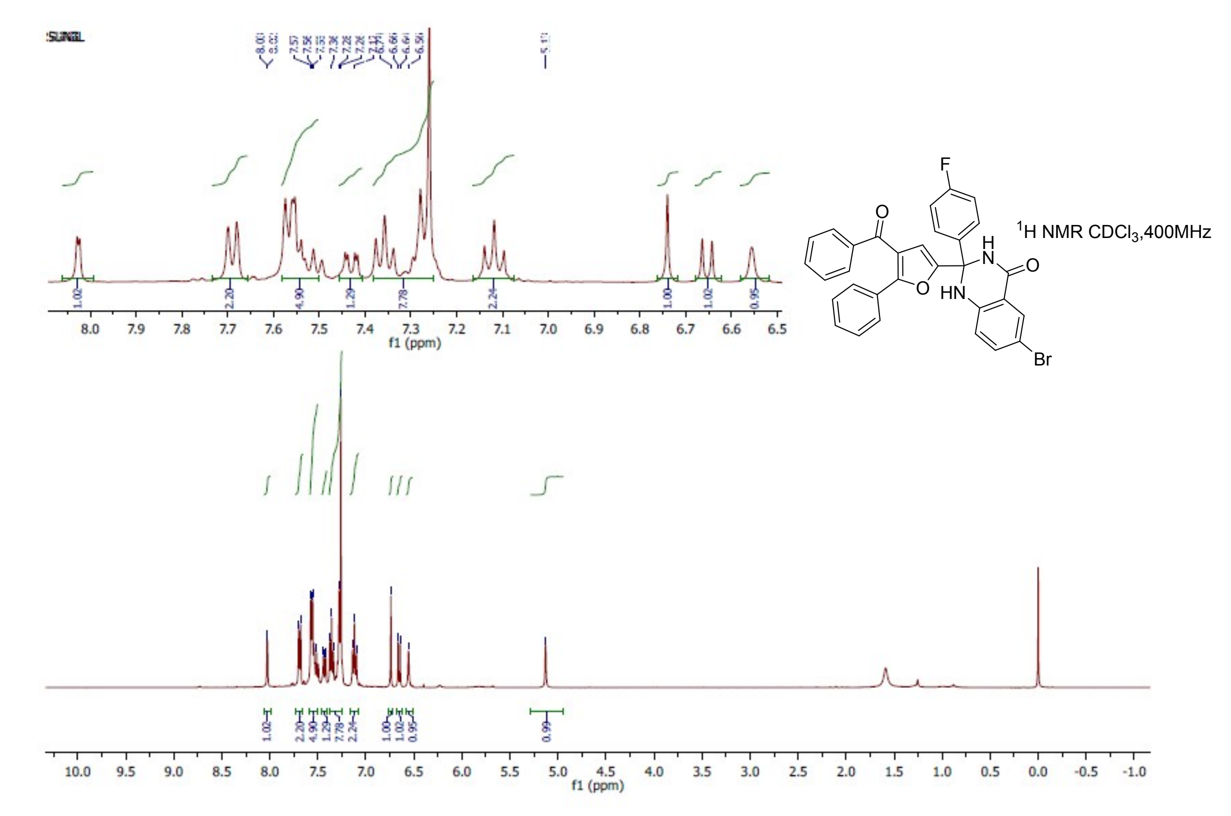

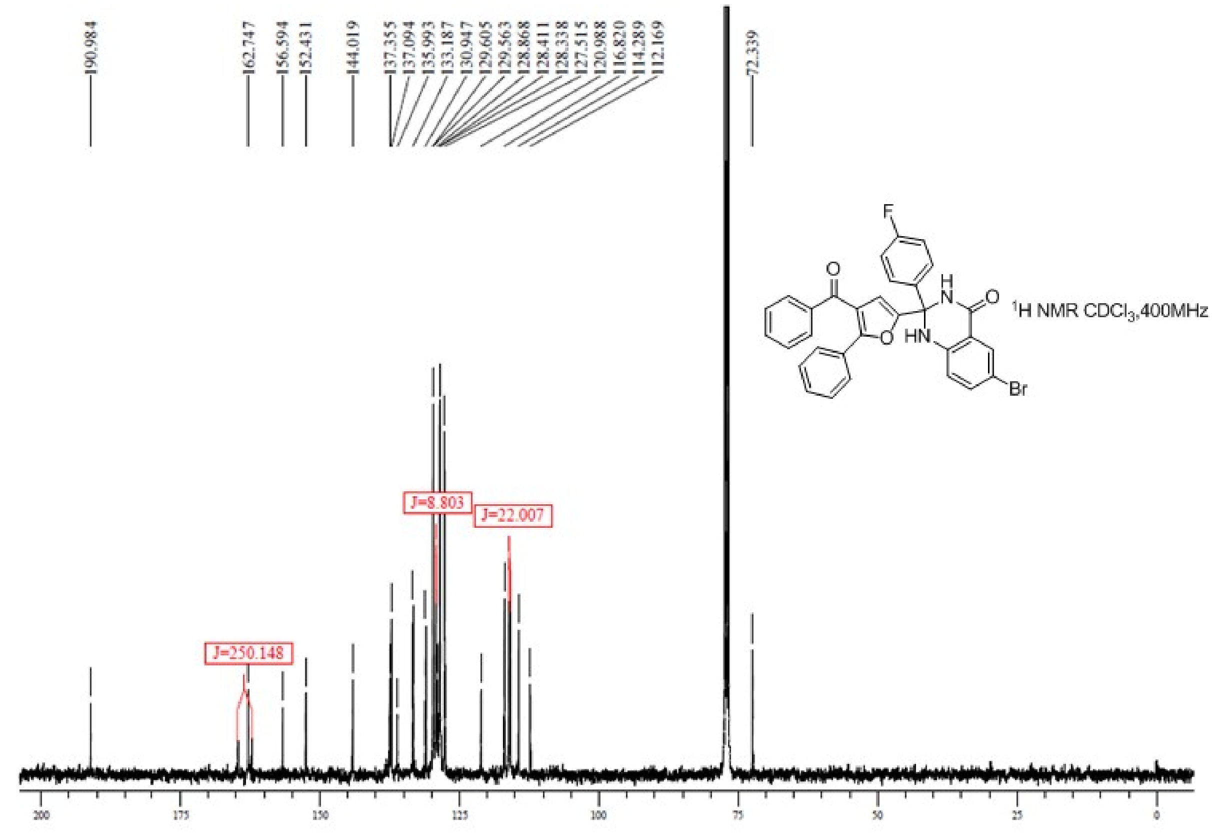

2-(4-Benzoyl-5-phenylfuran-2-yl)-2-(4-fluorophenyl)-6-iodo-2,3-dihydroquinazolin-4(*1H*)-one:**3u**

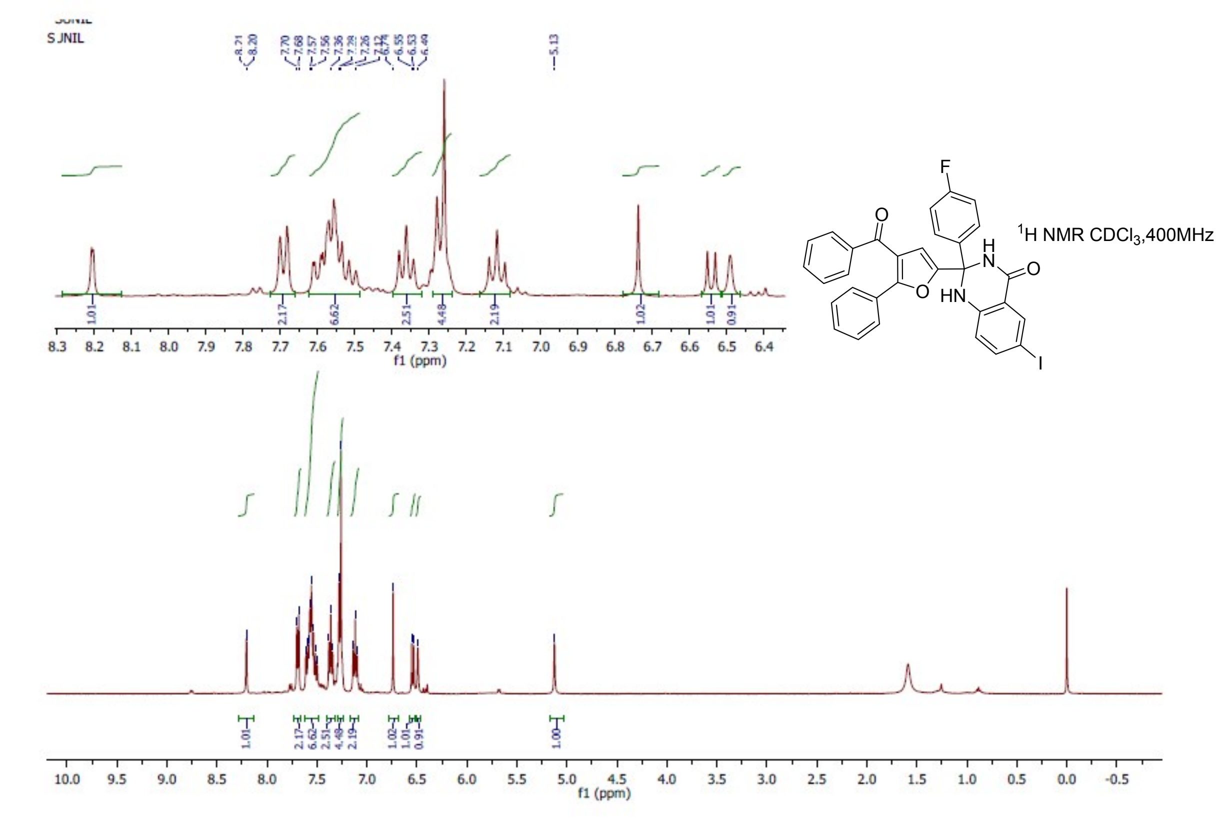

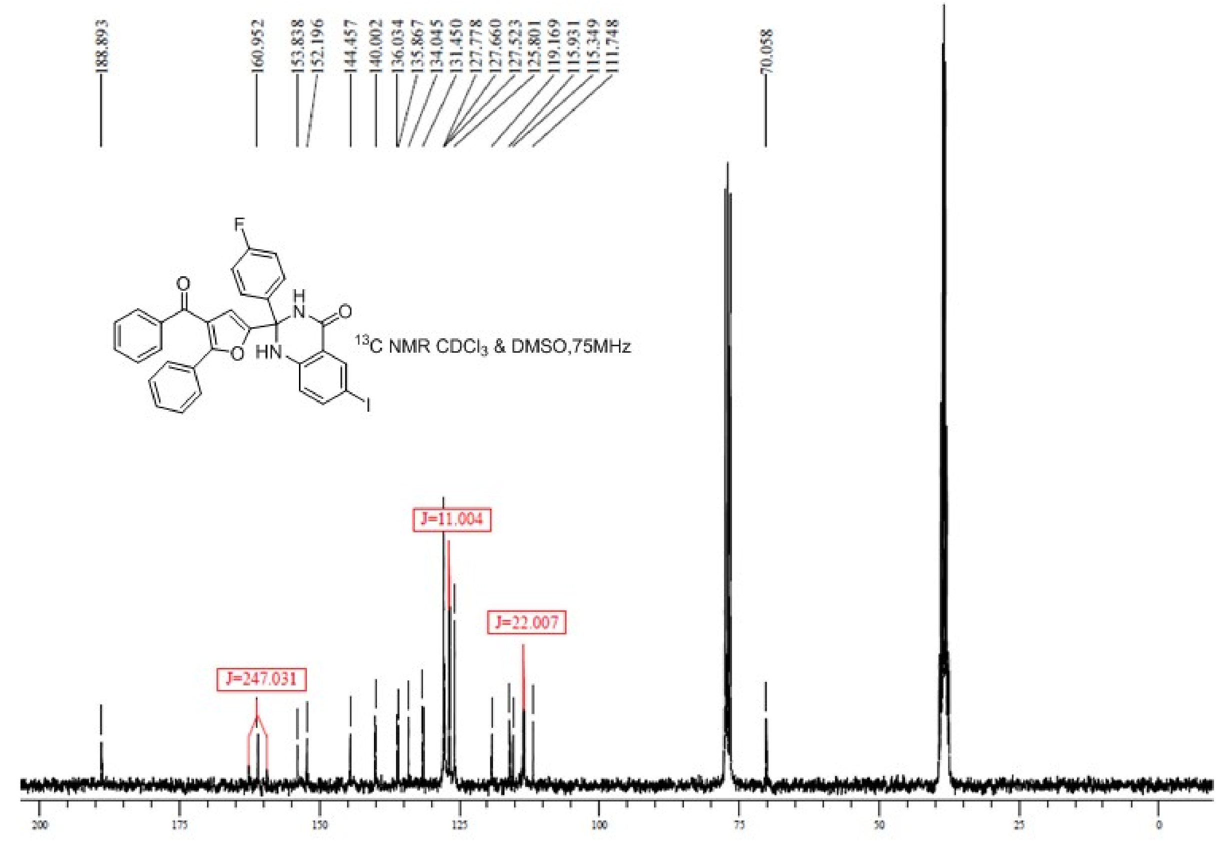

5-Methyl-2-(4-(4-methylbenzoyl)-5-(*p*-tolyl)furan-2-yl)-2-phenyl-2,3-dihydroquinazolin-4(*1H*)-one: **3v**

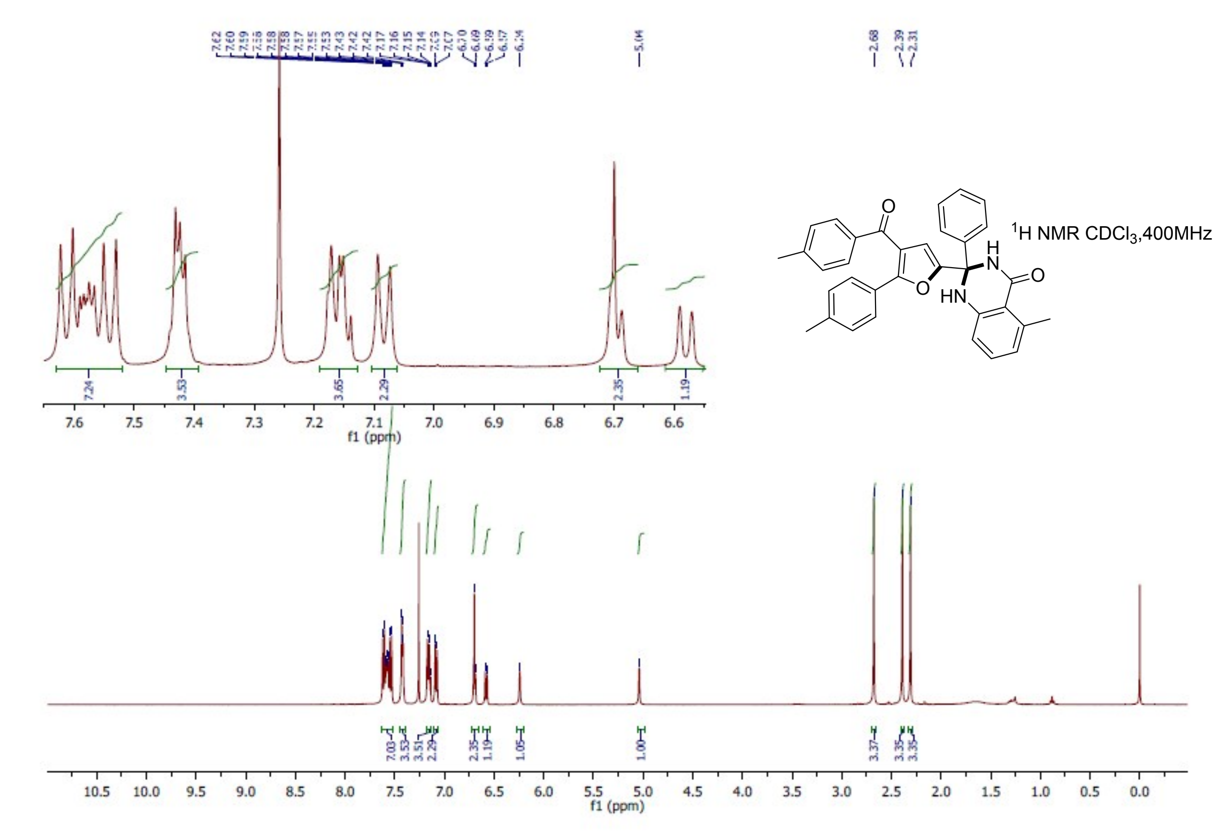

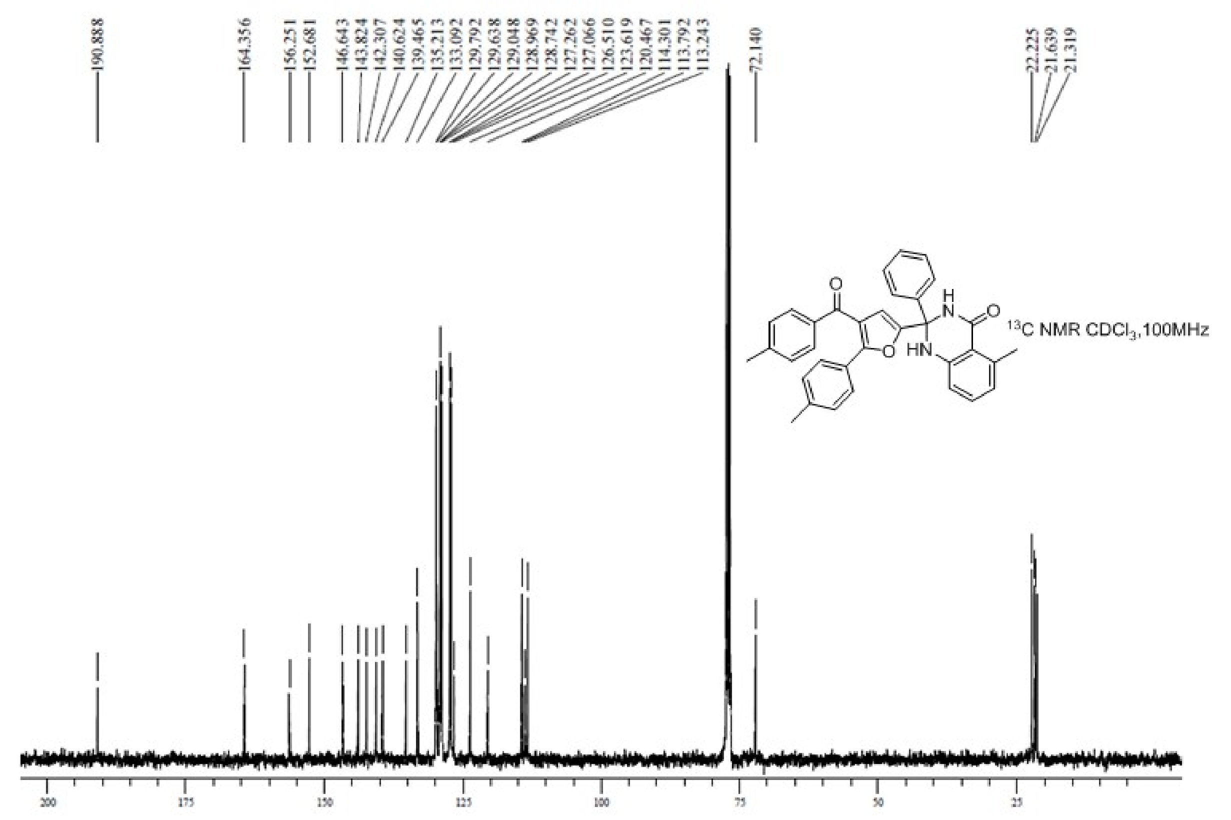

5-Methyl-2-(4-(4-methylbenzoyl)-5-(*p*-tolyl)furan-2-yl)-2-(*p*-tolyl)-2,3-dihydroquinazolin-4(*1H*)-one:**3w**

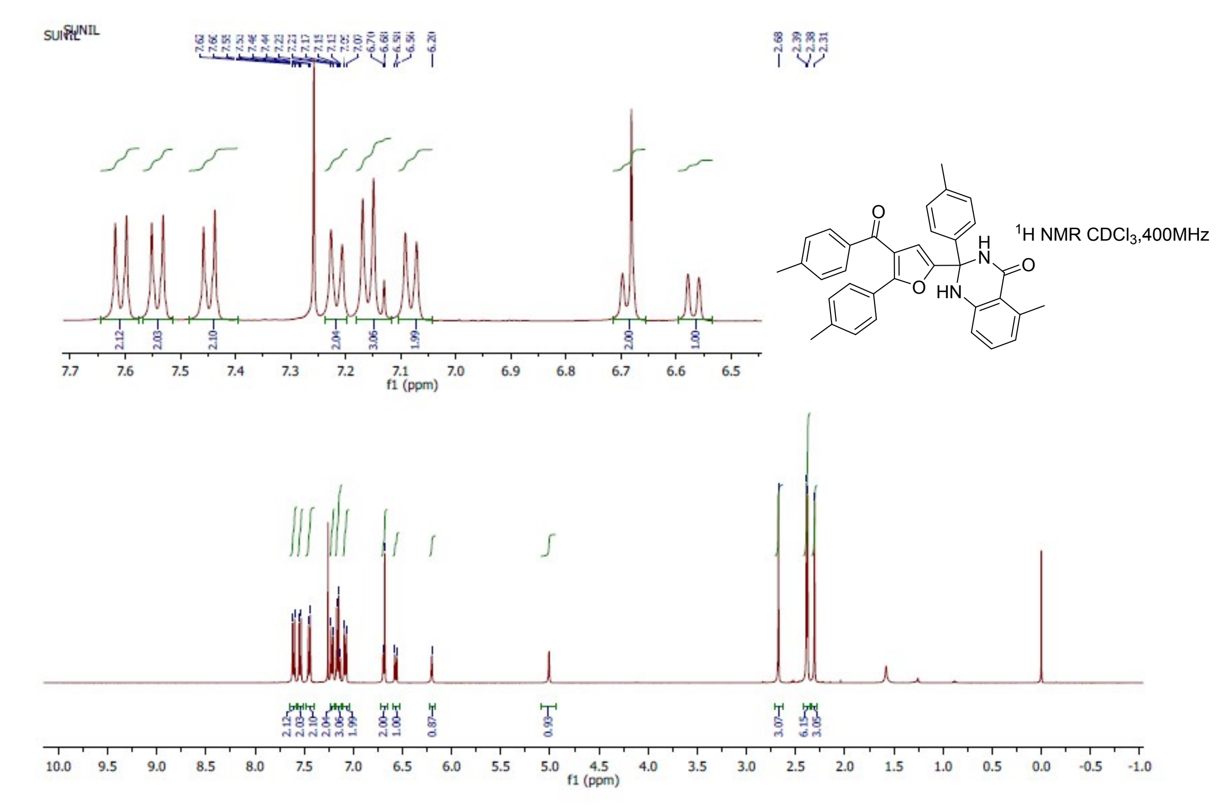

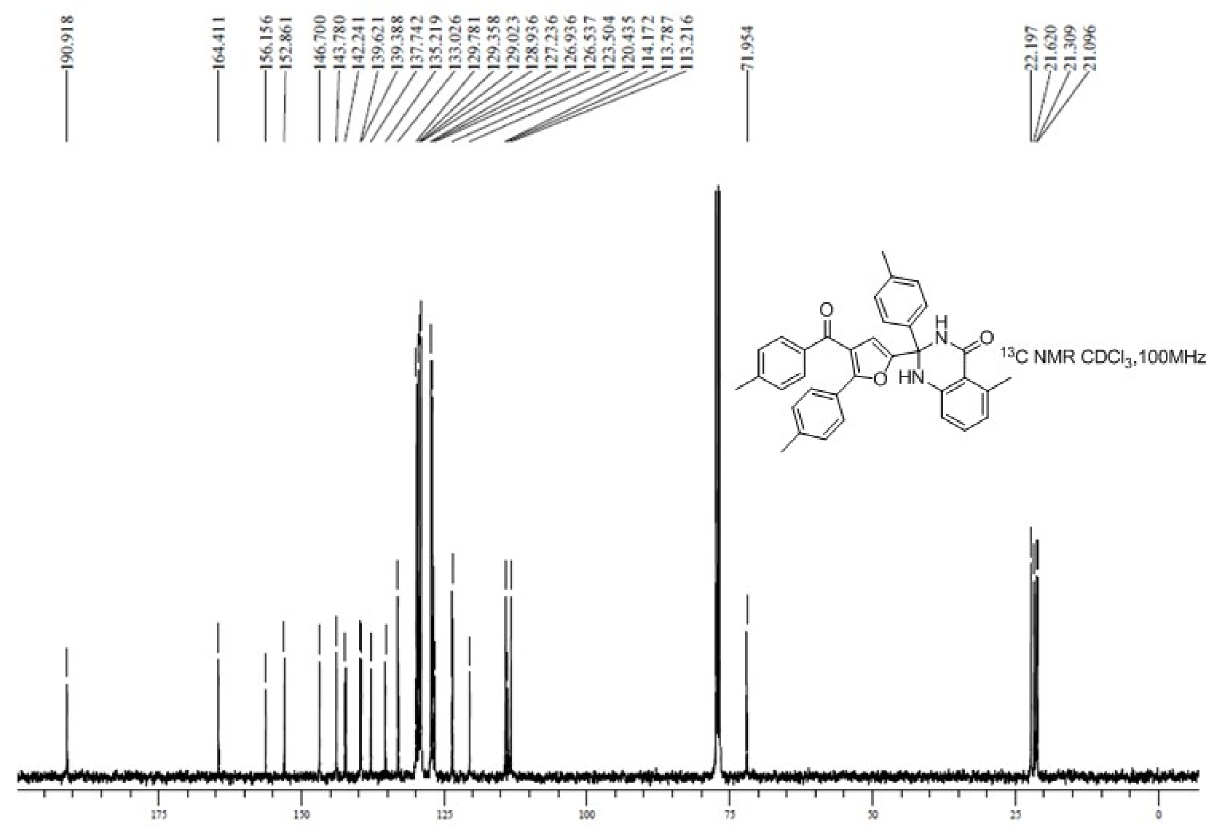

2-(4-Benzoyl-5-phenylfuran-2-yl)-2,7-diphenyl-5-(trifluoromethyl)-2,3-dihydropyrido[2,3-*d*]pyrimidin-4(*1H*)-one:**3x**

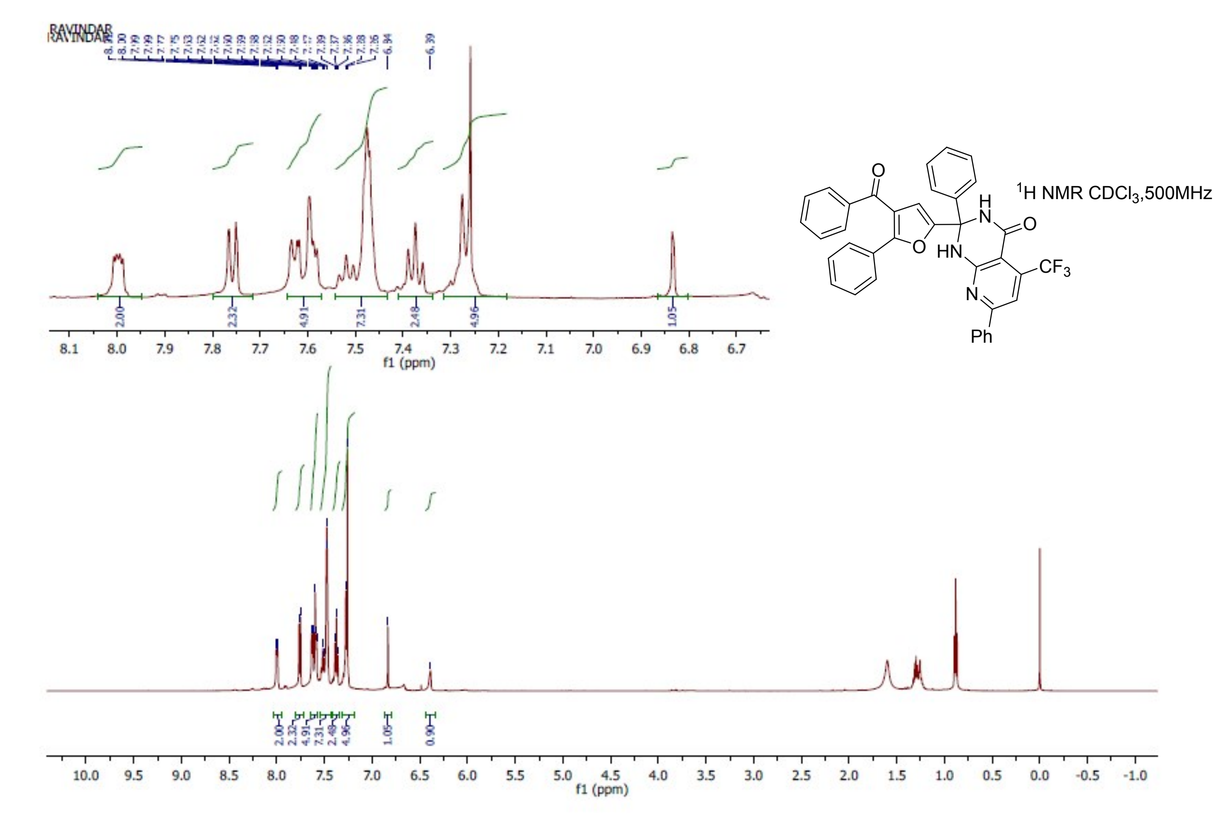

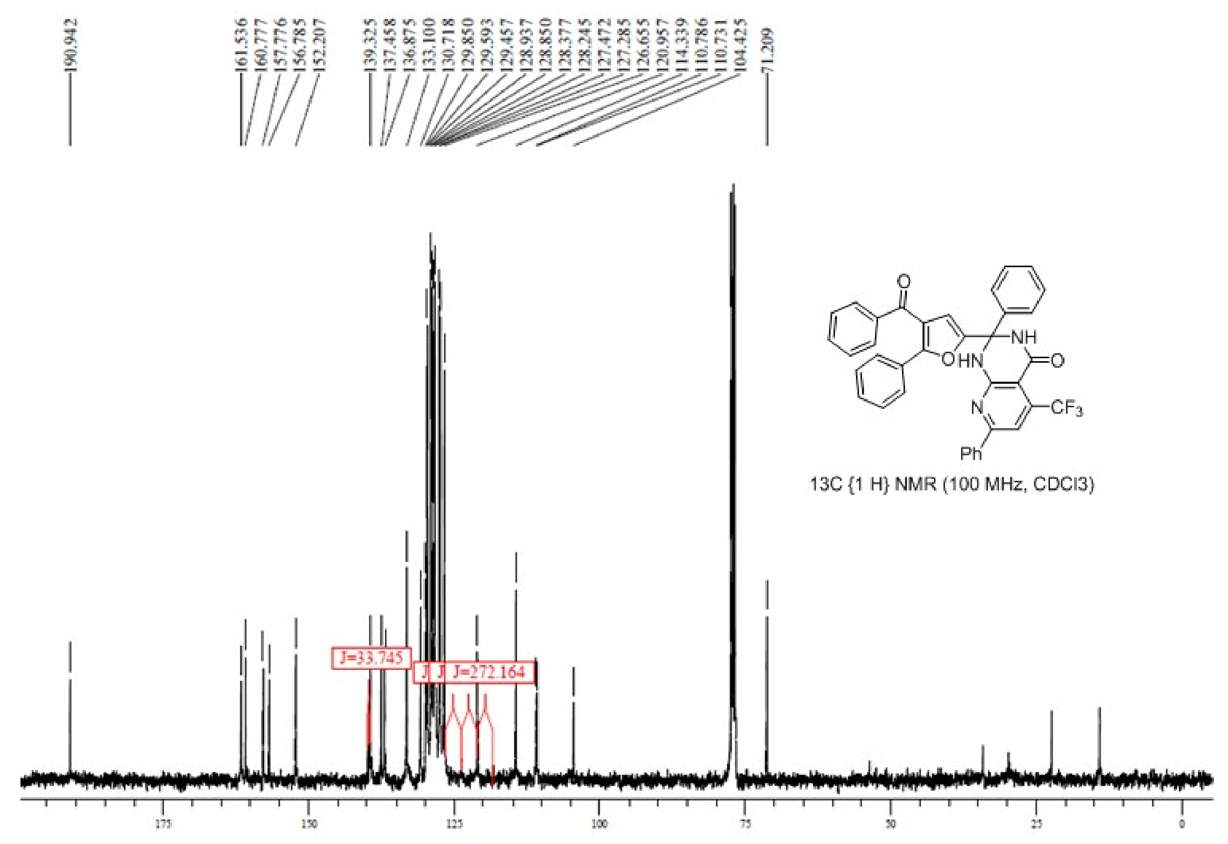

Dimethyl (2-methyl-5-(4-*oxo*-2-phenyl-1,2,3,4-tetrahydroquinazolin-2-yl)furan-3-yl)phosphonate: **3y**

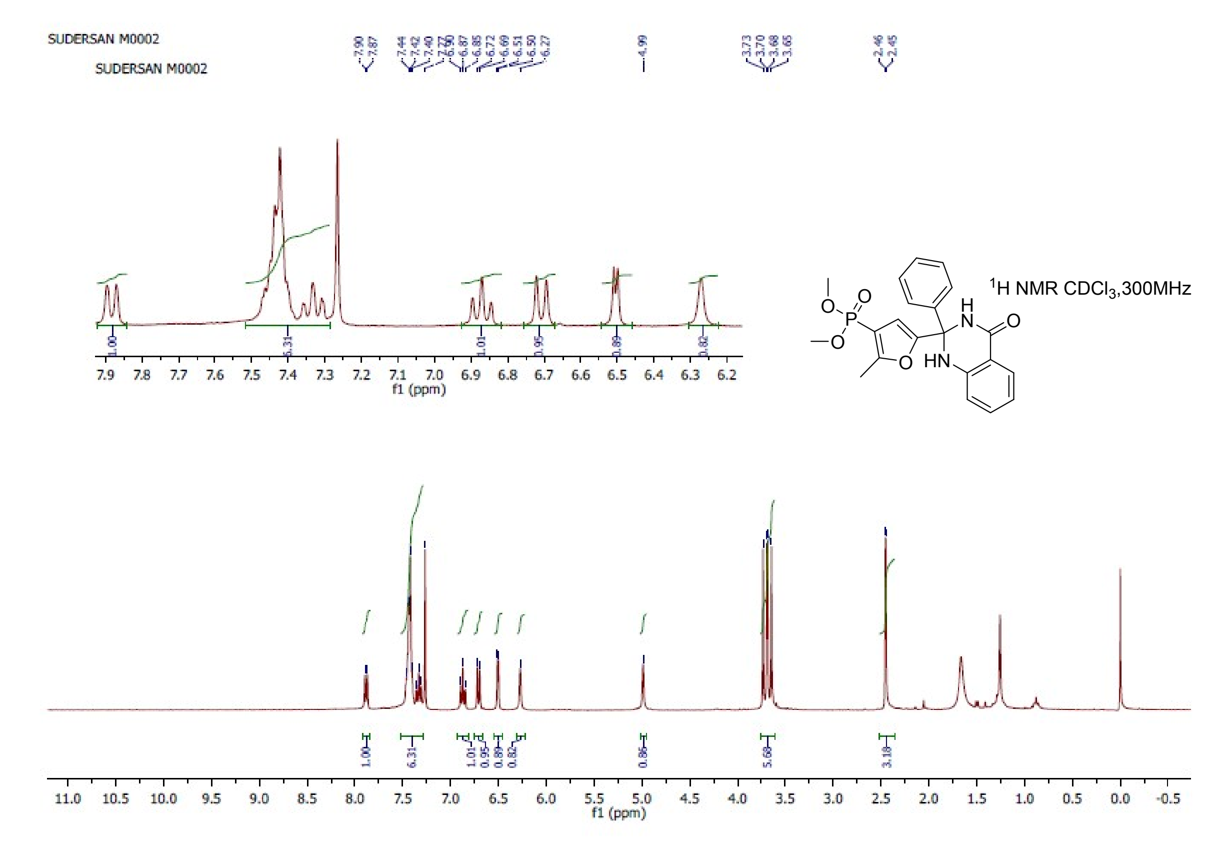

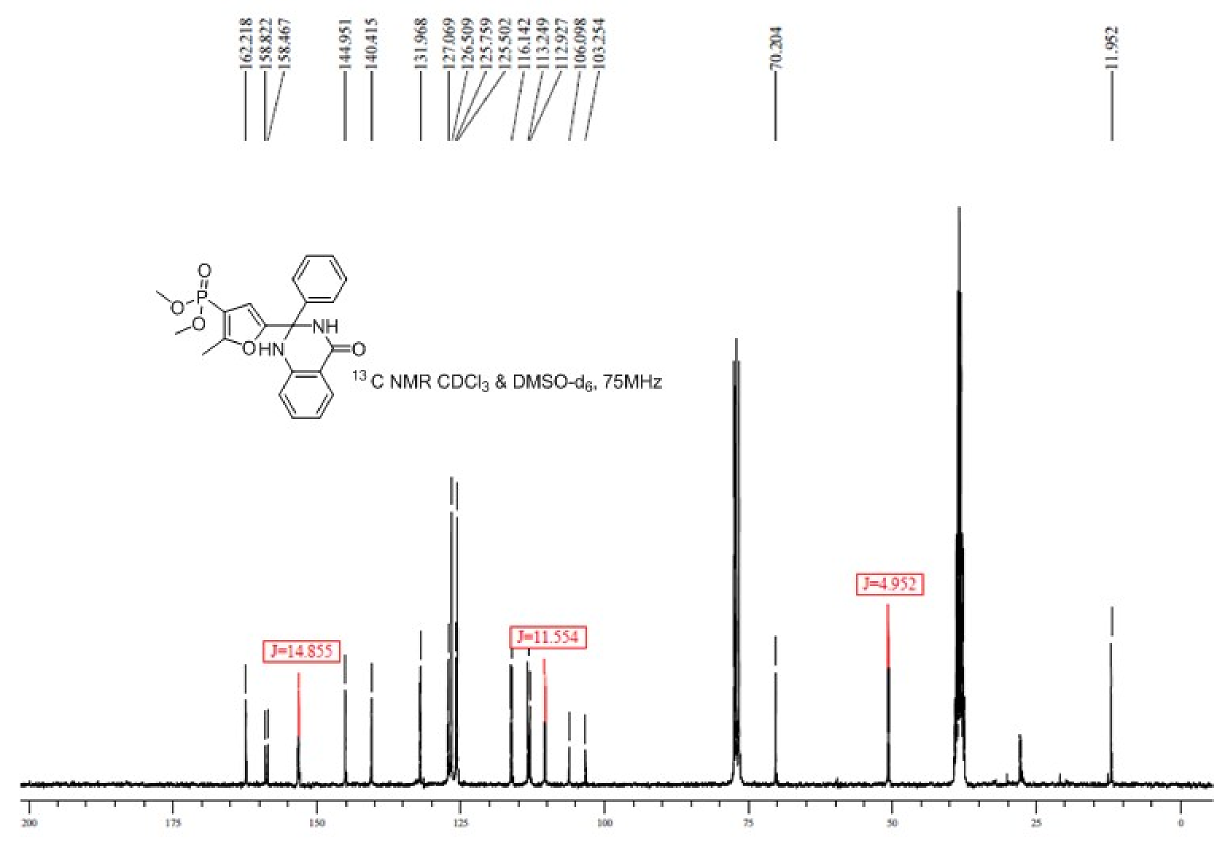

^{31}P NMR of compound **3y**

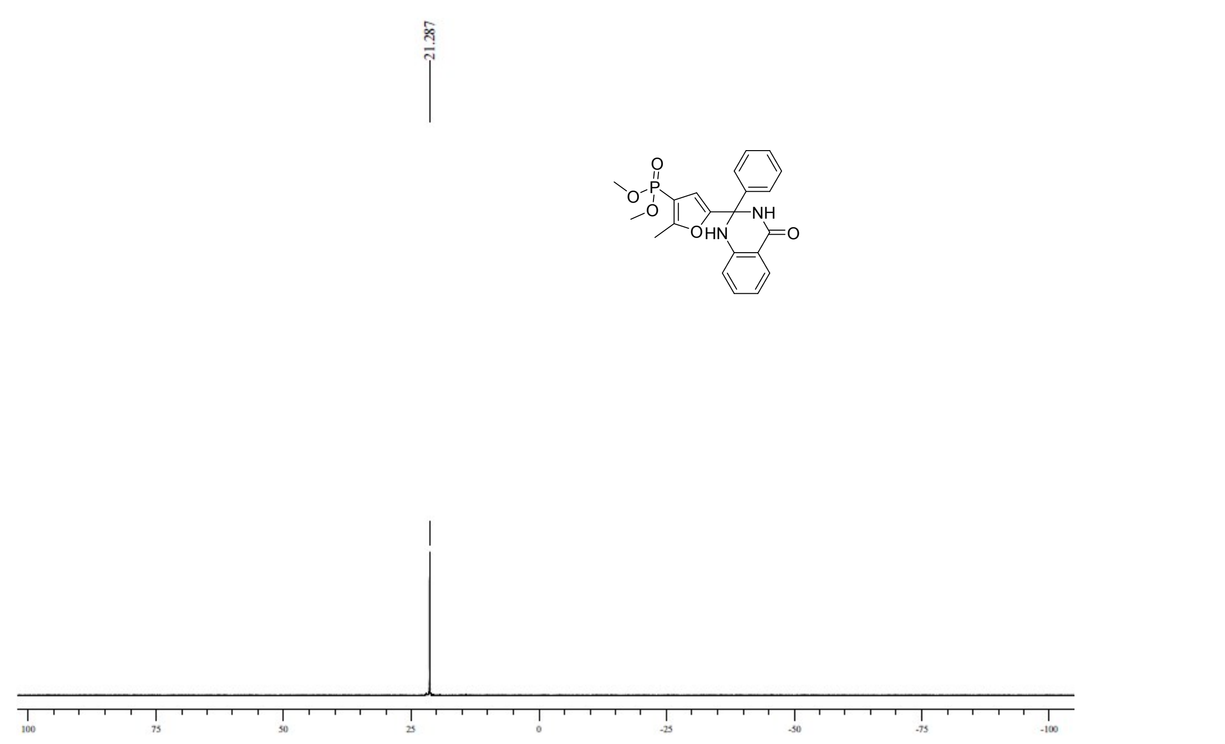

N'-((4-Benzoyl-5-phenylfuran-2-yl)(phenyl)methylene)-2-(prop-2-yn-1-yloxy)benzohydrazide:**5**

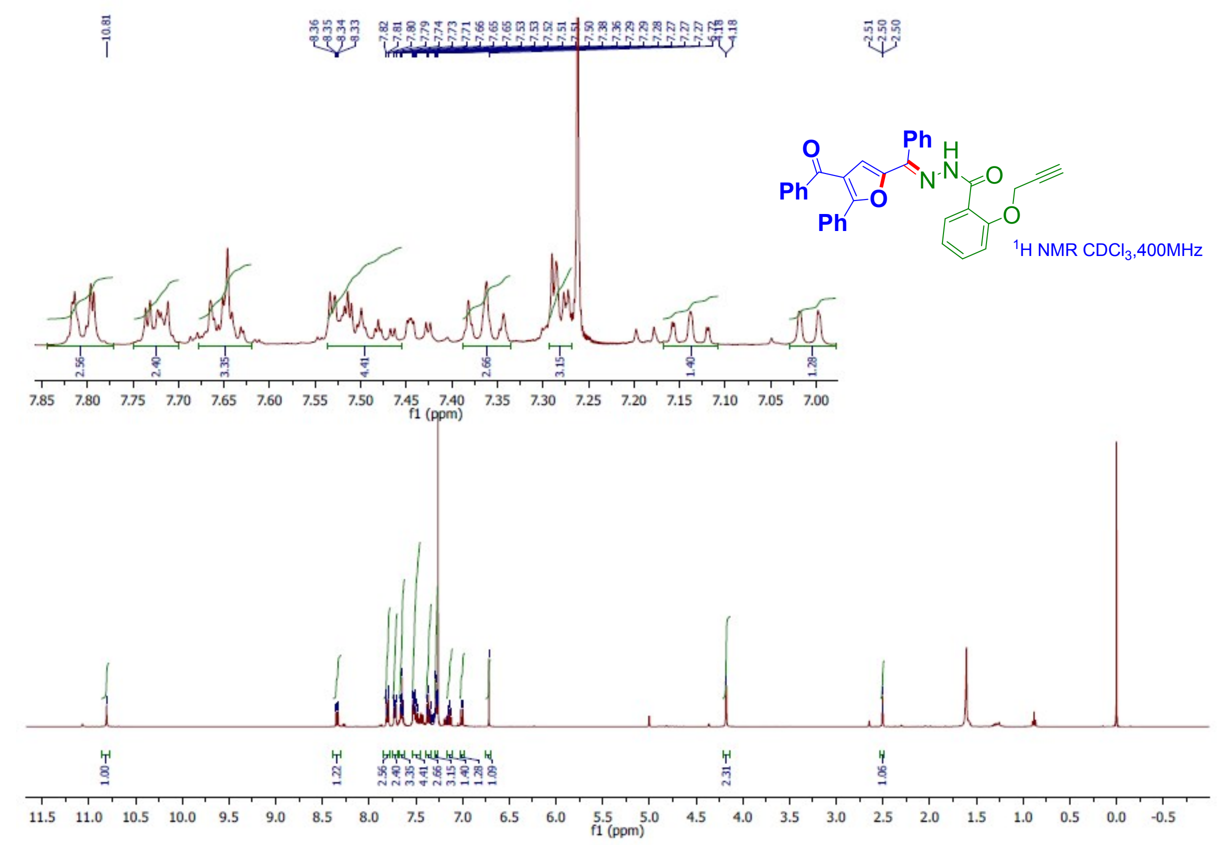

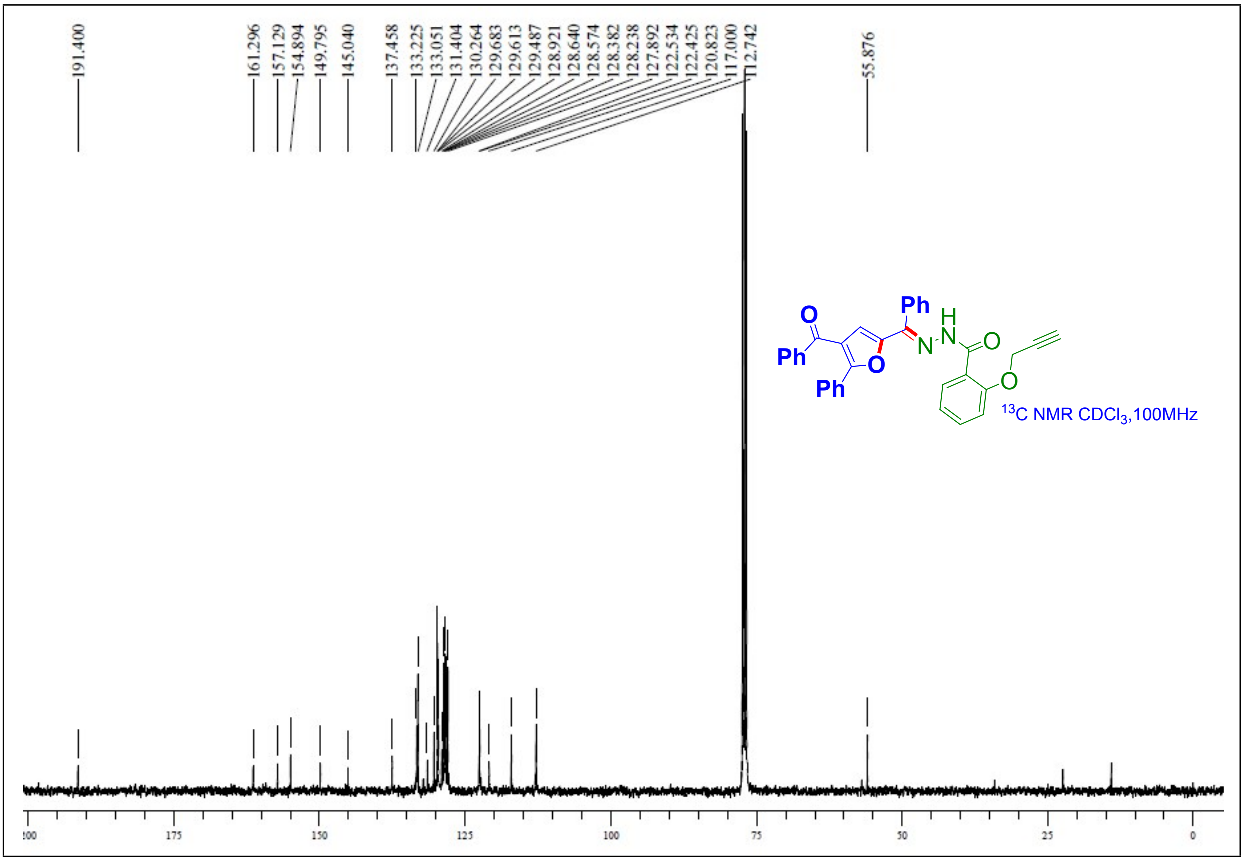
191.400
161.296
157.129
154.894
149.795
145.040
137.458
133.225
133.051
131.404
130.264
129.683
129.613
129.487
128.921
128.640
128.574
128.382
128.238
127.892
122.534
122.425
120.823
117.000
112.742
55.876
Ph
O
N
H
Ph
Ph
^{13}C NMR CDCl$_3$,100MHz
200
175
150
125
100
75
50
25
0

2 X-ray crystallography data of (2.1) compound 3a and (2.2) compound 5.

2.1 Crystal data for compound 3a

X-ray data for the compound KA302 was collected on a Bruker D8 QUEST instrument with an IμS Mo microsource (λ = 0.7107 A) and a PHOTON-100 detector. The raw data frames were reduced and corrected for absorption effects using the Bruker Apex 3 software suite programs.[1] The structure was solved using intrinsic phasing method[2] and further refined with the SHELXL[2] program and expanded using Fourier techniques. Anisotropic displacement parameters were included for all non-hydrogen atoms.The N-bound H atoms were located in difference Fourier maps, and their positions and isotropic displacement parameters were refined All C bound H atoms were positioned geometrically and treated as riding on their parent C atoms [C-H = 0.93-0.97 Å, and $U_{iso}(H) = 1.5U_{eq}(C)$ for methyl H or $1.2U_{eq}(C)$ for other H atoms].

Crystal Datafor KA302: $C_{31}H_{22}N_2O_3$ (M =470.50 g/mol): triclinic, space group P-1 (no. 2), a = 8.2197(2) Å, b = 10.4608(2) Å, c = 14.4752(3) Å, α = 77.0948(8)°, β = 79.6924(8)°, γ = 81.5655(9)°, V = 1186.21(4) Å^3, Z = 2, T = 294.15 K, μ(MoKα) = 0.085 mm^{-1}, $Dcalc$ = 1.317 g/cm^3, 35683 reflections measured (4.464° ≤ 2Θ ≤ 61.018°), 7216 unique (R_{int} = 0.0618, R_{sigma} = 0.0518) which were used in all calculations. The final R_1 was 0.0634 (I >2σ(I)) and wR_2 was 0.1669 (all data). CCDC1863534 contains supplementary Crystallographic data for the structure. These data can be obtained free of charge at www.ccdc.cam.ac.uk/conts/retrieving.html [or from the Cambridge Crystallographic Data Centre (CCDC), 12 Union Road, Cambridge CB2 1EZ, UK; fax: +44(0) 1223 336 033; email: deposit@ccdc.cam.ac.uk].

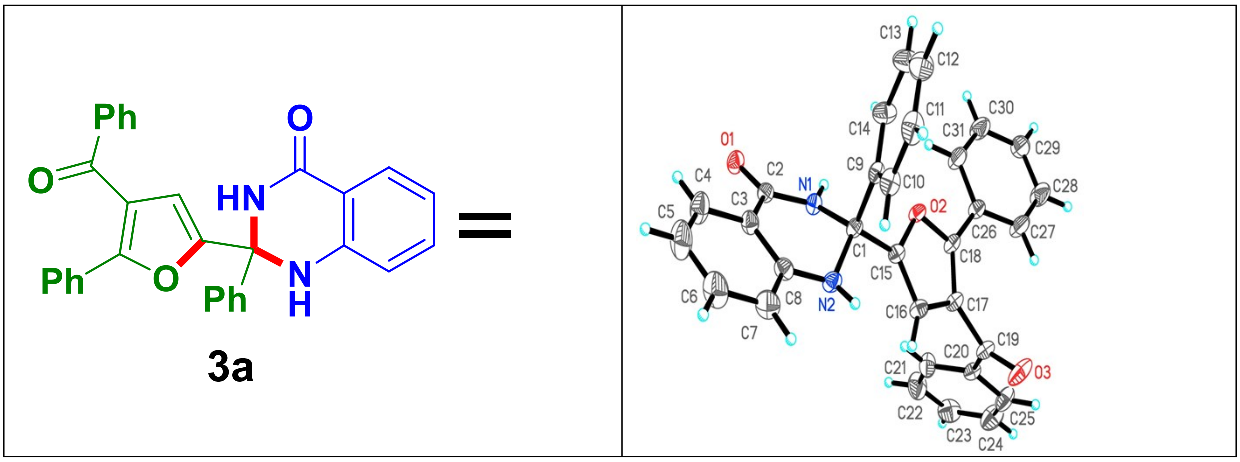

Fig.1. A view of **KA302**, showing the atom-labelling scheme. Displacement ellipsoids are drawn at the 30% probability level and H atoms are represented by circles of arbitrary radii. The Compound **3a** was

dissolved in ethanol and heated to 80 °C for 5 minutes and filtered. This solution was kept for 20 days at 28 °C, which leads to formation of single crystals.

2.2 Crystal data for compound 5

X-ray data for the compounds were collected at room temperature using a Bruker Smart Apex CCD diffractometer with graphite monochromatedMoKα radiation (λ=0.71073Å) with ω-scan method.[1] Preliminary lattice parameters and orientation matrices were obtained from four sets of frames. Integration and scaling of intensity data were accomplished using SAINT program.[1] The structure was solved by direct methods using SHELXS[2] and refinement was carried out by full-matrix least-squares technique using SHELXL.[2] Anisotropic displacement parameters were included for all non-hydrogen atoms.The N-bound H atoms and O-bound H atoms were located in difference Fourier maps and their positions and isotropic displacement parameters were refined. All other H atoms were positioned geometrically and treated as riding on their parent C atoms [C-H = 0.93-0.97 Å and $U_{iso}(H) = 1.5U_{eq}(C)$ for methyl H or $1.2U_{eq}(c)$ for other H atoms]. The methyl groups were allowed to rotate but not to tip.

Crystal Data for BG03: $C_{70}H_{53}N_4O_9$ (M =1066.17 g/mol): triclinic, space group P-1 (no. 2), a = 9.1182(6) Å, b = 13.8291(10) Å, c = 23.4126(16) Å, α = 103.581(1)°, β = 91.205(2)°, γ = 98.758(2)°, V = 2831.3(3) Å^3, Z = 2, T = 294.15 K, μ(Mo Kα) = 0.084 mm^{-1}, $Dcalc$ = 1.2505 g/cm^3, 35814 reflections measured (1.8° ≤ 2Θ ≤ 50°), 9961 unique (R_{int} = 0.0944, R_{sigma} = 0.1499) which were used in all calculations. The final R_1 was 0.1271 (I>2σ(I)) and wR_2 was 0.3437 (all data). CCDC 1898773 contains supplementary Crystallographic data for the structure. These data can be obtained free of charge at www.ccdc.cam.ac.uk/conts/retrieving.html [or from the Cambridge Crystallographic Data Centre (CCDC), 12 Union Road, Cambridge CB2 1EZ, UK; fax: +44(0) 1223 336 033; email: deposit@ccdc.cam.ac.uk].

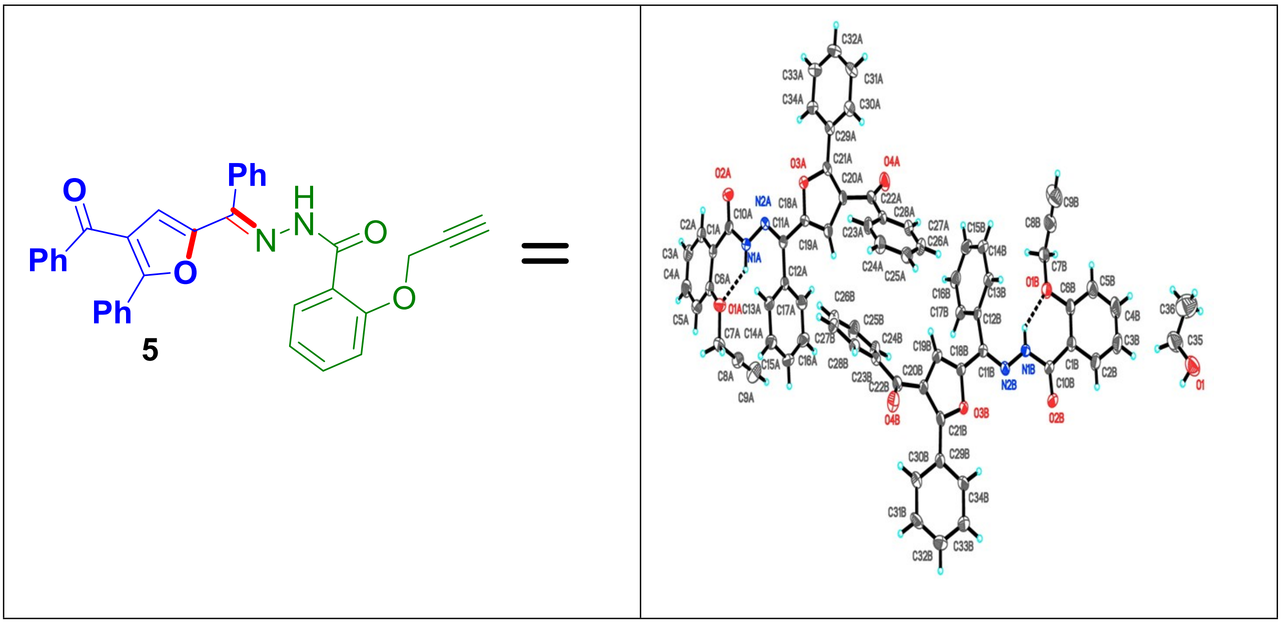

Fig.2. A view of BG03, showing the atom-labelling scheme. Displacement ellipsoids are drawn at the 30% probability level and H atoms are represented by circles of arbitrary radii. Hydrogen bonds are shown by dashed lines. The compound **8** was dissolved in ethanol and heated to 80 °C for 5 minutes and filtered. This solution was kept for 15 days at 28 °C, which leads to formation of single crystals.

Reference:

1. Bruker (2001). SAINT (Version 6.28a) &SMART (Version 5.625). Bruker AXS Inc., Madison, Wisconsin, USA.
2. Sheldrick G. M. (2015**)** ActaCrystallogrC71:3-8.
